# Impact of Chelation on Reactivity and Cytotoxicity of Hemilabile Biphenyl Gold(III) N‐Heterocyclic Carbene Complexes

**DOI:** 10.1002/cmdc.202500302

**Published:** 2025-06-14

**Authors:** Tom Lacoma, Jérémy Forté, Régina Maruchenko, Romain Morichon, Michèle Salmain, Joëlle Sobczak‐Thépot, Benoît Bertrand

**Affiliations:** ^1^ Institut Parisien de Chimie Moléculaire (IPCM) Sorbonne Université CNRS 4 place Jussieu F‐75005 Paris France; ^2^ Centre de Recherche Saint Antoine (CRSA) Sorbonne Université, INSERM 184 rue du Faubourg Saint Antoine F‐75 012 Paris France

**Keywords:** cytotoxicity, gold, hemilabile, organometallic, reactivity

## Abstract

Although great progresses have been accomplished in the field of antineoplastic treatments, the need for chemotherapy agents with new mechanisms of action remains essential. Metal complexes presenting hemilabile ligands could combine structural toxicity upon full coordination of the ligand and reactive toxicity upon ligand partial decoordination and direct coordination of the metal center to biological targets. To investigate the relevance of hemilability in the case of Au(III) complexes, we synthesized eight open biphenyl gold(III) N‐heterocyclic carbene complexes coined **BGC** of general formula [(C^C)Au(NHC^het)Cl] where het is a pyridine‐type entity and C^C is 4,4′‐diterbutylbiphenyl. Chloride abstraction afforded the chelated cationic complexes [(C^C)Au(NHC^N)]PF_6_ in which the pyridine arm coordinates the gold ion. Quantitative irreversible conversion of the cationic forms to the neutral ones in the presence of chloride ions was demonstrated through extensive speciation studies by ^1^H NMR spectroscopy on both forms in different media including DMSO/cell culture medium mixture. The **BGC** complexes exhibited antiproliferative activity in the low micromolar range with equivalent activities for each open neutral/chelated cationic pair. Time lapse fluorescence videomicroscopy studies demonstrated the activation of effector caspases 3/7, suggesting the induction of apoptosis. Preliminary mechanistic studies suggest that apoptotic cell death may arise partially from mitochondrial membrane depolarization.

## Graphical abstract

An array of eight pairs of biphenyl gold(III) complexes comprising NHC–pyridine ligands having a hemilabile character in vitro displays antiproliferative activity toward human cells in the low micromolar range which is exclusively due to their neutral open form according to speciation studies. All the complexes induce mitochondrial membrane depolarization, likely succeeding to apoptotic cell death.

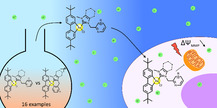

## 1 Introduction

Platinum‐based compounds are major components of the arsenal against cancer, as they are still present in the majority of chemotherapeutic cocktails.^[^ ^1^, ^2^ ^]^ However, resistance phenomena, both intrinsic and acquired, and the associated side‐effects narrow down the range of applicability of these treatments.^[^ ^3^ ^]^ This highlights the need for new therapeutic strategies to overcome platinum drugs limitations. One strategy consists in the replacement of Pt(II) by other transition metals.^[^ ^4^, ^5^, ^6^, ^7^, ^8^ ^]^ In this context, Au(I/III) complexes have been extensively studied since the 80's as potential alternatives of platinum‐based drugs due to the isoelectronic and isostructural character of Au(III) and Pt(II).^[^ ^5^, ^6^, ^9^, ^10^, ^11^ ^]^ Interestingly, while Pt(II) complexes are reported to induce cytotoxic effects through the direct coordination of the Pt(II) ion to puric bases of DNA,^[^ ^12^ ^]^ Au(I/III) compounds were found to trigger their anticancer effects mostly via enzyme inhibition,^[^ ^13^, ^14^ ^]^ although anticancer activity through G4‐DNA stabilization has been reported in some cases.^[^ ^15^, ^16^, ^17^ ^]^ Gold‐induced enzyme inhibition can be achieved through two different mechanisms involving or not the direct coordination of the gold ion by the target enzyme(s). Reactive toxicity involves the direct coordination of the Au(I/III) ion by S‐ or Se‐containing amino acids such as cysteine,^[^ ^18^, ^19^ ^]^ methionine,^[^ ^20^ ^]^ and selenocysteine^[^ ^21^, ^22^, ^23^ ^]^ requiring the presence of exchangeable ligands such as halogenides, thiolates, or carboxylates.^[^ ^13^, ^24^ ^]^ On the contrary, structural toxicity is induced by the interaction of the unmodified structure with biological targets via noncovalent interactions such as electrostatic, hydrophobic interactions or *π*‐stacking, which requires stable ligands such as cyclometalated, N‐heterocyclic carbenes (NHC) or diphosphine ligands.^[^ ^25^, ^26^, ^27^ ^]^

**Figure 1:**
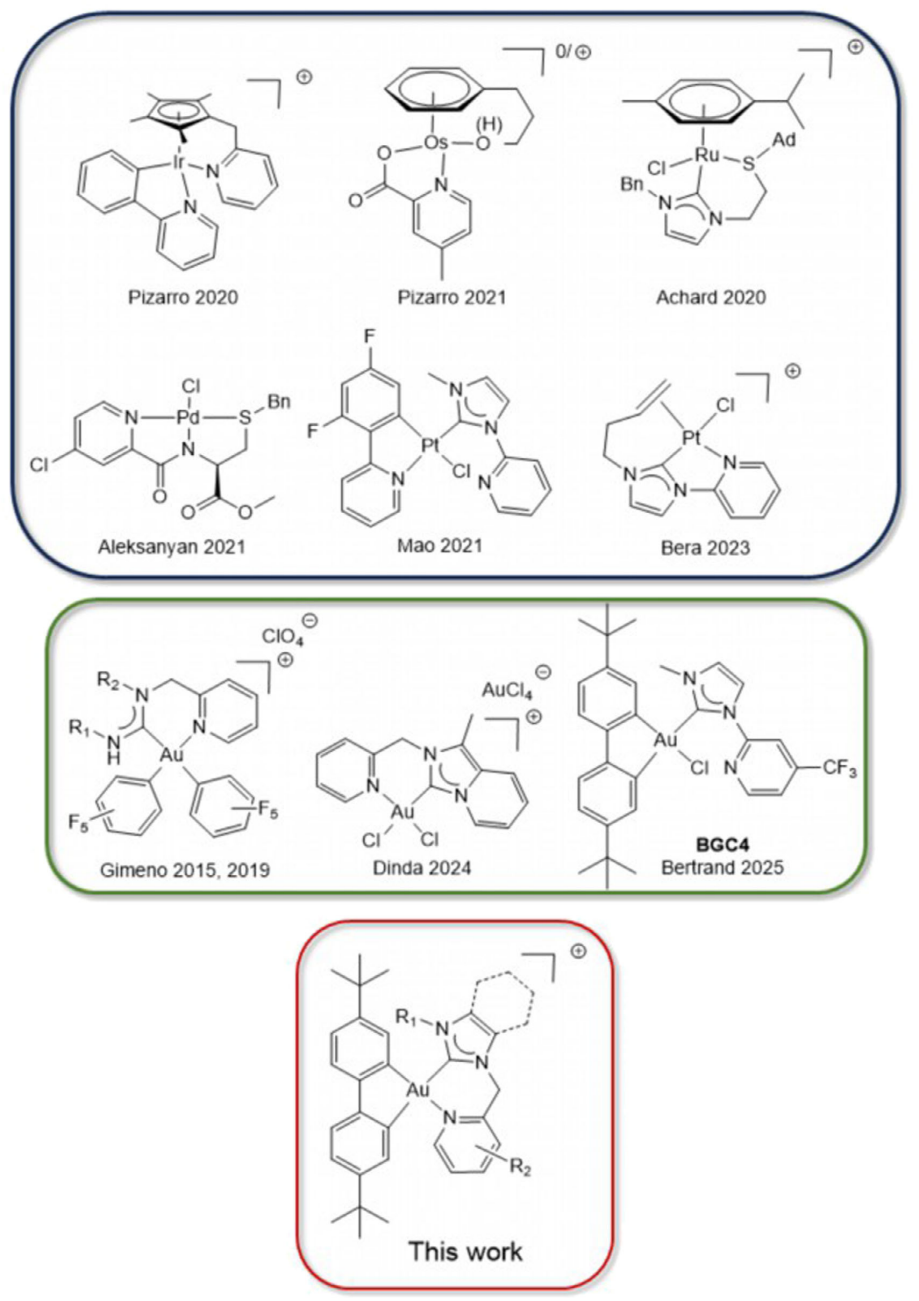
Examples of metal complexes with hemilabile ligands reported for biological applications, structure of complex **BGC4**, and general structure of the Au(III) complexes with hemilabile ligands reported herein.

In this context, the design of complexes able to trigger both reactive and structural toxicities might be of major relevance to broaden the scope of potential targets for a given compound. This aim can be achieved with hemilabile ligands presenting a stable ligand on one side and a weakly bound one on the other side that can coordinate or not the metal center. This concept has been recently successfully applied to various metals including Ir(III),^[^ ^28^, ^29^ ^]^ Os(II),^[^ ^30^ ^]^ Ru(II),^[^ ^31^ ^]^ Pd(II),^[^ ^32^ ^]^ and Pt(II)^[^ ^33^, ^34^ ^]^ (**Figure** **1**). In particular, Pizarro and coworkers reported pyridine‐tethered Cp* Ir(III) complexes up to 100 times more cytotoxic to breast cancer cells compared to the analogous complexes with separated ligands, due to the stronger coordination of the pyridine ligand induced by the chelating effect.^[^ ^28^ ^]^ Mao reported the intracellular cyclization of an NHC‐pyridine ligand to afford a square planar cationic [(C^N)Pt(NHC^N)]^+^ structure, through stabilization of G4‐structures of DNA, which could efficiently inhibit cancer cell growth.^[^ ^33^ ^]^ However, although the catalytic^[^ ^35^, ^36^ ^]^ and anticancer properties^[^ ^37^, ^38^, ^39^ ^]^ of some Au(III) complexes with chelating (carbene^pyridine) ligands have already been reported, the potential hemilabile behavior of these systems has not been investigated.

Recently, biphenyl‐based organogold(III) complexes presenting two Au—C bonds associated with bidentate diimine^[^ ^40^ ^]^ or diphosphine ligands^[^ ^27^, ^41^, ^42^ ^]^ have demonstrated a high redox stability, even in the presence of the biological reductant glutathione (GSH). Some of us have recently reported a family of eleven biphenyl gold N‐heterocyclic carbene complexes (**BGC** family) presenting a pyrido‐NHC ligand. In particular, complex **BGC4** (Figure 1) appeared particularly cytotoxic to “triple negative” breast cancer cells, both in vitro and in vivo. Although structurally analogous to Mao's Pt(II) complexes, the involvement of the cyclized [(C^C)Au(C^N)]^+^ form to the anticancer activity was ruled out.^[^ ^43^ ^]^ In order to favor the coordination of the pendant pyridine to give the cationic [(C^C)Au(C^N)]^+^ form, we undertook the synthesis of **BGC** complexes with 2‐picolyl‐NHC ligands (Figure 1). The addition of a methylene spacer is known to lead to the formation of more stable 6‐member metallacycles upon ring closure while moving away the pyridine from the electron‐withdrawing NHC ligand.^[^ ^35^, ^37^, ^38^, ^39^ ^]^ We have extended the **BGC** family with 16 new neutral and cationic complexes for which the substituent on the pyridine ring, the nature of the heterocycle as well as the nature of the NHC ligand have been varied. The speciation of the different complexes alone and in the presence of chloride ions was investigated by ^1^H NMR in DMSO, DMSO/D_2_O and DMSO/DMEM. The antiproliferative properties of the complexes have been measured in vitro and compared to analogs with separated NHC and pyridine ligand and **BGC4**. Investigations of the mode of action of this new family of complexes including reactivity studies with histidine, methionine and cysteine, and effects of the complexes on mitochondria were carried out, establishing a strong proof‐of‐concept of the use of Au(III) complexes with hemilabile ligands for anticancer applications.

## 2 Results and Discussion

### 2.1 Synthesis and Characterization

**Scheme 1:**
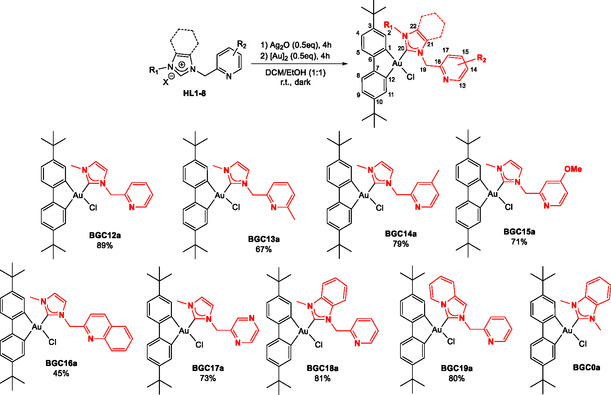
Synthesis of the neutral [(C^C)Au(NHC^2‐pic)Cl] complexes **BGC12a**‐**19a** and structure of complex **BGC0a**.

(2‐Picolyl)azolium salts (imidazolium, benzimidazolium, and pyridoimidazolium salts) **HL1‐8** were synthesized from the corresponding azoles and variously substituted 2‐picolylhalides (bromide or chloride) and analogs according to a reported procedure.^[^ ^44^ ^]^ By slight modification of the procedure applied to the synthesis of the first series of biphenyl gold NHC complex (**BGC0a**),^[^ ^45^ ^]^ a series of eight neutral biphenyl gold NHC complexes of general formula [(C^C)Au(NHC‐het)Cl] were obtained from the corresponding azolium pro‐ligands (**scheme** **1**, **BGC12a**‐**19a**) through a two‐step procedure involving silver carbene complex generation from the corresponding azolium salts reaction with silver oxide and subsequent transmetallation to the bisphenyl‐gold(III) dimeric precursor [Au]_2_. All compounds were isolated in moderate to very good yields after purification by silica gel column chromatography.

**Scheme 2:**
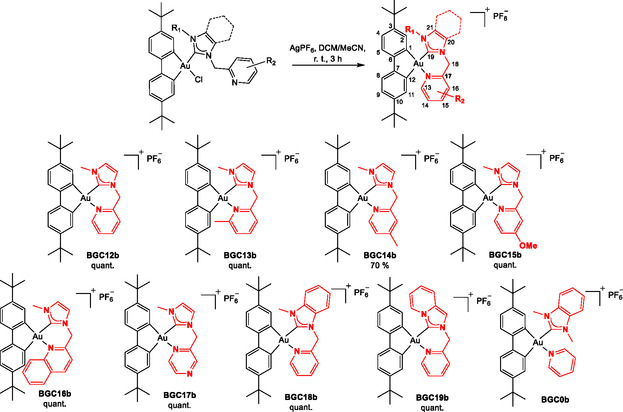
Synthesis of the cationic [(C^C)Au(NHC^N)]PF_6_ complexes **BGC12b**‐**BGC19b** and structure of complex **BGC0b**.

Using a reported procedure for the synthesis of [(C^C)Au(NHC)(pyr)]^+^ complexes,^[^ ^45^ ^]^ the neutral complexes **BGC12a**‐**19a** were readily converted to their cyclic cationic analogs by addition of AgPF_6_ affording a second series of eight cationic [(C^C)Au(NHC^N)]PF_6_ complexes in high to quantitative yields (**scheme** **2**, **BGC12b**‐**19b**). The syntheses were performed with variously substituted 2‐picolines (**BGC12**‐**15**), heterocycles (**BGC12**,** 16, 17**), or carbenes (**BGC12**, **18, 19**) while keeping good yields, demonstrating a broad tolerance to electron‐donating or ‐withdrawing groups, and to steric hindrance.

The compound structures were determined by ^1^H and ^13^C{^1^H} NMR and further confirmed by HRMS analysis; their purity was assessed by elemental analysis. One notable feature of the complexes’ ^1^H NMR spectra is the characteristic doublet corresponding to proton H^13^ of the 2‐picoline, which is shifted upfield by 0.40 to 0.49 ppm as the pyridine ring coordinates to gold. This proton is thus a good probe to monitor under which form (neutral open or cationic cyclized form) the complexes are present under given conditions. Moreover, upon coordination of the pyridine ring, a downfield shift of 1 ppm is observed for the signal of proton H^11^ due to the anisotropy cone of the aromatic pyridine rings. This downfield shift even reaches 2 ppm in the case of the quinoline complex **BGC16b** due to the extended *π* surface. This has already been observed in the case of analogous chelating or bridging diphosphine complexes.^[^ ^41^, ^46^ ^]^ Oppositely, the signal of H^2^ appeared shifted upfield by up to 1.3 ppm (with exception of complexes **BGC16a/b**) upon coordination of the heterocycle. This might arise from the geometrical constraint induced by the chelation reducing the interaction of this proton with the π system of the facing NHC.

### 2.2 Crystal Structure Analysis

**Figure 2:**
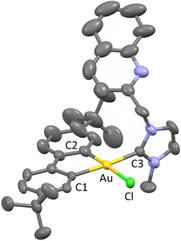
Solid‐state structure of one of the two molecules of the asymmetric unit of **BGC16a**. Ellipsoids set at 50% probability. Hydrogen atoms and solvent molecule have been omitted for clarity. Selected bond distances [Å] and angles [°] measured at 200 K: Au‐C_1_ 2.058(4), Au‐C_2_ 2.021(4), Au‐C_3_ 2.079(4), and Au‐Cl 2.3847(10) and C_1_‐Au‐C_2_ 81.37(17), C_2_‐Au‐C_3_ 93.50(17), C_3_‐Au‐Cl 88.45(12), and Cl‐Au‐C_1_ 96.71(12).

Crystals suitable for X‐ray diffraction were grown for neutral complexes **BGC12a**, **BGC16a**, and **BGC18a** and for cationic complexes **BGC13b**, **BGC16b**, and **BGC18b** by slow evaporation of a concentrated solution in a DCM/PE (2:1 v/v) mixture, and their solid‐state structures were elucidated by X‐ray diffraction (XRD) analysis (**Figure** **2** **,** **3** and S1–4, Supporting Information). Solid‐state structures of complexes **BGC12a**, **BGC16a**, and **BGC18a** confirmed the neutral structure with coordination of the chlorido ligand and pendant pyridine and quinoline respectively. In all cases, a barely distorted square‐planar geometry was observed with distances, in particular Au‐C_3_ in the same range as those of reported [(C^C)Au(NHC)Cl] complexes (Figure 2 and S1–2, Supporting Information).^[^ ^43^, ^45^, ^47^ ^]^

**Figure 3:**
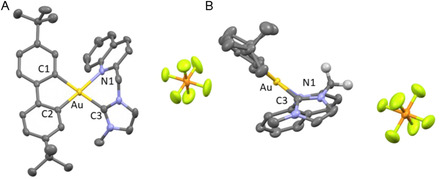
Solid‐state structure of **BGC16b**. Ellipsoids set at 50% probability. Hydrogen atoms and solvent molecule have been omitted for clarity. Selected bond distances [Å] and angles [°] measured at 200 K: Au‐C_1_ 2.047(4), Au‐C_2_ 2.025(4), Au‐C_3_ 2.065(4), and Au‐N_1_ 2.169(3) and C_1_‐Au‐C_2_ 88.83(15), C_2_‐Au‐C_3_ 99.25(15), C_3_‐Au‐N_1_ 82.09(14), and N_1_‐Au‐C_1_ 97.69(13). A) Top view. B) Side view.

Solid‐state structures of cationic complexes **BGC13b**, **BGC16b**, and **BGC18b** confirmed the coordination of the nitrogen atom of the pyridine, *o*‐methylpyridine, and quinoline respectively with a slightly distorted square‐planar geometry (Figure 3A and S3‐4, Supporting Information). The 6‐membered ring of the (NHC^N) chelate adopts a pseudo‐boat configuration in good agreement with previously reported crystallographic structures for (C^N)Au^III^ complexes.^[^ ^35^, ^37^, ^38^, ^39^, ^48^ ^]^ Compared to the neutral complexes, no significant modification of the Au‐C_3_ bond length could be observed. In the same way, chelation of (NHC^N) ligand led to Au‐C_3_ and Au—N bond lengths similar to those of complexes with separated NHC and pyridine ligands.^[^ ^45^ ^]^

### 2.3 Log P Measurements

The water/octanol partition coefficient (log P) of complexes **BGC12‐19a/b** was assessed as an indicator of their lipophilicity, which has a strong impact on their biodistribution and availability. The data can be found in Table S2, Supporting Information, and show log P ranging from 4.4 to 5.7 for **BGC12‐19a**, and from 3.5 to 4.8 for **BGC12‐19b**, each neutral complex being more lipophilic than its cationic counterpart differing by 0.5 to 1.2 log P units. Interestingly, the separation between the NHC and pyridine parts by a methylene bridge seems to decrease the log P value of neutral complexes **BGC12‐19a** in comparison to the previous generation of **BGC** complexes, which showed a much higher lipophilicity with log P values between 7 and 8.1.^[^ ^43^ ^]^ This can be tentatively rationalized by considering the higher basicity of the heterocycles due to their isolation from the NHC part. Overall, the highly lipophilic nature of complexes **BGC12‐19a/b** is expected to warrant cell permeability.

### 2.4 Speciation in Solvents and Culture Medium

**Figure 4:**
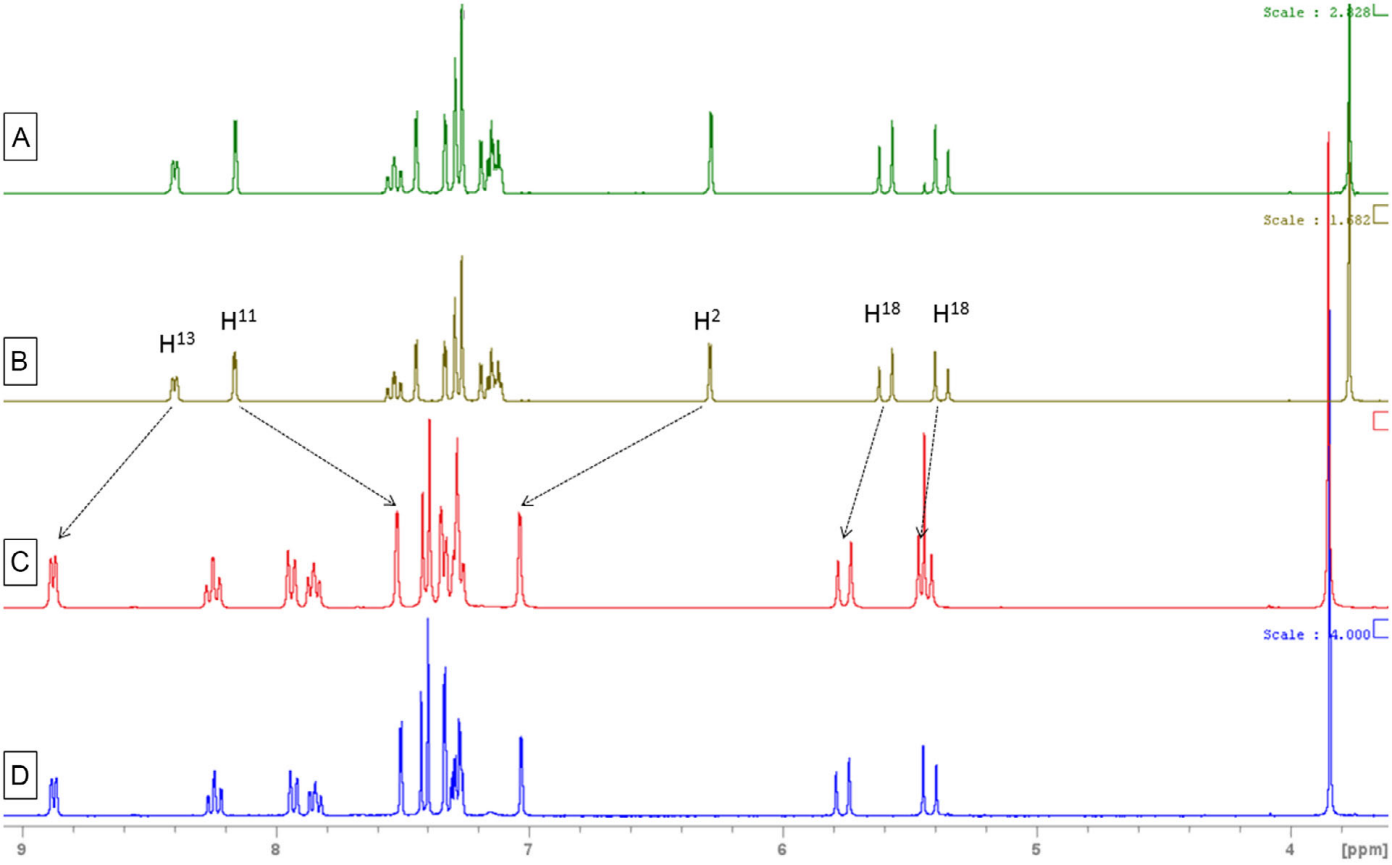
Spectra in MeCN‐d_3_ of A) **BGC12a** after synthesis. B) **BGC12a** after 24 h incubation at 37 °C in DMSO and extraction in DCM. C) **BGC12b** after synthesis. D) **BGC12b** after 24 h incubation at 37 °C in DMSO and extraction in DCM.

Complexes **BGC12**‐**19a**/**b** were tested for their stability in DMSO‐d_6_ and DMSO‐d_6_/D_2_O mixture by ^1^H NMR spectroscopy at a concentration of 5 mM. No modification of the spectra of all the complexes was observed in both solvents over a time range of 72 h at 37 °C, suggesting the stability of both neutral and cationic structures in these solvents (Figure S6–S37, Supporting Information). To ensure that the identified species result from an absence of reactivity and not from an instantaneous and quantitative reaction, spectra of **BGC12a** and **BGC12b** were recorded in MeCN‐d_3_ after 24 h incubation in DMSO followed by extraction of the products in DCM and evaporation. The spectra turned out unchanged compared to those of the initial products, confirming that no reaction occurred upon dissolution in DMSO (**Figure** **4**).

**Figure 5:**
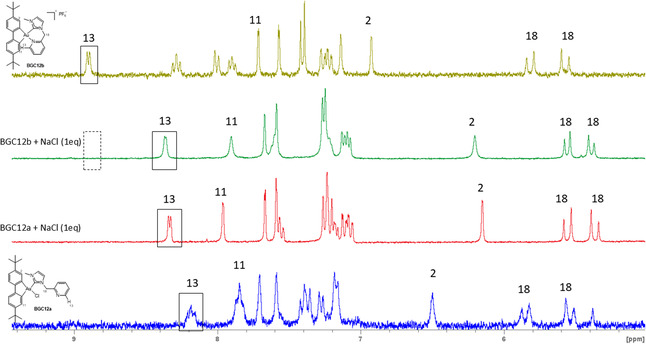
^1^H NMR spectra of **BGC12a** and **BGC12b** in DMSO‐d_6_/D_2_O 4/1 mixture with and without 1 equivalent of NaCl as Cl^−^ anion source.

Complexes **BGC12b**, **BGC15b**, **BGC19b**, and **BGC0b** were further probed for their reactivity toward chloride ions by ^1^H NMR in DMSO‐d6/D_2_O 4/1. Upon addition of 1 eq. of a chloride ions source, the aforementioned cationic complexes converted back quantitatively and instantaneously to their respective neutral form, thus proving the hemilabile nature of the present complexes (**Figure** **5** and S38–S40, Supporting Information). This series of results suggests that the Au—N bond strength is not modified by introducing a highly σ‐donating pyridine (**BGC12b** vs. **BGC15b**) or even by the use of a chelate ligand (**BGC12b** vs. **BGC0b**). This contrasts with the results obtained with Ir(III) complexes.^[^ ^28^ ^]^ Moreover, oppositely to what has been found for dichloroAu(III) complexes,^[^ ^35^, ^39^ ^]^ no sign of a backconversion from the neutral to the cationic species could be observed in the absence of external source of chloride ion (Figure 5, S21–28, Supporting Information and S37–39, Supporting Information).

**Figure 6:**
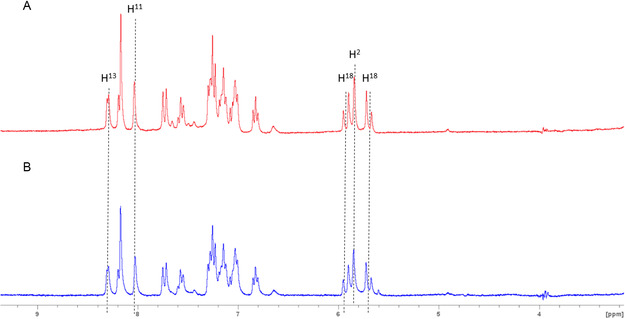
^1^H NMR spectra of A) **BGC19a** and B) **BGC19b** in DMSO‐d_6_/DMEM 3:1 mix using cpmg‐espg1d water suppression sequence (carrier frequency O1P is adjusted to the maximum of the water peak, Hahn echo delay D20 is left null).

The speciation of the gold complexes **BGC12a/b** and **BGC19a/b** was then further studied in a 3/1 DMSO‐d_6_/serum‐free DMEM mixture, to gain more insight into their behavior in biological‐like medium (**Figure** **6** and S41, Supporting Information). To run ^1^H NMR spectra in this complex medium, a particular ^1^H sequence (CPMG‐ESPG1D, see Experimental Part for details) was optimized in order to abolish the water signal, which led to a much higher signal/noise ratio. Already immediately after mixing (t < 5 min), for both couples, identical spectra were obtained for the neutral and cationic complexes which featured the signals of H^13^ at low chemical shifts (8.31 and 8.30 ppm respectively), the signals of H^11^ at high chemical shifts (7.96 and 8.03 ppm respectively), and the signals of H^2^ at low chemical shifts (6.12 and 5.69 ppm respectively). Therefore, it can be concluded that only the neutral complexes were identified for the two considered couples, indicating rapid conversion of the cationic complexes to the neutral ones, owing to the high concentration of chloride ions in the medium (c.a. 120 mM). This suggests that only the neutral form will interact with the cells, regardless of the initial form of the complex added to the culture medium.

### 2.5 In Vitro Antiproliferative Activity

**Figure 7:**
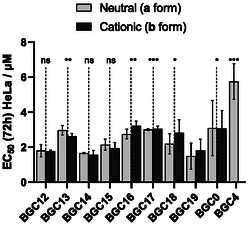
Antiproliferative activity of complexes **BGC12**‐**19a/b**, **BGC0a**/**b**, and **BGC4** against HeLa cells after 72 h incubation at 37 °C under 5% CO_2_; each data represents the average of three experiments ± standard error. Bilateral T‐test between **BGC12‐18a + b** (neutral a and cationic b forms pooled together) and **BGC19a + b** are represented above; n.s.: p > 0.05; *: 0.01 < p < 0.05; **: 0.001 < p < 0.01; ***: p < 0.001. Cellular viability measured through resazurin assay (fluorescence reading).

All new 16 **BGC** compounds were screened against HeLa cells (cervical adenocarcinoma) to assess their antiproliferative activity through a resazurin cell viability assay.^[^ ^49^ ^]^ The dose‐response curves were then fitted by a 4‐parameter sigmoid function to extract the median effective concentrations (EC_50_) shown in **Figure** **7**.

All complexes appeared highly active against HeLa cells with close EC_50_ values, ranging from 1.5 ± 0.7 to 3.2 ± 0.3 μM for **BGC19a** and **BGC16b** respectively, being all significantly more active than the previously reported complex **BGC4**. Moreover, no significant differences in toxicity could be observed between each neutral complex and its cationic counterpart whether the NHC and N‐donor ligands are linked (**BGC12‐19**) or not (**BGC0**). This is consistent with the speciation identified earlier, showing the immediate conversion of the cationic complexes (b series) to their neutral counterparts (a series) in the presence of chloride ions. In the same way, no difference of activity could be observed between the benzimidazole‐based (C^N) complexes **BGC18a**/**b** and the analogs with separated benzimidazole‐based NHC and pyridine ligands (**BGC0a**/**b**), suggesting that no back coordination of the pyridine ligand occurred, in line with the stability of the neutral complexes in aqueous environment observed by ^1^H NMR.

**BGC** complexes felt in two groups with significant differences in their antiproliferative activity, **BGC12**, **14**, **15**, and **19** being the most toxic candidates and **BGC13**, **16**, **17**, **18**, and **0** being the least toxic ones. Nevertheless, the statistical differences remain modest, showing a very mild impact of the structure of the complexes on their antiproliferative activities.

Considering the equivalent activities for neutral and cationic analogs, only cationic representatives of the **BGC12**, **BGC18**, and **BGC19** couples were further screened on two other human cancer cell lines including hepatic carcinoma Huh‐7 and “triple negative” breast adenocarcinoma MDA‐MB‐231 and noncancerous epithelial breast cells MCF‐10 A (**Table** **1**).

**Table 1:** Antiproliferative activities of complexes **BGC12b**, **BGC18b**, and **BGC19b** against HeLa, Huh‐7, and MDA‐MB‐231 cancer cells and the noncancerous MCF‐10 a cells after 72 h incubation.

Complexes	EC_50_ ± s. d. (μM)			
	HeLa	Huh‐7	MDA‐MB‐231	MCF‐10 A
**BGC12b**	1.8 ± 0.1	1.8 ± 0.1	2.9 ± 0.1	1.2 ± 0.3
**BGC18b**	2.8 ± 0.7	2.5 ± 0.4	4.4 ± 0.8	2.8 ± 0.5
**BGC19b**	1.8 ± 0.6	1.4 ± 0.1	2.0 ± 0.1	1.6 ± 0.4

The three complexes appeared active with EC_50S_ in the low micromolar range against the three tested cancer cell lines. On the three tested cancer cell lines, the same influence of the nature of the NHC ligand was observed with the methylimidazole and pyridoimidazole‐based complexes **BGC12b** and **BGC19b** being more active than the benzimidazole‐based complex **BGC18b**. However, no selectivity for cancer cells could be observed as shown by the similar EC_50_ values against the noncancerous cells line when compared with those against the three cancer cell lines.

### 2.6 Reactivity toward Biomolecules

**Figure 8:**
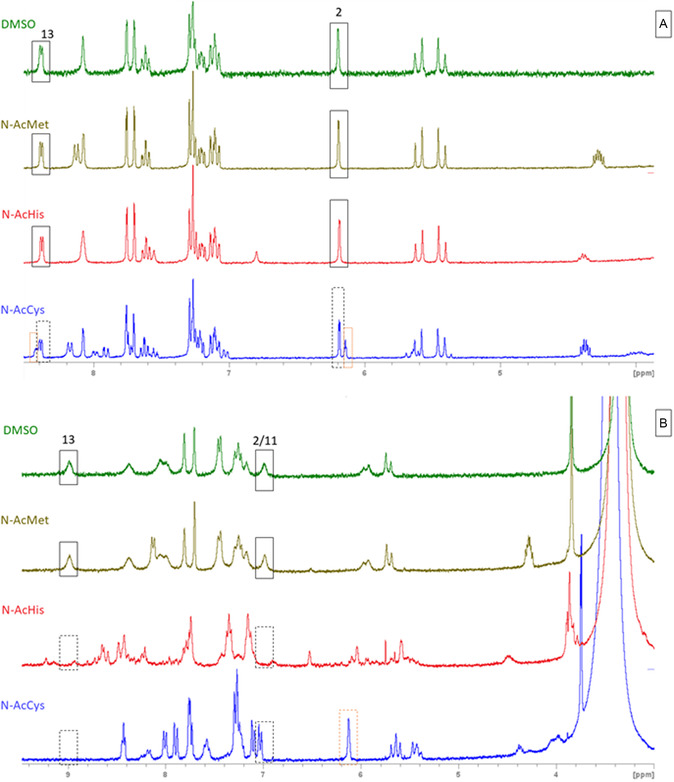
^1^H NMR spectra recorded at 5 mM in DMSO‐d_6_ immediately after mixing of A) **BGC12a** alone and reacted with 1 eq. of N‐Ac‐methionine, N‐Ac‐histidine, and N‐Ac‐cysteine and B) **BGC12b** alone and reacted with 1 eq. of N‐Ac‐methionine, N‐Ac‐histidine, and N‐Ac‐cysteine.

In order to better understand the mode of action of these new **BGC** complexes, all of them were reacted with 1 eq. of N‐acetylated amino acids presenting coordinating functions, namely methionine (thioether), histidine (imidazole), and cysteine (thiol/thiolate) in DMSO‐d_6_, and the ^1^H NMR were immediately recorded after mixing (see experimental part for details). The comparison of the spectra of the pure complexes and the mixtures with the N‐Ac amino acids is given in **Figure** **8** (**BGC12a/b**) and S42–S49, Supporting Information (**BGC13‐19a/b** and **BGC0a/b**), and corresponding NMR conversion rates are given in Table S4, Supporting Information. In the case of neutral complexes, no spectral changes were observed in the presence of N‐Ac‐methionine and N‐Ac‐histidine even after 72 h incubation at 37 °C, suggesting that no reaction has occurred in these conditions. When reacted with N‐Ac‐cysteine, the immediate appearance of a new set of signals in the characteristic regions corresponding to H^2^ and H^13^ was noticed (orange dot squares in Figure 8A and S41–S47A, Supporting Information) that did not evolve over 72 h incubation at 37 °C. Such a behavior is indicative of partial conversion (c.a. 30%) of complexes **BGC12‐19a** similarly to what was observed for **BGC0a** lacking the pyridine side‐chain (Figure S49A, Supporting Information) and **BGC4.** ^[^ ^43^ ^]^

Interestingly, while no spectral changes were observed when the cationic complexes **BGC12‐19b** were reacted with N‐Ac‐methionine, an instantaneous and quantitative conversion took place with both N‐Ac‐histidine and N‐Ac‐cysteine as revealed by the disappearance of the upfield signal of H^13^ (Figure 8B and S42–S48B, Supporting Information) indicative of the opening of the (C^N) chelate. Samples of **BGC12b** reacted with N‐Ac‐cysteine or histidine were further analyzed by HRMS to assess the structures of the reaction products. While the **BGC12b**‐cysteine 1:1 adduct ion could be clearly identified (Figure S50A, Supporting Information, [M + Na]^+^), only the starting **BGC12b** complex was detected for the histidine mix (Figure S50B, Supporting Information), highlighting once again the weakness of the Au—N bond. Unexpectedly, the quinoline‐bearing **BGC16b** turned out less reactive toward N‐Ac‐histidine in comparison to its pyridine counterpart **BGC12b**. Altogether, the chelated cationic complexes **BGC12**‐**19b** appeared more reactive toward amino acids than their neutral counterparts **BGC12**‐**19a**. Moreover, when reacted with N‐Ac cysteine and N‐Ac histidine, signals corresponding to the cationic nonchelated complex **BGC0b** were still present (Figure S49B, Supporting Information), while full conversion was reached for the chelated complex **BGC18b.** Chelation thus seemed to decrease the stability of the Au‐N bond likely due to metallacycle tension, in stark contrast to previously reported half‐sandwich Ir(III) complexes presenting a Cp*‐2‐picolyl chelate.^[^ ^28^ ^]^

### 2.7 Mechanistic Studies

**Figure 9:**
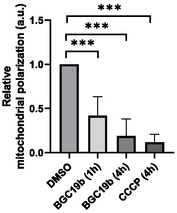
Normalized mitochondrial polarization of HeLa cells treated with DMSO (4 h, normalization reference), **BGC19b** (10 μM, 4 h and 1 h), or **CCCP** (10 μM, 4 h).

Several gold complexes were previously reported to accumulate in mitochondria^[^ ^50^, ^51^ ^]^ or even to disrupt their membrane potential.^[^ ^27^, ^52^, ^53^ ^]^ We therefore investigated the possible antimitochondrial activity of **BGC19b**, chosen as a representative of the most toxic compounds. Briefly, we incubated HeLa cells with **BGC19b** (1 h or 4 h) or **CCCP** (4 h) as positive control and monitored the mitochondrial membrane potential through a JC‐10 endpoint assay. As shown in **Figure** **9**, **BGC19b** induces a significant mitochondrial membrane depolarization as soon as 1 h after addition, while inducing no significant change in cell viability (Figure S51, Supporting Information).

**Figure 10:**
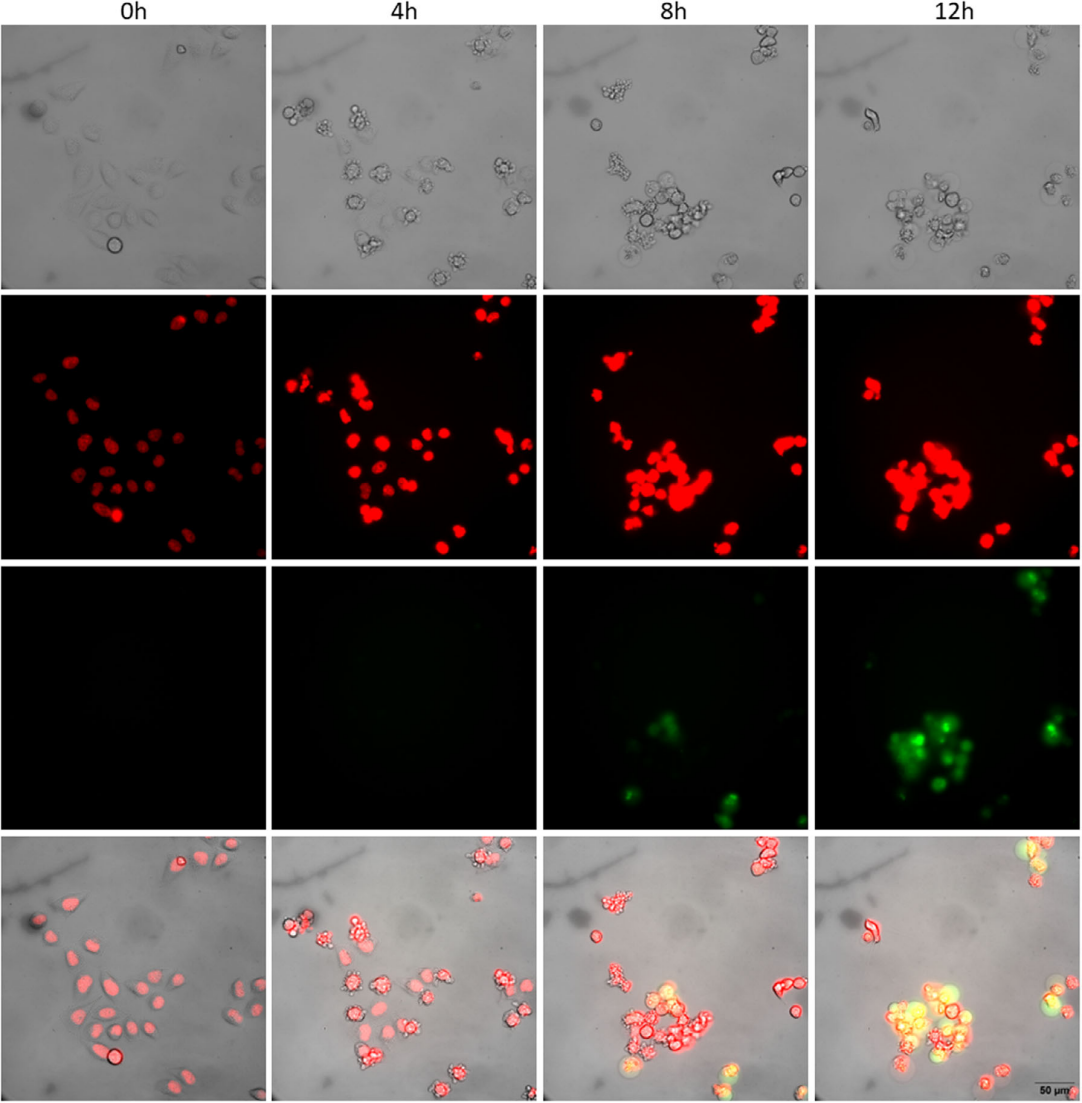
HeLa cells treated with 1.7 μM **BGC19b.** Grayscale: phase contrast; red channel: DNA marker; green channel: caspase 3/7 fluorogenic substrate.

**Figure 11:**
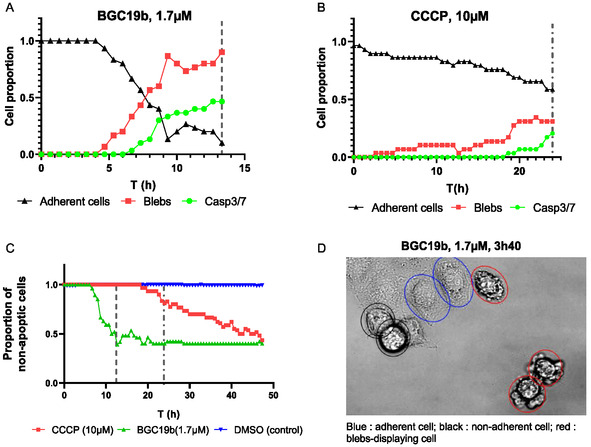
A) Cellular morphology and adhesion over time (0 to 13.3 h) for cells treated with **BGC19b** (1.7 μM). B) Cellular morphology and adhesion over time (0 to 24 h) of cells treated with **CCCP** (10 μM). C) Proportion of nonapoptotic HeLa cells treated with **BGC19b** (1.7 μM), **CCCP** (10 μM), or DMSO (control, 0.1 vol%) over time deduced from (A) and (B). Dashed lines indicate the end time for cell morphology monitoring. D) Example of manual cell tagging for **BGC19b**‐treated cells (1.7 μM) after 3.7 h. Blue: viable cell, black: nonadherent cell, red: bleb‐displaying cell.

Apoptosis is a possible outcome of energy shortage in cells following mitochondrial depolarization. We thus monitored the activity of the effector caspases 3 and 7, as a readout of cell apoptosis. The overall cellular morphology was analyzed in parallel. HeLa cells were incubated with **BGC19b** at its EC_50_ concentration (1.7 μM) along with a DNA deep red stain and caspase 3/7 activation fluorogenic probe, and cells were imaged by time lapse phase contrast and fluorescence videomicroscopy (**Figure** **10**). After an induction period of around 4 h, we observed adhesion loss and membrane blebs followed by caspase 3 and/or 7 activation, which altogether indicates that cells are indeed entering apoptosis (**Figure** **11**A).

For comparison, the same experiment was performed with the mitochondrial uncoupling agent **CCCP** at its EC_50_ (10 μM).^[^ ^54^ ^]^ Apoptosis was detected as expected following adhesion loss and the appearance of blebs but occurred at a much slower rate compared to **BGC19b** (Figure 11B).

The direct comparison of the caspase activation kinetics indicates that **BGC19b** induces apoptosis much faster than **CCCP,** suggesting a different mode of action for the two molecules (Figure 11C).

## 3 Conclusion

We have synthesized a library of eight pairs of biphenyl gold(III) N‐heterocyclic carbene complexes (**BGC** family) presenting NHC‐CH_2_‐heterocycle ligands comprising open neutral complexes of general formula [(C^C)Au(NHC^het)Cl] and chelated cationic complexes of general formula [(C^C)Au(NHC^het)]PF_6_. The complexes were fully characterized and the solid‐state structure of **BGC12a**, **BGC16a**, **BGC18a**, **BGC13b**, **BGC16b**, and **BGC18b** was established by X‐ray diffraction. ^1^H NMR spectroscopy studies revealed the excellent stability of both the cationic and neutral complexes in pure DMSO‐d_6_ and in DMSO‐d_6_/D_2_O mixture over 24 h. However, in all cases, immediate and quantitative conversion of the chelated cationic forms to the open neutral ones occurred in the presence of 1 equiv. chloride ion, suggesting opening of the metallacycle in the chloride‐rich culture medium. Indeed, ^1^H NMR studies of two couples of complexes **BGC12a/b** and **BGC19a/b** in DMSO‐d_6_/serum‐free DMEM 3/1 revealed only the presence of the open neutral forms in both cases. This indicates that, irrespective of the form initially introduced in the culture medium, only the open neutral form is present in the culture medium to interact with the cells. A similar behavior was found for the nonchelated complex **BGC0** indicating that the Au‐N bond was not strengthened by the chelating effect.

All complexes demonstrated high cytotoxic activity on human cancer and noncancer cells with EC_50_ values in the low micromolar range (1.8–3.2 μM) with a limited impact of the nature of the NHC‐heterocycle ligand. Moreover, regardless of the NHC‐heterocycle ligands considered, no difference of activity was observed between the corresponding open neutral and chelated cationic forms. These results are fully consistent with the speciation studies by ^1^H NMR and show that the cytotoxic activity is exclusively due to the neutral form regardless of the introduced form. Reactivity studies with amino acids highlighted thiols as potential interaction sites of these complexes with biomolecules similarly to what was previously observed for related [(C^C)Au(NHC‐R)Cl] complexes.^[^ ^43^ ^]^

Disruption of mitochondrial membrane potential in **BGC19b**‐treated HeLa cells was demonstrated, suggesting that **BGC19b**‐induced cell death might partially result from mitochondrial damages, similarly to other biphenyl Au(III) complexes.^[^ ^27^, ^42^ ^]^ Consistently, time lapse videomicroscopy studies on HeLa cells treated with complex **BGC19b** revealed the time‐dependent activation of the effector caspases 3/7, a signaling event characteristic of apoptotic death pathway. Altogether, these data represent the first study of hemilability in Au(III) complexes for anticancer applications and pave the way for the development of organogold(III) complexes with new reactivities.

## 4 Experimental Section

4.1

4.1.1

##### General Remarks

Chemicals were purchased from various manufacturers and used as received. Unless stated otherwise, all ^1^H and ^13^C NMR spectra were acquired on either one of the following 300 or 400 MHz spectrometers: **Bruker Avance III Nanobay** **400** spectrometer (9.4 T) operating at a ^1^H Larmor frequency of 400.13 MHz with a 5‐mm observe broadband probe (BBFO) z‐axis (^15^N‐^31^P/^19^F/^1^H) at 27 °C, and **Bruker Avance III Nanobay** **300** spectrometer (7 T) operating at a ^1^H Larmor frequency of 300.16 MHz with a 5‐mm observe broadband probe (BBFO) z‐axis (^15^N‐^31^P/^19^F/^1^H) at 27 °C. The spectra were further analyzed using TopSpin 4.4.0 (Bruker). Chemical shifts (*δ*) are expressed as ppm referenced to the solvent residual signal. Splitting patterns are expressed as follows: s, singlet; d, doublet; t, triplet; m, multiplet. Mass spectrometry was carried out at the Mass Spectrometry Sciences Sorbonne University (MS^3^U) platform of Sorbonne Université (Paris). Elemental analyses were performed at the Service Chromato‐Masse Microanalyse of the Université Paris‐Saclay (Châtenay‐Malabry, France) and at the Pôle Chimie Moléculaire‐PACSMUB de l’Université de Bourgogne (Dijon, France). Videomicroscopy and imaging were performed at the Cytométrie et Imagerie Saint‐Antoine platform (CISA, Sorbonne Université, Paris). Gold dimer **[(** ^**t**^ **Bu**_**2**_ **biPh)AuCl]**_**2**_ was synthesized from 4,4′‐ditertbutyl‐1,1′‐bisphenyl through bromination, lithiation, and transmetallation of dibutyltin dichloride following a previously described procedure.^[^ ^47^ ^]^ (2‐Picolyl)azolium salts (imidazolium, benzimidazolium, and pyridoimidazolium salts) **HL1‐8** were synthesized from the corresponding azoles and variously substituted 2‐picolylhalides (bromide or chloride) and analogs according to a reported procedure.^[^ ^44^ ^]^ Complexes **BGC0a/b** were synthesized according to reported procedures.^[^ ^45^ ^]^

##### Synthesis of Neutral Complexes BGC12a‐BGC19a

In a round‐bottom flask, **N‐(2‐methylpyridiyl)‐N′‐methylimidazolium halide derivative**, **N‐(2‐methylpyridiyl)‐N′‐methylbenzimidazolium bromide**, or **N‐(2‐methylpyridiyl)‐pyridoimidazolium** **bromide** (0.2 mmol) was added along with 40 mL of DCM/EtOH mixture (1:1). **Ag**_**2**_ **O** (25.5 mg, 0.11 mmol) was added to the flask prior to sealing it with a glass stopper. The reaction was kept at room temperature in the dark for 4 h under stirring. **[(** ^**t**^ **Bu**_**2**_ **biPh)AuCl]**_**2**_ (100 mg, 0.1 mmol) was added, and the reaction was kept in the dark for another 4 h under stirring. The solution was filtered through celite and the solvents were evaporated under reduced pressure. The resulting oily liquid was dissolved back in a minimal volume of DCM, and a large excess of petroleum ether was added, leading to the formation of a white precipitate. The solvents were evaporated, leaving the crude product as an off white‐yellow powder. The raw product was further purified by silica gel column chromatography using a 5:5 to 6:4 v/v ethyl acetate/petroleum ether mixture to reach R_f_ = 0.3 for the desired product. The solvents were evaporated under reduced pressure, and the remaining solid was further dried under vacuum.

**BGC12a**: AcOEt/Pet. Et. 6:4 eluent. Final yield 122 mg, 0.18 mmol, 89% yield (white solid). ^1^H NMR (300 MHz, CD_3_CN, 300 K): *δ* 8.40 (d, 1 H, ^*3*^ *J*_*H‐H*_ = 5 Hz, H^13^), 8.16 (d, 1 H, ^*4*^ *J*_*H‐H*_ = 2 Hz, H^11^), 7.53 (td, 1 H, ^*3*^ *J*_*H‐H*_ = 8 Hz, ^*4*^ *J*_*H‐H*_ = 2 Hz, H^15^), 7.45 (d, 1 H, ^*3*^ *J*_*H‐H*_ = 2 Hz, H^20^), 7.33 (d, 1 H, ^*3*^ *J*_*H‐H*_ = 2 Hz, H^21^), 7.28 (m, 3 H, H^16^ + H^5^ + H^8^), 7.19 (m, 3 H, H^14^ + H^4^ + H^9^), 6.29 (d, 1 H, ^*4*^ *J*_*H‐H*_ = 2 Hz, H^2^), 5.60 (d, 1 H, ^2^ *J*_*H‐H*_ = 15 Hz, H^18^), 5.38 (d, 1 H, ^2^ *J*_*H‐H*_ = 15 Hz, H^18^), 3.77 (s, 3 H, H^22^), 1.32 (s, 9 H, H_tBu_), 1.09 (s, 9 H, H_tBu_). ^13^C{^1^H} Jmod NMR (75 MHz, CD_3_CN, 300 K): *δ* 184.8 (s, C^19^), 160.8 (s, C^6/7^), 156.0 (s, C^17^), 152.0 (s, C^1/12^), 151.6 (s, C^6/7^), 151.4 (s, C^1/12^), 150.7 (s, C^3/10^), 150.5 (s, C^13^), 149.9 (s, C^3/10^), 137.9 (s, C^15^), 131.1 (s, C^2/11^), 130.84 (s, C^2/11^), 125.1 (s, C^4/9^), 124.9 (s, C^4/9^), 124.5 (s, C^21^), 124.2 (s, C^20^), 124.1 (s, C^14^), 123.8 (s, C^16^), 122.0 (s, C^5/8^), 121.0 (s, C^5/8^), 56.1 (s, C^18^), 38.2 (s, C^22^), 35.8 (s, Ar‐C‐(CH_3_)_3_), 35.0 (s, Ar‐C‐(CH_3_)_3_), 31.8 (s, C_CH3, tBu_), 31.4 (s, C_CH3, tBu_). HRMS (ESI) m/z: [*M*‐Cl]^+^ Calcd for C_30_H_35_N_3_Au^+^ 634.2491. Found 634.2483; (Error: −1.3 ppm). Calcd for C_30_H_35_N_3_AuCl.0.25C_5_H_12_: C, 54.55; H, 5.57; N, 6.11 found: C, 54.79; H, 5.87; N, 5.66.

**BGC13a**: AcOEt/Pet. Et. 5:5 eluent. Final yield 91 mg, 0.13 mmol, 67 % (white solid). ^1^H NMR (300 MHz, CD_3_CN, 300 K): *δ* 8.17 (d, ^4^ *J*_*H‐H*_ = 2 Hz, 1 H, H^11^), 7.49 (d, ^3^ *J*_*H‐H*_ = 2 Hz, 1 H, H^21^), 7.43 (t, ^3^ *J*_*H‐H*_ = 8 Hz, 1 H, H^16^), 7.33 (d, ^3^ *J*_*H‐H*_ = 2 Hz, 1 H, H^22^), 7.30 (d, ^*3*^ *J*_*H‐H*_ = 8 Hz, 2 H, H^5^ + H^8^), 7.19 (dd, ^*4*^ *J*_*H‐H*_ = 2 Hz, ^*3*^ *J*_*H‐H*_ = 8 Hz, 1 H, H^4/9^), 7.13 (dd, ^*4*^ *J*_*H‐H*_ = 2 Hz, ^*3*^ *J*_*H‐H*_ = 8 Hz, 1 H, H^4/9^), 7.07 (d, ^*3*^ *J*_*H‐H*_ = 8 Hz, 1 H, H^15^), 6.99 (d, ^3^ *J*_*H‐H*_ = 8 Hz, 1 H, H^17^), 6.19 (d, ^3^ *J*_*H‐H*_ = 2 Hz, 1 H, H^2^), 5.52 (d, ^2^ *J*_*H‐H*_ = 15 Hz, 1 H, H^19^), 5.31 (d, ^2^ *J*_*H‐H*_ = 15 Hz, 1 H, H^19^), 3.78 (s, 3 H, H^23^), 2.26 (s, 3 H, H^14^), 1.33 (s, 9 H, (CH_3_)_3_^*t*Bu^), 1.05 (s, 9 H, (CH_3_)_3_^*t*Bu^). ^13^C{^1^H} Jmod NMR (75 MHz, CD_3_CN, 300 K): *δ* 184.6 (s, C^20^), 160.7 (s, C^1/12^), 159.5 (s, C^13^), 155.2 (s, C^18^), 152.1 (s, C^6/7^), 151.7 (s, C^1/12^), 151.4 (s, C^6/7^), 150.7 (s, C^3/10^), 149.9 (s, C^3/10^), 138.1 (s, C^16^), 130.8 (s, C^2^  + C^11^), 125.0 (s, C^4/9^), 124.8 (s, C^4/9^), 124.4 (s, C^22^), 123.9 (s, C^21^), 123.6 (s, C^17^), 121.9 (s, C^5/8^), 120.9 (s, C^5/8^), 120.7 (s, C^15^), 56.4 (s, C^19^), 38.1 (s, C^23^), 35.7 (s, C(CH_3_)_3_^*t*Bu^), 34.9 (s, C(CH_3_)_3_^*t*Bu^), 31.7 (s, (CH_3_)_3_^*t*Bu^), 31.4 (s, (CH_3_)_3_^*t*Bu^), 24.2 (s, C^14^). HRMS (ESI) m/z: [*M*‐Cl]^+^ Calcd for C_31_H_37_N_3_Au^+^ 648.2648. Found 648.2644; (Error:−0.6 ppm). Calcd for C_31_H_37_N_3_AuCl.C_6_H_14_: C, 57.70; H, 5.76; N, 5.46 found: C, 57.98; H, 6.76; N, 4.91.

**BGC14a**: AcOEt/Pet. Et. 6:4 eluent. Final yield 108 mg, 0.16 mmol, 80% yield (white solid). ^1^H NMR (300 MHz, CD_3_CN, 300 K): *δ* 8.21 (d, ^3^ *J*_*H‐H*_ = 5 Hz, 1 H, H^13^), 8.16 (d, ^4^ *J*_*H‐H*_ = 2 Hz, 1 H, H^11^), 7.46 (d, ^3^ *J*_*H‐H*_ = 2 Hz, 1 H, H^21^), 7.32 (d, ^3^ *J*_*H‐H*_ = 2 Hz, 1 H, H^22^), 7.30 (d, ^*3*^ *J*_*H‐H*_ = 8 Hz, 1 H, H^5/8^), 7.28 (d, ^*3*^ *J*_*H‐H*_ = 8 Hz, 1 H, H^5/8^), 7.19 (dd, ^4^ *J*_*H‐H*_ = 2 Hz, ^3^ *J*_*H‐H*_ = 8 Hz, 1 H, H^4/9^), 7.18 (d, ^4^ *J*_*H‐H*_ = 2 Hz, 1 H, H^17^), 7.13 (dd, ^4^ *J*_*H‐H*_ = 2 Hz, ^3^ *J*_*H‐H*_ = 8 Hz, 1 H, H^4/9^), 6.90 (*broad* d, ^3^ *J*_*H‐H*_ = 5 Hz, 1 H, H^14^), 6.18 (d, ^4^ *J*_*H‐H*_ = 2 Hz, 1 H, H^2^), 5.52 (d, ^2^ *J*_*H‐H*_ = 15 Hz, 1 H, H^19^), 5.28 (d, ^2^ *J*_*H‐H*_ = 15 Hz, 1 H, H^19^), 3.77 (s, 3 H, H^23^), 1.99 (s, 3 H, H^16^), 1.33 (s, 9 H, (CH_3_)_3_^*t*Bu^), 1.06 (s, 9 H, (CH_3_)_3_^*t*Bu^). ^13^C NMR (75 MHz, CD_3_CN, 300 K): *δ* 184.6 (s, C^20^), 160.7 (s, C^6/7^), 155.5 (s, C^18^), 152.0 (s, C^1/12^), 151.5 (s, C^6/7^), 151.4 (s, C^1/12^), 150.6 (s, C^3/10^), 150.3 (s, C^13^), 149.9 (s, C^3/10^), 149.3 (s, C^15^), 131.0 (s, C^2/11^), 130.8 (s, C^2/11^), 125.4 (s, C^17^), 125.0 (s, C^4/9^ + C^14^), 124.8 (s, ^4/9^C), 124.2 (s, C^21/22^), 124.2 (s, C^21/22^), 121.8 (s, C^5/8^), 121.0 (s, C^5/8^), 56.2 (s, C^19^), 38.2 (s, C^23^), 35.7 (s, C(CH_3_)_3_^*t*Bu^), 34.9 (s, C(CH_3_)_3_^*t*Bu^), 31.8 (s, (CH_3_)_3_^*t*Bu^), 31.4 (s, (CH_3_)_3_^*t*Bu^), 19.8 (s, C^16^). HRMS (ESI) m/z: [*M*‐Cl]^+^ Calcd for C_31_H_37_N_3_Au^+^ 648.2648. Found: 648.2651; (Error: 0.5 ppm). Calcd for C_31_H_37_N_3_AuCl: C, 54.43; H, 5.45; N, 6.14 found: C, 54.26; H, 5.80; N, 5.37.

**BGC15a**: AcOEt/Pet. Et. 6:4 eluent. Final yield 100 mg, 0.14 mmol, 71% (white solid). ^1^H NMR (300 MHz, CD_3_CN, 300 K): *δ* 8.18 (d, ^*3*^ *J*_*H‐H*_ = 6 Hz, 1 H, H^13^), 8.16 (d, ^*4*^ *J*_*H‐H*_ = 2 Hz, 1 H, H^11^), 7.48 (d, ^*3*^ *J*_*H‐H*_ = 2 Hz, 1 H, H^21^), 7.32 (d, ^*3*^ *J*_*H‐H*_ = 2 Hz, 1 H, H^22^), 7.293 (d, ^*3*^ *J*_*H‐H*_ = 8 Hz, 1 H, H^5/8^), 7.288 (d, ^*3*^ *J*_*H‐H*_ = 8 Hz, 1 H, H^5/8^), 7.19 (dd, ^*4*^ *J*_*H‐H*_ = 2, ^*3*^ *J*_*H‐H*_ = 8 Hz, 1 H, H^4/9^), 7.14 (dd, ^*4*^ *J*_*H‐H*_ = 2, ^*3*^ *J*_*H‐H*_ = 8 Hz, 1 H, H^4/9^), 6.90 (d, ^*4*^ *J*_*H‐H*_ = 3 Hz, 1 H, H^17^), 6.63 (dd, ^*4*^ *J*_*H‐H*_ = 3, ^*3*^ *J*_*H‐H*_ = 6 Hz, 1 H, H^14^), 6.21 (d, ^*4*^ *J*_*H‐H*_ = 2 Hz, 1 H, H^2^), 5.50 (d, ^*2*^ *J*_*H‐H*_ = 15 Hz, 1 H, H^19^), 5.29 (d, ^*2*^ *J*_*H‐H*_ = 15 Hz, 1 H, H^19^), 3.77 (s, 3 H, H^23^), 3.53 (s, 3 H, H^16^), 1.33 (s, 9 H, (CH_3_)_3_^*t*Bu^), 1.07 (s, 9 H, (CH_3_)_3_^*t*Bu^). ^13^C NMR (75 MHz, CD_3_CN, 300 K) *δ* 184.6 (s, C^20^), 167.1 (s, C^15^), 160.7 (s, C^6/7^), 157.5 (s, C^18^), 152.0 (s, C^1/12^), 151.7 (s, C^13^), 151.4 (s, C^6/7^+C^1/12^), 150.7 (s, C^3/10^), 149.9 (s, C^3/10^), 131.0 (s, C^2/11^), 130.8 (s, C^2/11^), 125.1 (s, C^4/9^), 124.8 (s, C^4/9^), 124.3 (s, C^22^), 124.2 (s, C^21^), 121.9 (s, C^5/8^), 121.0 (s, C^5/8^), 110.5 (s, C^14^), 110.4 (s, C^17^), 56.3 (s, C^19^), 55.8 (s, C^16^), 38.1 (s, C^23^), 35.7 (s, C(CH_3_)_3_^*t*Bu^), 34.9 (s, C(CH_3_)_3_^*t*Bu^), 31.7 (s, (CH_3_)_3_^*t*Bu^), 31.3 (s, (CH_3_)_3_^*t*Bu^). HRMS (ESI) m/z: [*M*‐Cl]^+^ Calcd for C_31_H_37_ON_3_Au^+^ 664.2597. Found 664.2599; (Error: 0.3 ppm). Calcd for C_31_H_37_ON_3_AuCl.0.33C_5_H_12_: C, 55.12; H, 6.06; N, 5.62 found: C, 55.35; H, 6.05; N, 5.01.

**BGC16a**: AcOEt/Pet. Et. 5:5 eluent. Final yield 64 mg, 0.09 mmol, 45% yield (white solid). ^1^H NMR (CD_3_CN, 300 K): *δ* 8.17 (d, 1 H, ^*4*^ *J*_*H‐H*_ = 2 Hz, H^11^), 8.07 (d, 1 H, ^*3*^ *J*_*H‐H*_ = 9 Hz, H^20^), 7.77(d, 1 H, ^*3*^ *J*_*H‐H*_ = 8 Hz, H^14/17^), 7.74 (d, 1 H, ^*3*^ *J*_*H‐H*_ = 8 Hz, H^14/17^), 7.61 (td, 1 H, ^*3*^ *J*_*H‐H*_ = 8 Hz, ^*4*^ *J*_*H‐H*_ = 1 Hz, H^15/16^), 7.54 (d, 1 H, ^*3*^ *J*_*H‐H*_ = 2 Hz, H^24/25^), 7.49 (dt, 1 H, ^*3*^ *J*_*H‐H*_ = 8 Hz, ^*4*^ *J*_*H‐H*_ = 1 Hz, H^15/16^), 7.41 (d, 1 H, ^*3*^ *J*_*H‐H*_ = 9 Hz, H^19^), 7.36 (d, 1 H, ^*3*^ *J*_*H‐H*_ = 2 Hz, H^24/25^), 7.27 (d, 1 H, ^*3*^ *J*_*H‐H*_ = 8 Hz, H^5/8^), 7.23 (d, 1 H, ^*3*^ *J*_*H‐H*_ = 8 Hz, H^5/8^), 7.18 (dd, ^*3*^ *J*_*H‐H*_ = 8 Hz, ^*4*^ *J*_*H‐H*_ = 2 Hz, H^4/9^), 7.06 (dd, ^*3*^ *J*_*H‐H*_ = 8 Hz, ^*4*^ *J*_*H‐H*_ = 2 Hz, H^4/9^), 6.22 (d, ^*3*^ *J*_*H‐H*_ = 2 Hz, H^2^), 5.76 (d, 1 H, ^*2*^ *J*_*H‐H*_ = 15 Hz, H^22^), 5.56 (d, 1 H, ^*2*^ *J*_*H‐H*_ = 15 Hz, H^22^), 3.79 (s, 3 H, H^26^), 1.33 (s, 9 H, H^*tBu*^), 0.96 (s, 9 H, H^*tBu*^). ^13^C{^1^H} Jmod NMR (75 MHz, CD_3_CN, 300 K): *δ* 185.0 (s, C^23^), 160.8 (s, C^6/7^), 156.3 (s, C^21^), 152.0 (s, C^1/12^), 151.8 (s, C^6/7^), 151.5 (s, C^1/12^), 150.6 (s, C^3/10^), 149.9 (s, C^3/10^), 148.4 (s, C^18^), 138.2 (s, C^19/20^), 130.8 (s, C^2/11^), 130.8 (s, C^2/11^), 130.7 (s, C^15/16^), 130.0 (s, C^14/17^), 128.7 (s, C^14/17^), 128.4 (s, C^13^), 127.7 (s, C^15/16^), 125.0 (s, C^4/9^), 124.8 (s, C^4/9^), 124.6 (s, C^24/25^), 124.2 (s, C^24/25^), 122.0 (s, C^5/8^), 121.3 (s, C^19/20^), 121.0 (s, C^5/8^), 56.8 (s, C^22^), 35.8 (s, C^IV,tBu^), 34.9 (s, C^IV,tBu^), 31.8 (s, C^CH3,tBu^), 31.3 (s, C^CH3,tBu^). HRMS (ESI) m/z: [*M*‐Cl]^+^ Calcd for C_34_H_37_N_3_Au^+^ 684.2648. Found 684.2645; (Error:−0.4 ppm). Calcd for C_34_H_37_N_3_AuCl.0.25C_5_H_12_: C, 57.36; H, 5.46; N, 5.69 found: C, 57.43; H, 5.54; N, 5.29.

**BGC17a**: AcOEt/Pet. Et. 5:5 eluent. Final yield 99 mg, 0.15 mmol, 73% yield (white solid). ^1^H NMR (300 MHz, CD_3_CN, 300 K): *δ* 8.55 (s, 1 H, H^15^), 8.35 (m, 2 H, H^13^ + H^14^), 8.15 (d, ^4^ *J*_*H‐H*_ = 2 Hz, 1 H, H^11^), 7.50 (d, ^3^ *J*_*H‐H*_ = 2 Hz, 1 H, H^19^), 7.36 (d, ^3^ *J*_*H‐H*_ = 2 Hz, 1 H, H^20^), 7.29 (d, ^3^ *J*_*H‐H*_ = 8 Hz, 1 H, H^5/8^), 7.28 (d, ^3^ *J*_*H‐H*_ = 8 Hz, 1 H, H^5/8^), 7.17 (dd, ^3^ *J*_*H‐H*_ = 8 Hz, ^4^ *J*_*H‐H*_ = 2 Hz, 1 H, H^4/9^), 7.14 (dd, ^3^ *J*_*H‐H*_ = 8 Hz, ^4^ *J*_*H‐H*_ = 2 Hz, 1 H, H^4/9^), 6.18 (d, ^4^ *J*_*H‐H*_ = 2 Hz, 1 H, H^2^), 5.68 (d, ^2^ *J*_*H‐H*_ = 15 Hz, 1 H, H^17^), 5.42 (d, ^2^ *J*_*H‐H*_ = 15 Hz, 1 H, H^17^), 3.77 (s, 3 H, H^21^), 1.33 (s, 9 H, (CH_3_)_3_^*t*Bu^), 1.09 (s, 9 H, (CH_3_)_3_^*t*Bu^). ^13^C NMR (75 MHz, CD_3_CN, 300 K): *δ* 185.1 (s, C^18^), 160.62 (s, C^6/7^), 152.0 (s, C^1/12^), 151.7 (s, C^16^), 151.5 (s, C^6/7^), 151.6 (s, C^1/12^), 150.7 (s, C^3/10^), 149.9 (s, C^3/10^), 145.3 (s, 2C, C^14^ + C^13^), 145.2 (s, C^15^), 130.9 (s, C^2/11^), 130.8 (s, C^2/11^), 125.1 (s, C^4/9^), 124.9 (s, C^4/9^), 124.6 (s, C^20^), 124.4 (s, C^19^), 122.0 (s, C^5/8^), 121.0 (s, C^5/8^), 53.7 (s, C^17^), 38.2 (s, C^21^), 35.7 (s, C(CH_3_)_3_^*t*Bu^), 34.9 (s, C(CH_3_)_3_^*t*Bu^), 31.7 (s, (CH_3_)_3_^*t*Bu^), 31.4 (s, (CH_3_)_3_^*t*Bu^). HRMS (ESI) m/z: [*M*‐Cl]^+^ Calcd for C_29_H_34_N_4_Au^+^ 635.2444. Found 635.2448; (Error: 0.7 ppm). Calcd for C_29_H_34_N_4_AuCl: C, 51.91; H, 5.11; N, 8.35 found: C, 52.09; H, 5.29; N, 7.69.

**BGC18a**: AcOEt/Pet. Et. 3:7 eluent. Final yield 116 mg, 0.16 mmol, 81 % (white solid). ^1^H NMR (300 MHz, CDCl_3_, 300 K): *δ* 8.49 (d, ^3^ *J*_*H‐H*_ = 4 Hz, 1 H, H^13^), 8.34 (d, ^4^ *J*_*H‐H*_ = 2 Hz, 1 H, H^11^), 7.67 (dd, ^4^ *J*_*H‐H*_ = 1 Hz, ^3^ *J*_*H‐H*_ = 7 Hz, 1 H, H^23^), 7.54 (m, 2 H, H^21^+H^24^), 7.50‐7.38 (m, 3 H, H^15^ + H^22^+H^14^), 7.32 (d, ^*3*^ *J*_*H‐H*_ = 8 Hz, 2 H, H^5^+H^8^), 7.23 (dd, ^4^ *J*_*H‐H*_ = 2 Hz, ^3^ *J*_*H‐H*_ = 8 Hz, 1 H, H^4/9^), 7.12 (dd, ^4^ *J*_*H‐H*_ = 2 Hz, ^3^ *J*_*H‐H*_ = 8 Hz, 1 H, H^4/9^), 7.09 (m, 1 H, H^16^), 6.38 (d, ^4^ *J*_*H‐H*_ = 2 Hz, 1 H, H^2^), 5.89 (d, ^2^ *J*_*H‐H*_ = 15 Hz, 1 H, H^18^), 5.87 (d, ^2^ *J*_*H‐H*_ = 15 Hz, 1 H, H^18^), 4.10 (s, 3 H, H^26^), 1.40 (s, 9 H, (CH_3_)_3_^*t*Bu^), 0.98 (s, 9 H, (CH_3_)_3_^*t*Bu^). ^13^C NMR (75 MHz, CDCl_3_, 300 K): *δ* 193.8 (s, C^19^), 159.9 (s, C^6/7^), 154.4 (s, C^17^), 151.6 (s, C^1/12^ + C^6/7^), 150.3 (s, C^1/12^), 149.7 (s, C^3/10^), 149.6 (s, C^3/10^), 149.5 (s, C^13^), 137.3 (s, C^15^), 135.0 (s, C^25^), 134.0 (s, C^20^), 130.2 (s, C^2/11^), 129.8 (s, C^2/11^), 124.9 (s, C^22^ + C^16^), 124.4 (s, C^4/9^), 124.3 (s, C^4/9^), 123.4 (s, C^14/21^), 123.4 (s, C^14/21^), 121.4 (s, C^5/8^), 120.3 (s, C^5/8^), 113.1 (s, C^23^), 111.1 (s, C^24^), 54.3 (s, C^18^), 35.4 (s, C(CH_3_)_3_^*t*Bu^), 34.7 (s, C^26^), 34.4 (s, C(CH_3_)_3_^*t*Bu^), 31.6 (s, (CH_3_)_3_^*t*Bu^), 31.2 (s, (CH_3_)_3_^*t*Bu^). HRMS (ESI) m/z: [*M*‐Cl]^+^ Calcd for C_34_H_37_N_3_Au^+^ 684.2648. Found 684.2646; (Error: −0.2 ppm). Calcd for C_34_H_37_N_3_AuCl.C_5_H_12_: C, 59.13; H, 6.23; N, 5.30 found: C, 59.10; H, 6.16; N, 4.85.

**BGC19a**: AcOEt/Pet. Et. 5:5 eluent. Final yield 113 mg, 0.16 mmol, 80% (white solid). ^1^H NMR (300 MHz, CDCl_3_, 300 K): *δ* 8.41 (d, ^*3*^ *J*_*H‐H*_ = 5 Hz, 1 H, H^13^), 8.28 (s, 1 H, H^11^), 8.22 (d, ^*3*^ *J*_*H‐H*_ = 7 Hz, 1 H, H^22^), 7.62 (s, 1 H, H^20^), 7.45 (d, ^*3*^ *J*_*H‐H*_ = 8 Hz, 1 H, H^15^), 7.41 (dd, ^*3*^ *J*_*H‐H*_ = 7 Hz, ^4^ *J*_*H‐H*_ = 1 Hz, 1 H, H^16^), 7.34 (d, ^*3*^ *J*_*H‐H*_ = 9 Hz, 1 H, H^25^), 7.21 (d, ^*3*^ *J*_*H‐H*_ = 8 Hz, 1 H, H^5/8^), 7.19 (d, ^*3*^ *J*_*H‐H*_ = 8 Hz, 1 H, H^5/8^), 7.13 (dd, ^*3*^ *J*_*H‐H*_ = 8 Hz, ^*4*^ *J*_*H‐H*_ = 2 Hz, 1 H, H^4/9^), 7.03 (ddd, ^*4*^ *J*_*H‐H*_ = 1 Hz, ^*3*^ *J*_*H‐H*_ = 5 Hz, ^*3*^ *J*_*H‐H*_ = 7 Hz, 1 H, H^14^), 6.97 (dd, ^*3*^ *J*_*H‐H*_ = 8 Hz, ^*4*^ *J*_*H‐H*_ = 2 Hz, 1 H, H^4/9^), 6.82 (dd, ^*3*^ *J*_*H‐H*_ = 7 Hz, ^*3*^ *J*_*H‐H*_ = 9 Hz, 1 H, H^24^), 6.53 (t, ^*3*^ *J*_*H‐H*_ = 7 Hz, 1 H, H^23^), 5.89 (d, ^*2*^ *J*_*H‐H*_ = 15 Hz, 1 H, H^18^), 5.84 (s, 1 H, H^2^), 5.60 (d, ^*2*^ *J*_*H‐H*_ = 15 Hz, 1 H, H^18^), 1.31 (s, 9 H, (CH_3_)_3_^*t*Bu^), 0.80 (s, 9 H, (CH_3_)_3_^*t*Bu^). ^13^C NMR (75 MHz, CDCl_3_, 300 K): *δ* 176.0 (s, C^19^), 160.0 (s, C^6/7^), 154.2 (s, C^17^), 151.4 (s, C^6/7^), 151.2 (s, C^1/12^), 150.2 (s, C^1/12^), 149.6 (s, C^13^), 149.6 (s, C^3/10^), 149.2 (s, C^3/10^), 137.3 (s, C^15/16^), 131.3 (s, C^21^), 130.2 (s, C^2/11^), 130.0 (s, C^2/11^), 127.1 (s, C^22^), 124.1 (s, C^15/16^), 124.0 (s, C^4/9^), 123.9 (s, C^4/9^), 123.5 (s, C^14^), 123.3 (s, C^24^), 121.1 (s, C^5/8^), 120.1 (s, C^5/8^), 117.5 (s, C^25^), 114.3 (s, C^23^), 112.7 (s, C^20^), 57.0 (s, C^18^), 35.3 (s, C(CH_3_)_3_^*t*Bu^), 34.2 (s, C(CH_3_)_3_^*t*Bu^), 31.5 (s, (CH_3_)_3_^*t*Bu^), 30.9 (s, (CH_3_)_3_^*t*Bu^). HRMS (ESI) m/z: [*M*‐Cl]^+^ Calcd for C_33_H_35_N_3_Au^+^ 670.2491. Found 670.2496; (Error: 0.7 ppm). Calcd for C_33_H_35_N_3_AuCl: C, 56.14; H, 5.00; N, 5.95 found: C, 56.30; H, 5.64; N, 5.00.

##### Synthesis of Cationic Complexes BGC12b‐19b

In a round bottom flask, **BGC12‐19a** (50 mg, 1 eq.) was dissolved in dichloromethane (5 mL) under stirring. **AgPF**_**6**_ (1.3 eq.) was dissolved in acetonitrile (1 mL) and added to the reaction mixture, leading to the formation of a cloudy white precipitate. The reaction was then stirred for 3 h in the dark at room temperature and then filtered through celite and rinsed with dichloromethane. The solvents were then evaporated and the product was reprecipitated using a minimal amount of DCM followed by the addition of an excess of petroleum ether. The products were obtained as yellow to brownish powders and were further dried under vacuum.

**BGC12b**: Final yield 59 mg, 0.076 mmol, quant. (white solid). ^1^H NMR (300 MHz, CD_3_CN, 300 K): *δ* 8.88 (d, ^*3*^ *J*_*H‐H*_ = 6 Hz, 1 H, H^13^), 8.24 (dt, ^*2*^ *J*_*H‐H*_ = 2 Hz, ^*3*^ *J*_*H‐H*_ = 8 Hz, 1 H, H^15^), 7.93 (d, ^*3*^ *J*_*H‐H*_ = 8 Hz, 1 H, H^16^), 7.85 (ddd, ^*4*^ *J*_*H‐H*_ = 1 Hz, ^*3*^ *J*_*H‐H*_ = 6 Hz, ^*3*^ *J*_*H‐H*_ = 8 Hz, 1 H, H^14^), 7.51 (d, ^*4*^ *J*_*H‐H*_ = 2 Hz, 1 H, H^20/21^), 7.42 (d, ^*3*^ *J*_*H‐H*_ = 8 Hz, 2 H, H^5^+H^8^), 7.34 (d, ^*3*^ *J*_*H‐H*_ = 2 Hz, 1 H, H^20/21^), 7.30 (dd, ^*4*^ *J*_*H‐H*_ = 2 Hz, ^*3*^ *J*_*H‐H*_ = 8 Hz, 1 H, H^4/9^), 7.27 (m, 2 H, H^11^+H^4/9^), 7.03 (d, ^*3*^ *J*_*H‐H*_ = 2 Hz, 1 H, H^2^), 5.77 (d, ^*2*^ *J*_*H‐H*_ = 15 Hz, 1 H, H^18^), 5.42 (d, ^*2*^ *J*_*H‐H*_ = 15 Hz, 1 H, H^18^), 3.85 (s, 3 H, H^22^), 1.29 (s, 9 H, (CH_3_)_3_^*t*Bu^), 1.24 (s, 9 H, (CH_3_)_3_^*t*Bu^). ^13^C{^1^H} Jmod NMR (75 MHz, CD_3_CN, 300 K): *δ* 182.3 (s, C^19^), 161.4 (s, C^6/7^), 154.6 (s, C^17^), 153.4 (s, C^13^), 152.5 (s, C^1/12^), 151.4 (s, C^1/12^), 150.9 (s, C^3/10^), 150.6 (s, C^3/10^), 146.3 (s, C^6/7^), 143.5 (s, C^15^), 133.7 (s, C^2/11^), 130.1 (s, C^2/11^), 127.6 (s, C^14^), 127.4 (s, C^16^), 126.3 (s, C^4/9^), 125.8 (s, C^4/9^), 125.3 (s, C^20/21^), 123.8 (s, C^20/21^), 122.7 (s, C^5/8^), 121.7 (s, C^5/8^), 55.7 (s, C^18^), 39.0 (s, C^22^), 35.6 (s, C(CH_3_)_3_^*t*Bu^), 35.3 (s, C(CH_3_)_3_^*t*Bu^), 31.4 (s, 2*(CH_3_)_3_^*t*Bu^). HRMS (ESI) m/z: [*M*‐PF_6_]^+^ Calcd for C_30_H_35_AuN_3_^+^ 634.2491. Found 634.2477; (Error:−2.2 ppm). Calcd for C_30_H_35_N_3_AuPF_6_.3H_2_O: C, 43.23; H, 4.96; N, 5.04 found: C, 43.30; H, 4.30; N, 4.80.

**BGC13b**: Final yield 59 mg, 0.073 mmol, quant. (yellowish powder). ^1^H NMR (300 MHz, CD_3_CN, 300 K): *δ* 8.12 (t, ^3^ *J*_*H‐H*_ = 7.8 Hz, 1 H, H^16^), 7.76 (d, ^3^ *J*_*H‐H*_ = 8 Hz, 1 H, H^15/17^), 7.74 (d, ^3^ *J*_*H‐H*_ = 8 Hz, 1 H, H^15/17^), 7.49 (d, ^3^ *J*_*H‐H*_ = 2 Hz, 1 H, H^21/22^), 7.44‐7.33 (m, 3 H, H^5^ + H^8^ + H^21/22^), 7.31 (s, 1 H, H^11^), 7.28 (dd, ^4^ *J*_*H‐H*_ = 2 Hz, ^3^ *J*_*H‐H*_ = 8 Hz, 1 H, H^4/9^), 7.21 (dd, ^4^ *J*_*H‐H*_ = 2 Hz, ^3^ *J*_*H‐H*_ = 8 Hz, 1 H, H^4/9^), 6.66 (d, ^4^ *J*_*H‐H*_ = 2 Hz, 1 H, H^2^), 5.77 (d, ^2^ *J*_*H‐H*_ = 16 Hz, 1 H, H^19^), 5.36 (d, ^2^ *J*_*H‐H*_ = 16 Hz, 1 H, H^19^), 3.94 (s, 3 H, H^23^), 2.73 (s, 3 H, H^14^), 1.29 (s, 9 H, (CH_3_)_3_^*t*Bu^), 1.16 (s, 9 H, (CH_3_)_3_^*t*Bu^). ^13^C NMR (75 MHz, CD_3_CN, 300 K): *δ* 182.7 (s, C^20^), 161.9 (s, C^13^), 159.3 (s, C^6/7^), 154.3 (s, C^18^), 152.7 (s, C^1/12^), 151.2 (s, C^1/12^), 151.2 (s, C^3/10^), 150.6 (s, C^3/10^), 147.0 (s, C^6/7^), 142.9 (s, C^16^), 133.5 (s, C^2/11^), 130.8 (s, C^2/11^), 129.1 (s, C^15^), 126.5 (s, C^4/9^), 125.8 (s, C^4/9^), 125.4 (s, C^22^), 124.8 (s, C^17^), 124.1 (s, C^21^), 122.8 (s, C^5/8^), 121.9 (s, C^5/8^), 55.8 (s, C^19^), 38.8 (s, C^23^), (s, 2 C(CH_3_)_3_^*t*Bu^), 31.6 (s, (CH_3_)_3_^*t*Bu^), 31.4 (s, (CH_3_)_3_^*t*Bu^), 27.9 (s, C^14^). HRMS (ESI) m/z: [*M*‐PF_6_] + Calcd for C_31_H_37_N_3_Au^+^ 648.2648. Found 648.2644; (Error: −0.6 ppm). Calcd for C_31_H_37_N_3_AuPF_6_.0,5C_5_H_12_: C, 48.5; H, 5.22; N, 5.06 found : C, 48.83; H, 5.35; N, 5.07

**BGC14b**: Reaction on 40 mg scale. Final yield 33 mg, 0.04 mmol, 70% (off‐white powder). ^1^H NMR (300 MHz, CD_3_CN, 300 K): *δ* 8.70 (d, ^3^ *J*_*H‐H*_ = 6 Hz, 1 H, H^13^), 7.75 (s, 1 H, H^17^), 7.66 (d, ^3^ *J*_*H‐H*_ = 6 Hz, 1 H, H^14^), 7.50 (s, 1 H, H^21^), 7.40 (d, ^3^ *J*_H‐H_ = 8 Hz, 2 H, H^5^ + H^8^), 7.31 (m, 4 H, H^4^ + H^9^ + H^22^ + H^11^), 7.08 (s, 1 H, H^2^), 5.71 (d, ^2^ *J*_*H‐H*_ = 15 Hz, 1 H, H^19^), 5.34 (d, ^2^ *J*_*H‐H*_ = 15 Hz, 1 H, H^19^), 3.85 (s, 3 H, H^23^), 2.55 (s, 3 H, H^16^), 1.29 (s, 9 H, (CH_3_)_3_^*t*Bu^), 1.25 (s, 9 H, (CH_3_)_3_^*t*Bu^). ^13^C NMR (101 MHz, CD_3_CN, 300 K): *δ* 182.4 (s, C^20^), 161.4 (s, C^6/7^), 156.7 (s, C^15^), 153.9 (s, C^18^), 152.7 (s, C^1/12^), 152.6 (s, C^13^), 151.7 (s, C^1/12^), 150.9 (s, C^3/10^), 150.6 (s, C^3/10^), 146.5 (s, C^6/7^), 133.8 (s, C^2/11^), 130.2 (s, C^2/11^), 128.2 (s, C^14/17^), 128.1 (s, C^14/17^), 126.3 (s, C^4/9^), 125.8 (s, C^4/9^), 125.4 (s, C^22^), 123.8 (s, C^21^), 122.7 (s, C^5/8^), 121.8 (s, C^5/8^), 55.8 (s, C^19^), 39.1 (s, C^23^), 35.7 (s, C(CH_3_)_3_^*t*Bu^), 35.3 (s, C(CH_3_)_3_^*t*Bu^), 31.5 (s, (CH_3_)_3_^*t*Bu^), 31.5 (s, (CH_3_)_3_^*t*Bu^), 21.6 (s, C^16^). HRMS (ESI) m/z: [*M*‐PF_6_]^+^ Calcd for C_31_H_37_N_3_Au^+^ 648.2648. Found 648.2668; (Error: 3.1 ppm). Calcd for C_31_H_37_N_3_AuPF_6_.C_5_H_12_: C, 49.95; H, 5.71; N, 4.85 found: C, 49.74; H, 5.33; N, 4.53.

**BGC15b**: Final yield 58 mg, 0.072 mmol, quant. (yellow powder). ^1^H NMR (300 MHz, CD_3_CN, 300 K): *δ* 8.64 (d, ^3^ *J*_*H‐H*_ = 7 Hz, 1 H, H^13^), 7.50 (d, ^3^ *J*_*H‐H*_ = 2 Hz, 1 H, H^21/22^), 7.43 (d, ^4^ *J*_*H‐H*_ = 3 Hz, 1 H, H^17^), 7.39 (d, ^3^ *J*_H‐H_ = 8 Hz, 2 H, H^5^ + H^8^), 7.35 (d, ^3^ *J*_*H‐H*_ = 2 Hz, 1 H, H^21/22^), 7.31 (m, 1 H, H^14^), 7.30‐7.25 (m, 3 H, H^4^ + H^9^ + H^11^), 7.10 (d, ^4^ *J*_*H‐H*_ = 2 Hz, 1 H, H^2^), 5.67 (d, ^2^ *J*_*H‐H*_ = 15 Hz, 1 H, H^19^), 5.30 (d, ^2^ *J*_*H‐H*_ = 15 Hz, 1 H, H^19^), 4.03 (s, 1 H, H^16^), 3.85 (s, 3 H, H^23^), 1.28 (s, 9 H, (CH_3_)_3_^*t*Bu^), 1.26 (s, 9 H, (CH_3_)_3_^*t*Bu^). ^13^C NMR (75 MHz, CD_3_CN, 300 K): *δ* 182.4 (s, C^20^), 170.3 (s, C^15^), 161.2 (s, C^6/7^), 155.8 (s, C^18^), 154.4 (s, C^13^), 152.5 (s, C^1/12^), 151.5 (s, C^1/12^), 150.9 (s, C^3/10^), 150.6 (s, C^3/10^), 146.5 (s, C^6/7^), 133.8 (s, C^2/11^), 130.2 (s, C^2/11^), 126.2 (s, C^4/9^), 125.8 (s, C^4/9^), 125.3 (s, C^21^), 123.7 (s, C^22^), 122.6 (s, C^5/8^), 121.7 (s, C^5/8^), 113.7 (s, C^17^), 112.8 (s, C^14^), 57.8 (s, C^16^), 55.8 (s, C^19^), 39.1 (s, C^23^), 35.6 (s, C(CH_3_)_3_^*t*Bu^), 35.3 (s, C(CH_3_)_3_^*t*Bu^), 31.5 (s, (CH_3_)_3_^*t*Bu^), 31.4 (s, (CH_3_)_3_^*t*Bu^). HRMS (ESI) m/z: [*M*‐PF_6_]^+^ Calcd for C_31_H_37_ON_3_Au^+^ 664.2597. Found 664.2620; (Error: 3.5 ppm). Calcd for C_31_H_37_ON_3_AuPF_6_.0,25C_5_H_12_: C, 46.8; H, 4.87; N, 5.08 found: C, 46.59; H, 5.32; N, 4.95

**BGC16b**: Final yield 66 mg, 0.079 mmol, quant. (yellowish powder). ^1^H NMR (300 MHz, CD_3_CN, 300 K): *δ* 8.82 (d, ^3^ *J*_*H‐H*_ = 8 Hz, 1 H, H^19^), 8.42 (m, 1 H, H^14^), 8.22 (m, 1 H, H^17^), 8.03 (d, ^3^ *J*_*H‐H*_ = 8 Hz, 1 H, H^20^), 7.79 (m, 2 H, H^15^ + H^16^), 7.54 (d, ^3^ *J*_*H‐H*_ = 2 Hz, 1 H, H^24/25^), 7.42 (d, ^3^ *J*_*H‐H*_ = 8 Hz, 1 H, H^5/8^), 7.35 (m, 4 H, H^4/9^+H^5/8^ + H^11^+H^24/25^), 7.12 (dd, ^4^ *J*_*H‐H*_ = 2 Hz, ^3^ *J*_*H‐H*_ = 8 Hz, 1 H, H^4/9^), 6.10 (d, ^4^ *J*_*H‐H*_ = 2 Hz, 1 H, H^2^), 5.96 (d, ^2^ *J*_*H‐H*_ = 16 Hz, 1 H, H^22^), 5.60 (d, ^2^ *J*_*H‐H*_ = 16.0 Hz, 1 H, H^22^), 3.96 (s, 3 H, H^26^), 1.32 (s, 9 H, (CH_3_)_3_^*t*Bu^), 0.75 (s, 9 H, (CH_3_)_3_^*t*Bu^). ^13^C NMR (75 MHz, CD_3_CN, 300 K): *δ* 183.3 (s, C^23^), 159.8 (s, C^6/7^), 157.8 (s, C^21^), 152.8 (s, C^1/12^), 151.3 (s, C^1/12^), 151.2 (s, C^3/10^), 149.9 (s, C^3/10^), 147.5 (s, C^6/7^), 145.8 (s, C^18^), 144.0 (s, C^19/20^), 133.4 (s, C^2/11^), 132.7 (s, 2C, C^2/11^ + C^16^), 131.2 (s, C^13^), 130.0 (s, C^15^), 129.9 (s, 2C, C^14^ + C^17^), 126.5 (s, C^4/9^), 125.5 (s, C^4/9^), 125.3 (s, C^25^), 124.4 (s, C^19/20^), 124.3 (s, C^24^), 122.8 (s, C^5/8^), 121.6 (s, C^5/8^), 56.6 (s, C^22^), 38.9 (s, C^26^), 35.5 (s, 9 H, (CH_3_)_3_^*t*Bu^), 34.9 (s, (CH_3_)_3_^*t*Bu^), 31,6 (s, C(CH_3_)_3_^*t*Bu^), 30,9 (s, C(CH_3_)_3_^*t*Bu^). HRMS (ESI) m/z: [M]+ Calcd for C_34_H_37_N_3_Au 684.2648. Found 684.2643; (Error: −0.7 ppm). Calcd for C_34_H_37_N_3_AuPF_6_.4H_2_O: C, 45.29; H, 5.03; N, 4.66 found: C, 45.48; H, 4.55; N, 4.36

**BGC17b**: Final yield 55 mg, 0.07 mmol, 95 % (orange powder). ^1^H NMR (300 MHz, CD_3_CN, 300 K): *δ* 9.18 (s, 1 H, H^15^), 9.10 (s, 1 H, H^14^), 8.85 (s, 1 H, H^13^), 7.56 (d, ^3^ *J*_*H‐H*_ = 2 Hz, 1 H, H^19^), 7.40 (m, 3 H, H^5^ + H^8^ + H^20^), 7.31 (dd, ^4^ *J*_*H‐H*_ = 2 Hz, ^3^ *J*_*H‐H*_ = 8 Hz, 1 H, H^4/9^), 7.28 (dd, ^3^ *J*_*H‐H*_ = 2 Hz, ^3^ *J*_*H‐H*_ = 8 Hz, 1 H, H^4/9^), 7.20 (s, 1 H, H^11^), 7.05 (s, 1 H, H^2^), 5.74 (d, ^2^ *J*_*H‐H*_ = 16 Hz, 1 H, H^17^), 5.58 (d, ^2^ *J*_*H‐H*_ = 16 Hz, 1 H, H^17^), 3.83 (s, 2 H, H^21^), 1.27 (s, 9 H, (CH_3_)_3_^*t*Bu^), 1.26 (s, 9 H, (CH_3_)_3_^*t*Bu^). ^13^C NMR (75 MHz, CD_3_CN, 300 K): *δ* 182.2 (s, C^18^), 161.4 (s, C^6/7^), 152.3 (s, C^1/12^), 151.0 (s, C^1/12^), 151.0 (s, C^3/10^ + C^16^), 150.8 (s, C^3/10^), 149.2 (s, C^14^), 148.4 (s, C^15^), 146.4 (s, C^6/7^), 146.0 (s, C^13^), 133.3 (s, C^2/11^), 129.7 (s, C^2/11^), 126.5 (s, C^4/9^), 125.9 (s, C^4/9^), 125.4 (s, C^20^), 124.2 (s, C^19^), 122.8 (s, C^5/8^), 121.8 (s, C^5/8^), 53.3 (s, C^17^), 39.0 (s, C^21^), 35.7 (s, C(CH_3_)_3_^*t*Bu^), 35.2 (s, C(CH_3_)_3_^*t*Bu^), 31.4 (s, 2*(CH_3_)_3_^*t*Bu^). C^16^ not visible. HRMS (ESI) m/z: [*M*‐PF_6_]^+^ Calcd for C_29_H_34_N_4_Au^+^ 635.2444. Found 635.2468; (Error: 3.8 ppm). Calcd for C_29_H_34_N_4_AuPF_6_.2H_2_O: C, 42.66; H, 4.69; N, 6,86 found: C, 42.37; H, 4.19; N, 6.48.

**BGC18b**: Final yield 63 mg, 0.075 mmol, quant. (grayish powder). ^1^H NMR (300 MHz, CD_3_CN, 300 K): *δ* 8.90 (d, ^3^ *J*_*H‐H*_ = 5 Hz, 1 H, H^13^), 8.23 (dt, ^4^ *J*_*H‐H*_ = 2 Hz, ^3^ *J*_*H‐H*_ = 8 Hz, 1 H, H^15^), 8.06 (d, ^3^ *J*_*H‐H*_ = 8 Hz, 1 H, H^16^), 7.97 (dd, ^4^ *J*_*H‐H*_ = 2 Hz, ^3^ *J*_*H‐H*_ = 7 Hz, 1 H, H^24^), 7.84 (ddd, ^4^ *J*_*H‐H*_ = 1 Hz, ^3^ *J*_*H‐H*_ = 5 Hz, ^3^ *J*_*H‐H*_ = 8 Hz, 1 H, H^14^), 7.77 (dd, ^4^ *J*_*H‐H*_ = 2 Hz, ^3^ *J*_*H‐H*_ = 7 Hz, 1 H, H^21^), 7.60 (m, 2 H, H^22^ + H^23^), 7.44 (d, ^3^ *J*_H‐H_ = 8 Hz, 2 H, H^5^ + H^8^), 7.37−7.27 (m, 3 H, H^4^ + H^9^ + H^11^), 7.07 (d, ^4^ *J*_*H‐H*_ = 2 Hz, 1 H, H^2^), 5.94 (d, ^2^ *J*_*H‐H*_ = 16 Hz, 1 H, H^18^), 5.87 (d, ^*2*^ *J*_*H‐H*_ = 16 Hz, 1 H, H^18^), 4.06 (s, 3 H, H^26^), 1.26 (s, 18 H, 2*(CH_3_)_3_^*t*Bu^). ^13^C NMR (75 MHz, CD_3_CN, 300 K): *δ* 190.8 (s, C^19^), 161.4 (s, C^6/7^), 154.5 (s, C^17^), 153.4 (s, C^13^), 152.6 (s, C^1/12^), 151.5 (s, C^1/12^), 151.2 (s, C^3/10^), 150.8 (s, C^3/10^), 146.9 (s, C^6/7^), 143.7 (s, C^15^), 136.2 (s, C^25^), 134.5 (s, C^20^), 133.9 (s, C^2/11^), 130.3 (s, C^2/11^), 127.7 (s, C^14^), 127.5 (s, C^5/8^), 126.4 (s, C^4/9^ + C^22^), 126.2 (s, C^23^), 126.0 (s, C^4/9^), 122.8 (s, C^5/8^), 121.9 (s, C^5/8^), 113.7 (s, C^21^), 112.5 (s, C^24^), 52.5 (s, C^18^), 36.7 (s, C^26^), 35.7 (s, C(CH_3_)_3_^*t*Bu^), 35.4 (s, C(CH_3_)_3_^*t*Bu^), 31.5 (s, (CH_3_)_3_^*t*Bu^), 31.5 (s, (CH_3_)_3_^*t*Bu^). HRMS (ESI) m/z: [*M*‐PF_6_]^+^ Calcd for C_34_H_37_N_3_Au^+^ 684.2648. Found 684.2646; (Error: −0.2 ppm). Calcd for C_34_H_37_N_3_AuPF_6_.2H_2_O: C, 47.18; H, 4.77; N, 4.85 found: C, 47.49; H, 4.48; N, 4.63.

**BGC19b**: Final yield 65 mg, 0.08 mg, quant. (grayish solid). ^1^H NMR (300 MHz, CD_3_CN, 300 K): *δ* 8.87 (d, ^3^ *J*_*H‐H*_ = 6 Hz, 1 H, H^13^), 8.24 (m, 2 H, H^15^ + H^25^), 8.00 (d+s, ^3^ *J*_*H‐H*_ = 8 Hz, 2 H, H^16^+H^20^), 7.82 (ddd, ^4^ *J*_*H‐H*_ = 1 Hz, ^3^ *J*_*H‐H*_ = 6 Hz, ^3^ *J*_*H‐H*_ = 7 Hz, 1 H, H^14^), 7.66 (d, ^3^ *J*_*H‐H*_ = 9 Hz, 1 H, H^22^), 7.40 (d, ^3^ *J*_H‐H_ = 8 Hz, 2 H, H^5^+H^8^), 7.29 (dd, ^3^ *J*_*H‐H*_ = 2 Hz, ^3^ *J*_*H‐H*_ = 8 Hz, 1 H, H^4/9^), 7.27 (dd, ^3^ *J*_*H‐H*_ = 2 Hz, ^3^ *J*_*H‐H*_ = 8 Hz, 1 H, H^4/9^), 7.19 (d, ^4^ *J*_*H‐H*_ = 2 Hz, 1 H, H^11^), 7.09 (d, ^4^ *J*_*H‐H*_ = 2 Hz, 1 H, H^2^), 7.07 (dd, ^3^ *J*_*H‐H*_ = 7 Hz, ^3^ *J*_*H‐H*_ = 9 Hz, 1 H, H^23^), 6.85 (td, ^4^ *J*_*H‐H*_ = 1 Hz, ^3^ *J*_*H‐H*_ = 7 Hz, 1 H, H^24^), 5.98 (d, ^2^ *J*_*H‐H*_ = 15 Hz, 1 H, H^18^), 5.72 (d, ^2^ *J*_*H‐H*_ = 15 Hz, 1 H, H^18^), 1.25 (s, 9 H, (CH_3_)_3_^*t*Bu^), 1.13 (s, 9 H, (CH_3_)_3_^*t*Bu^). ^13^C NMR (75 MHz, CD_3_CN, 300 K): δ 171.6 (s, C^19^), 161.4 (s, C^6/7^), 154.1 (s, C^17^), 153.4 (s, C^13^), 152.5 (s, C^1/12^), 151.4 (s, C^1/12^), 151.0 (s, C^3/10^), 150.7 (s, C^3/10^), 146.4 (s, C^6/7^), 143.5 (s, C^15^), 134.1 (s, C^2/11^), 132.7 (s, C^21^), 130.3 (s, C^2/11^), 127.8 (s, C^16^), 127.7 (s, C^14^+C^25^), 126.3 (s, C^4/9^), 125.9 (s, C^4/9^), 124.5 (s, C^23^), 122.7 (s, C^5/8^), 121.8 (s, C^5/8^), 119.5 (s, C^22^), 116.6 (s, C^24^), 115.0 (s, C^20^), 56.8 (s, C^18^), 35.7 (s, C(CH_3_)_3_^*t*Bu^), 35.2 (s, C(CH_3_)_3_^*t*Bu^), 31.5 (s, (CH_3_)_3_^*t*Bu^), 31.2 (s, (CH_3_)_3_^*t*Bu^). HRMS (ESI) m/z: [*M*‐PF_6_]^+^ Calcd for C_33_H_35_N_3_Au^+^ 670.2491. Found 670.2495; (Error: 0.6 ppm). Calcd for C_33_H_35_N_3_AuPF_6_: C, 48.60; H, 4.33; N, 5.15 found: C, 48.26; H, 5.70; N, 3.33.

##### X‐Ray Crystal Structure Determination

Single crystals of **BGC12a**, **BGC16a**, **BGC19a**, **BGC13b**, **BGC16b**, and **BGC18b** were selected, mounted onto a cryoloop, and transferred into a cold nitrogen gas stream. For **BGC19a**, **BGC16b**, and **BGC18b**, intensity data were collected with a Bruker Kappa‐APEX2 CCD diffractometer using a graphite‐monochromated MoKα radiation (**BGC16b**) or a microfocused CuKα radiation (**BGC19a** and **BGC18b**). Data collections, unit‐cell parameter determinations, integration, and data reductions were performed with the Bruker APEX/SAINT^[^ ^55^ ^]^ suite at 200 K. For **BGC12a**, **BGC16a**, and **BGC13b,** intensity data were collected with a Rigaku Oxford Diffraction Xcalibur‐S four‐circle diffractometer equipped with a CCD area‐detector and graphite monochromated MoK*α* radiation. Data collections, unit‐cell parameter determinations, integration, and data reductions were performed with the Rigaku CrysalisPro^[^ ^56^ ^]^ suite at 200 K. All structures were solved with SHELXT^[^ ^57^ ^]^ and refined anisotropically by full‐matrix least‐squares methods with SHELXL,^[^ ^58^ ^]^ using the Olex2^[^ ^59^ ^]^ software (except H atoms). The structures were deposited at the Cambridge Crystallographic Data Centre with numbers CCDC 2 420 678–2 420 683 and can be obtained free of charge via www.ccdc.cam.ac.UK.

##### NMR Reactivity Assays

**Solvolysis Kinetics:** 400 μL of freshly prepared gold complex solution in DMSO‐d_6_ (5 mM) was transferred to an NMR tube. Spectra were acquired straight away (t_0_ reference) prior to transferring the tubes to an oven set at 37 °C for 72 h. Spectra were then acquired periodically during incubation. The same procedure was repeated with addition of 100 μL of D_2_O to each tube.

**Solvolysis:** A concentrated solution of gold complex (≈40 mM) was prepared in 1 mL DMSO‐d_6_ mixed with 250 μL of D_2_O and incubated at 37 °C for 24 h. The solution was then extracted 5 times with 3 mL DCM. The DCM phase was recovered and evaporated thoroughly, and the resulting residue was dissolved in MeCN‐d_3_ and analyzed by ^1^H NMR.

**Reaction with Chloride Ions:** 400 μL of freshly prepared gold complex solution in DMSO‐d_6_ (5 mM) was added to an NMR tube, followed by 4 μL of 0.5 M NaCl in D_2_O and 96 μL of D_2_O. Spectra were acquired straight away (t_0_ reference) prior to transferring the tubes to an oven set at 37 °C for 72 h. Spectra were then acquired periodically during incubation.

**Reaction with Amino Acids:** Solutions of the gold complexes (10 mM) and N‐acetyl amino acids (10 mM) were freshly prepared in DMSO‐d_6_. Samples were prepared by adding 200 μL of gold complex solution along with 200 μL of freshly prepared N‐acetyl amino acid solution in an NMR tube. A control sample was prepared for each gold complex by diluting 200 μL of complex solution in 200 μL of DMSO‐d_6_. ^1^H NMR spectra were recorded immediately after dilution. The tubes were then kept in an oven set at 37 °C, and spectra were recorded periodically until 72 h of incubation.

**Speciation in DMEM:** NMR spectra were recorded on a **Bruker Avance II** 300 spectrometer (7 T) operating at a ^1^H Larmor frequency of 300.13 MHz with a 5 mm observe broadband probe (QNP) z‐axis (^31^P/^19^ F/^13^C/^1^H) at 27 °C.

Solutions of the gold complexes (10 mM) were freshly prepared in DMSO‐d_6_. Samples were prepared by mixing 200 μL of complex solution with 100 μL (resp. 67 μL) of pure DMSO‐d_6_ and 100 μL (resp. 133 μL) of DMEM GlutaMax High glucose without FBS. ^1^H NMR spectra were acquired immediately after mixing (t < 5 min) using a standard zg30 sequence to determine the resonant frequency of water in the tube. Further spectra were acquired using a cpmg‐esgp1d sequence (pulse program available^[^ ^60^ ^]^) which carrier frequency (O1P) was tuned to the water peak maximum to ensure optimal water suppression. The Hahn echo time (D20) was left null to acquire a spectrum without the use of T_2_‐gating. A particular attention was paid to the shimming process as poor shims result in a broader water signal thus leading to an inefficient water suppression.

##### Cell Culture and Viability Measurements

HeLa and MDA‐MB‐231 cells were cultivated in DMEM GlutaMax High Glucose supplemented with 10 vol% fetal bovine serum (FBS) and 1 vol% penicillin/streptomycin (50 U/mL and 50 μg/mL respectively). Huh‐7 cells were cultivated in MEM supplemented with 10 vol% FBS, GlutaMax (2 mM), sodium pyruvate (1 mM), nonessential amino acids mix (0.1 mM), and 1 vol% penicillin/streptomycin. MCF10A cells were cultivated in DMEM:F12 GlutaMax supplemented with 1 vol% penicillin/streptomycin, hEGF (20 ng/mL), hydrocortisone (500 μg/mL), cholera toxin (100 ng/mL), insulin (10 μg/mL), and 5 vol% horse serum. These media will be further referred as “Complete media.”

Stock solutions of the gold complexes (20 mM) were prepared in DMSO and kept at −20 °C. Serial dilutions were performed to obtain a set of 8 concentrations with a 2‐fold geometric progression, and were stored at −20 °C as well.

##### Cellular Proliferation Assay

Cell proliferation assays were performed using resazurin as a viability indicator. Cells were seeded in 96‐well plates (2000 cells/100 μL per well) in the appropriate complete medium and allowed to grow for 24 h. Cells were then treated in triplicate with 8 different concentrations of the complexes, which were added as 100 μL of 2x dilution in culture medium containing no FBS (or containing 5% horse serum for MCF‐10 A cells), thus leading to a 5% concentration of serum in each well. After 72 h incubation, 110 μL of supernatant was removed from each well, and 10 μL of 500 μM resazurin solution (10x) in PBS were added prior to incubating the cells for 2 h. The fluorescence intensity of the reduction product resorufin was measured in each well (540 nm excitation, 590 nm reading), and cellular viability was computed as the fluorescence ratio between sample and DMSO control wells after blank subtraction. The EC_50_ value was computed by fitting the data with a 4‐parameter sigmoid *σ* through nonlinear least square regression, where the EC_50_ value is directly the *c* parameter. The regression was performed using curve_fit() function *scipy.optimize* module in python (scripts available^[^ ^60^ ^]^).

$$\sigma \left(x,a,b,c,d\right)=d+\frac{a-d}{1+{\left(\frac{x}{c}\right)}^{b}}$$

##### Log P Determination by HPLC

Water/octanol partition coefficients log(k′_w_) were determined by a reverse‐phase HPLC method from OECD.^[^ ^61^ ^]^ The stationary phase was a Nucleodur C18 Gravity 5 μm, 4.6 × 150 mm column (Macherey‐Nagel). For neutral complexes **BGC12‐19a**, mobile phase was set as 25% 1 mM phosphate buffer, pH 7.4 in acetonitrile in order to keep the pyridine in its free base state (condition A). For cationic complexes **BGC12‐19b**, mobile phase was set as 25% water in acetonitrile to prevent any species from substituting the coordinated pyridine (condition B). All eluents were filtered through a 0.22 μm nylon membrane before use. Flow rate was set to 1 mL/min. Compounds solutions were prepared as 500 μM solutions in acetonitrile spiked with 50 μM (neutral complexes) or 100 μM (cationic complexes) uracil as a null retention marker.

A standard mix was prepared by combining 8 molecules of known Log(k′_w_) and uracil, as described in Table S2, Supporting Information. This standard mix was used to compute a calibration curve of LogP = f(log(k)) in both elution conditions A and B (Figure S5), where $log\left(k\right)=log\left(\frac{t_{R}-t_{0}}{t_{0}}\right)$, *k* being the retention factor of a given peak, *t*_*R*_ the retention time of the corresponding peak and *t*_0_ the null retention time.

Retention times were measured on ChromNAV control center, and the log k′_w_ was determined by linear extrapolation using equation described on Figure S5, Supporting Information, to yield the log k′_w_ presented in Table S3, Supporting Information.

##### Mitochondrial Membrane Polarization Assay

HeLa cells were seeded in a 96‐well plate (2000 cells in 100 μL complete medium per well) and left to adhere. Cells were then treated in triplicate with gold complexes (addition of 100 μL of 2x complex solution in FBS‐free medium) 4 h and 1 h before reading the plate. A control triplicate was treated with **CCCP** (10 μM) and incubated for 4 h before reading. One hour before reading, 100 μL of supernatant was withdrawn from each well and replaced with JC‐10 in *assay buffer A* solution (Abcam, 1x, 50 μL). Cells were incubated with the marker for 1 h, and *assay buffer B* (50 μL) was added. Mitochondrial membrane potential was assessed by computing the ratio of fluorescence intensities between aggregated (*λ*_ex_ = 540 nm, *λ*_fluo_ = 590 nm) and nonaggregated JC‐10 (*λ*_ex_ = 490 nm, *λ*_fluo_ = 525 nm). To correlate overall cell viability to mitochondrial function, rezasurin assay was carried out in parallel: one hour before reading, 110 μL of medium were removed, and 10 μL of 500 μM rezasurin solution was added to each well, followed by incubation at 37 °C until reading.

##### Caspase Activity and Cellular Morphology Monitoring

HeLa cells were seeded in an 8‐well Ibidi slide (4000 cells in 500 μL complete medium per well) and allowed to grow for 24 h. The culture medium was changed to a fresh one (DMEM 5% FBS, 200 μL) containing SiR‐DNA dye (Spirochrome, 1 μM) and CellEvent Caspase 3/7 Green marker (ThermoFisher, 1 μM). Cells were incubated with the markers for 2 h and then treated with gold complexes at their respective EC_50_ concentrations (supplied as 200 μL of 2x solutions in DMEM 5% FBS). Cells were monitored for 24 h (20 min step between images) by phase contrast and fluorescence microscopy (Cy5 and EGFP filters for SiR‐DNA and CellEvent Caspase 3/7 markers, respectively). Images were processed using Fiji, and cellular viabilities were extracted as $Viability=1-\frac{N_{EGFP}}{N_{Cy5}}$ where *N*_*i*_ denotes the number of cells counted in channel *i*. Cells were counted in EGFP and Cy5 channels using TrackMate (*DoG detector* + *Simple LAP tracker*).^[^ ^62^ ^]^ The cellular morphology was also manually monitored using the phase contrast channel by tagging cells losing adhesion and displaying blebs.^[^ ^60^ ^]^

## Conflict of Interest

The authors declare no conflict of interest.

## Supporting information

Supplementary Material

## Data Availability

The major part of the data supporting this article is available in the main text and in the supplementary information. Additional data such as the raw videomicroscopy data, custom cpmg‐esgp1d NMR pulse program and python script used to perform nonlinear fits are publicly available in the “Recherche Data Gouv” repository (Sorbonne Université subset) at https://doi.org/10.57745/PQTFRY (accessed on 18 March 2025). See the README.md files for details on the content of the repository.
