# Supplementary material for: Impact of Chelation on Reactivity and Cytotoxicity of Hemilabile Biphenyl Gold(III) N‐Heterocyclic Carbene Complexes
Source: ChemMedChem. 2025 Jun 14;20(14):e202500302. doi: 10.1002/cmdc.202500302 (PMC12276038; doi:10.1002/cmdc.202500302)
Supplement: Supplementary file 1 — Supplementary Material [file CMDC-20-e202500302-s001.pdf]

Electronic Supporting Information

for

Impact of chelation on reactivity and cytotoxicity of hemilabile biphenyl gold(III) N-heterocyclic carbene complexes

Tom Lacoma,[a,b] Jérémy Forté,[a] Régina Maruchenko,[a] Michèle Salmain,[a*] Joëlle Sobczak-Thépot[b]* and Benoît Bertrand[a]*

[a] Institut Parisien de Chimie Moléculaire (IPCM), Sorbonne Université, CNRS, 4 place Jussieu, F-75005 Paris, France.

E-mail: benoit.bertrand@sorbonne-universite.fr; michele.salmain@sorbonne-universite.fr

[b] Centre de Recherche Saint Antoine (CRSA), Sorbonne Université, INSERM, 184 rue du Faubourg Saint Antoine, F-75012 Paris, France.

E-mail: joelle.sobczak@inserm.fr

Content	Page
**Figure S1-4**: Crystal structures of complexes **BGN12a**, **BGN18a**, **BGN13b** and **BGN18b**	S3-4
**Table S1**: Au-C_3 and Au-N bond length for complexes **BGC12a**, **BGC16a**, **BGC18a**, **BGN13b**, **BGC16b** and **BGC18b**.	S5
**Table S2**: Composition of the logP standard mix	S5
**Figure S5:** (A) Calibration curve computed from standard mix for neutral complexes. (B) Calibration curve computed from standard mix for cationic complexes.	S5
**Table S3**: Measured logP values for complexes **BGC12-19a/b**	S5
**Figure S6-13:** ^{1}H NMR spectrum of complex **BGC12a-BGC19a** in DMSO-d_6 after preparation and after 72h of incubation at 37°C	S5-9
**Figure S14-21:** ^{1}H NMR spectrum of complex **BGC12b-BGC19b** in DMSO-d_6 after preparation and after 72h of incubation at 37°C	S9-13
**Figure S22-29:** ^{1}H NMR spectrum of complex **BGC12a-BGC19a** in DMSO-d_6/D_2O mix (4/1 v/v) after preparation and after 72h of incubation at 37°C	S13-17
**Figure S30-37:** ^{1}H NMR spectrum of complex **BGC12b-BGC19b** in DMSO-d_6/D_2O mix (4/1 v/v) after preparation and after 72h of incubation at 37°C	S17-21
**Figure S38-40:** ^{1}H NMR spectrum of complex **BGC15a/b**, **BGC19a/b** and **BGC0a/b** in DMSO-d_6/D_2O (4:1 v/v) in presence or absence of NaCl (1eq)	S21-22
**Figure S41:** ^{1}H NMR spectra of **BGC12a/b** in DMSO-d_6/DMEM (3:1 v/v) using CPMGR-ESPG1D water suppression sequence	S23
**Figure S42-49**: ^{1}H NMR spectra recorded at 5 mM in DMSO-d_6 immediately after mixing of A) **BGC12-19a** alone and reacted with 1 eq. of N-Ac-methionine, N-Ac-histidine and N-Ac-cysteine; B) **BGC12-19b** alone and reacted with 1 eq. of N-Ac-methionine, N-Ac-histidine and N-Ac-cysteine.	S24-31
**Fig S50:** Structures of the products and HRMS reports from the reaction of (A) BGC12b and N-acetylcysteine (1 eq.) and (B) BGC12b and N-acetylhistidine (1 eq.)	S32
**Table S4**: ^{1}H NMR conversion rates of the reaction of **BGC** complex with 1 equivalent of N-Acetyl amino acid straight after mixing.	S33
**Figure S51:** Cellular viability of HeLa cells treated with DMSO (4h, normalization reference), **BGC19b** (10µM, 4h and 1h) and **CCCP** (10µM, 4h). Viability measured using resazurin assay as described in the experimental part.	S33
**Figure S52-S67**: ^{1}H and ^{13}C{^{1}H} spectra of complexes **BGC12a-19a**	S34-S41
**Figure S68-S83**: ^{1}H and ^{13}C{^{1}H} spectra of complexes **BGC12b-19b**	S42-S49
**Table S5.** Crystallographic data for **BGC12a**, **BGC16a**, **BGC18a** and **BGC13b**, **BGC16b**, **BGC18b**.	S50

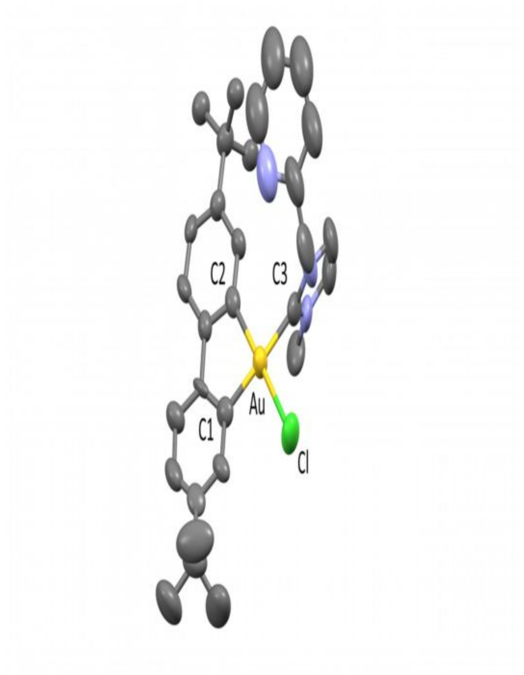

Figure S1: Crystal structure of one of the two molecule of the asymmetric unit of **BGC12a**. Ellipsoids set at 50% probability. Hydrogen atoms and solvent molecule have been omitted for clarity. Selected bond distances [Å] and angles [°] measured at 200 K: Au-C_1 2.00(2), Au-C_2 2.03(2), Au-C_3 2.08(3), Au-Cl 2.377(7) and C_1-Au-C_2 81.8(9), C_2-Au-C_3 92.9(9), C_3-Au-Cl 89.5(7), Cl-Au-C_1 96.0(7).

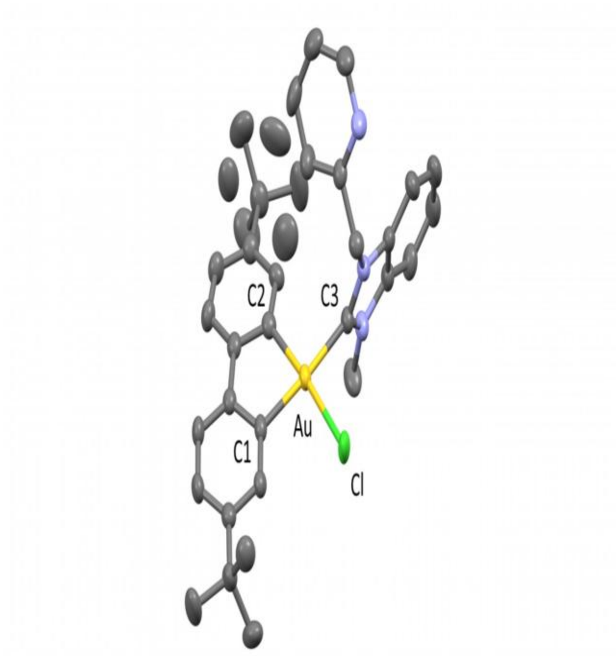

Figure S2: Crystal structure of **BGC18a**. Ellipsoids set at 50% probability. Hydrogen atoms and solvent molecule have been omitted for clarity. Selected bond distances [Å] and angles [°] measured at 200 K: Au-C_1 2.064(3), Au-C_2 2.036(3), Au-C_3 2.091(3), Au-Cl 2.3765(7) and C_1-Au-C_2 80.96(12), C_2-Au-C_3 94.67(12), C_3-Au-Cl 89.59(8), Cl-Au-C_1 94.83(8).

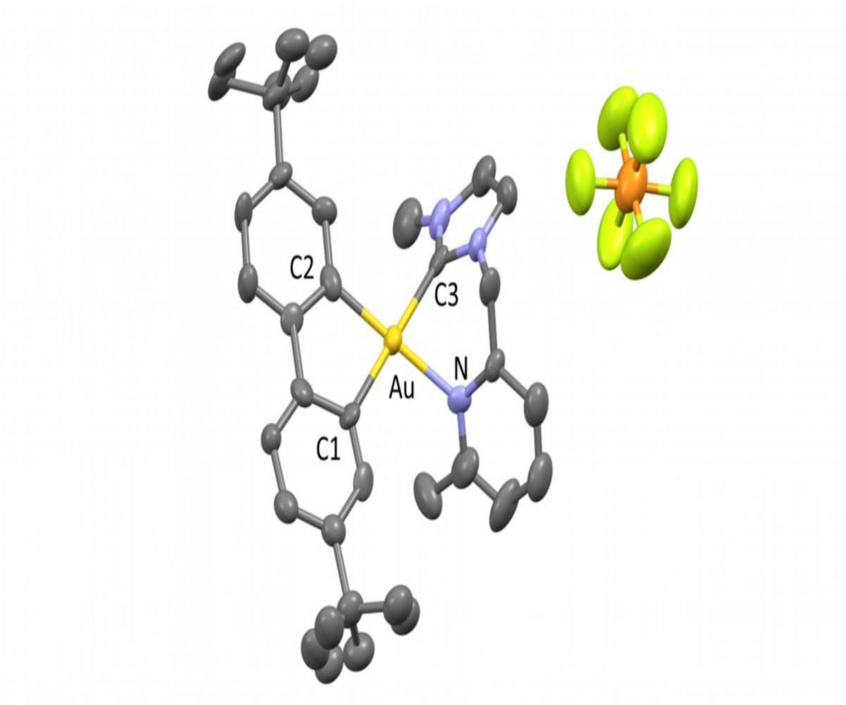

Figure S3: Crystal structure of **BGC13b**. Ellipsoids set at 50% probability. Hydrogen atoms have been omitted for clarity. Selected bond distances [Å] and angles [°] measured at 200 K: Au-C_1 2.058(14), Au-C_2 2.012(15), Au-C_3 2.065(14), Au-N 2.144(12) and C_1-Au-C_2 80.9(6), C_2-Au-C_3 99.7(6), C_3-Au-N 81.6(5), N-Au-C_1 97.3(5).

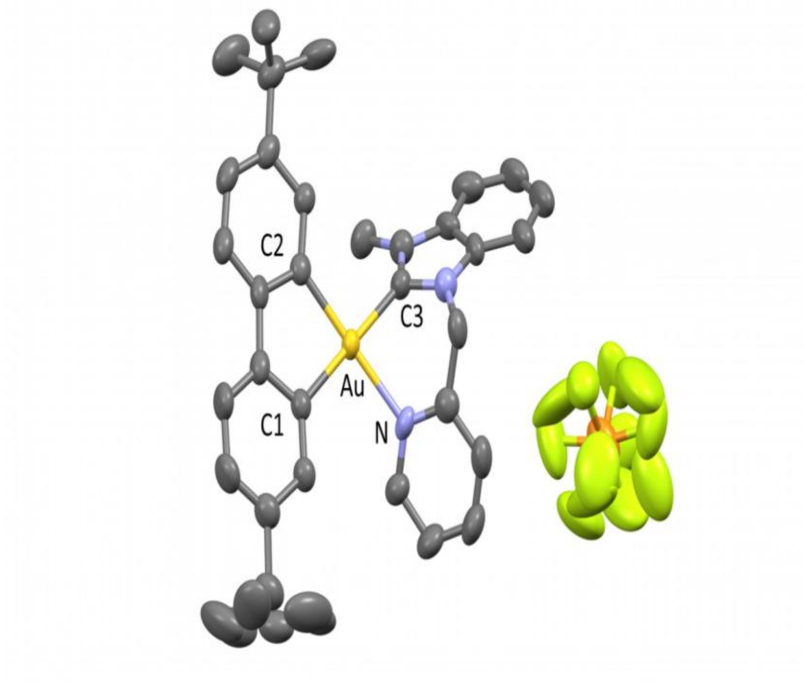

Figure S4: Crystal structure of **BGC18b**. Ellipsoids set at 50% probability. Hydrogen atoms have been omitted for clarity. Selected bond distances [Å] and angles [°] measured at 200 K: Au-C_1 2.076(5), Au-C_2 2.036(5), Au-C_3 2.068(5), Au-N 2.161(5) and C_1-Au-C_2 81.6(2), C_2-Au-C_3 98.6(2), C_3-Au-N 82.52(19), N-Au-C_1 97.2(2).

Table S1: Au-C_3 and Au-N bond length for complexes **BGC12a**, **BGC16a**, **BGC18a**, **BGC13b**, **BGC16b** and **BGC18b**.

Complexes	Au-C_3 bond length (Å)	Au-N bond length (Å)
**BGC12a**	2.08(3)<br>2.07(2)	-
**BGC16a**	2.079(4)<br>2.065(4)	-
**BGC18a**	2.091(3)	-
**BGC13b**	2.065(14)	2.144(2)
**BGC16b**	2.065(4)	2.169(3)
**BGC18b**	2.068(5)	2.161(5)

Table S2: Composition of the log P standard mix

**Compound**	**Concentration (mM)**	**log(k'$_w$)**
**Uracile**	0.01	N/A
**Benzonitrile**	1	1.6
**Anisole**	0.5	2.1
**Toluene**	1	2.7
**Naphtalene**	0.1	3.6
**Diphenylether**	0.5	4.2
**Butylbenzene**	1	4.6
**Triphenylamine**	1	5.7
**Octylbenzene**	1	6.3

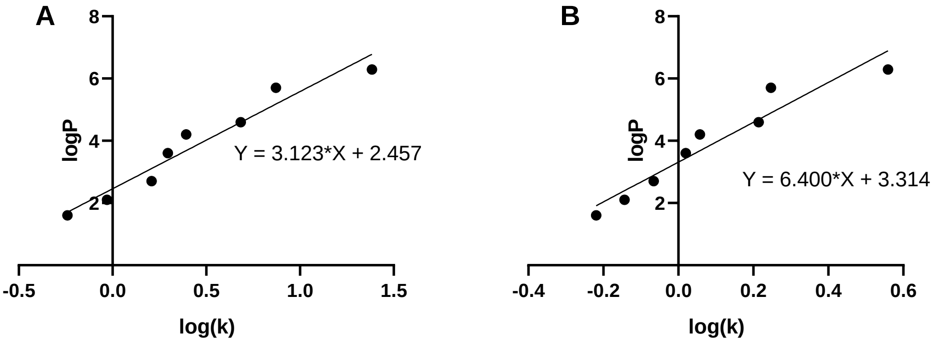

Figure S5 : (A) Calibration curve computed from standard mix for neutral complexes. (B) Calibration curve computed from standard mix for cationic complexes.

Table S3: Measured log P values for complexes **BGC12-19a/b**

Compound	Log P (neutral, **a** form)	Log P (cationic **b**, form)
**BGC12**	4.7	3.9
**BGC13**	5.3	4.4
**BGC14**	5.2	4.8
**BGC15**	4.8	4.2
**BGC16**	5.7	4.5
**BGC17**	4.4	3.5
**BGC18**	5.7	4.5
**BGC19**	5.3	4.3

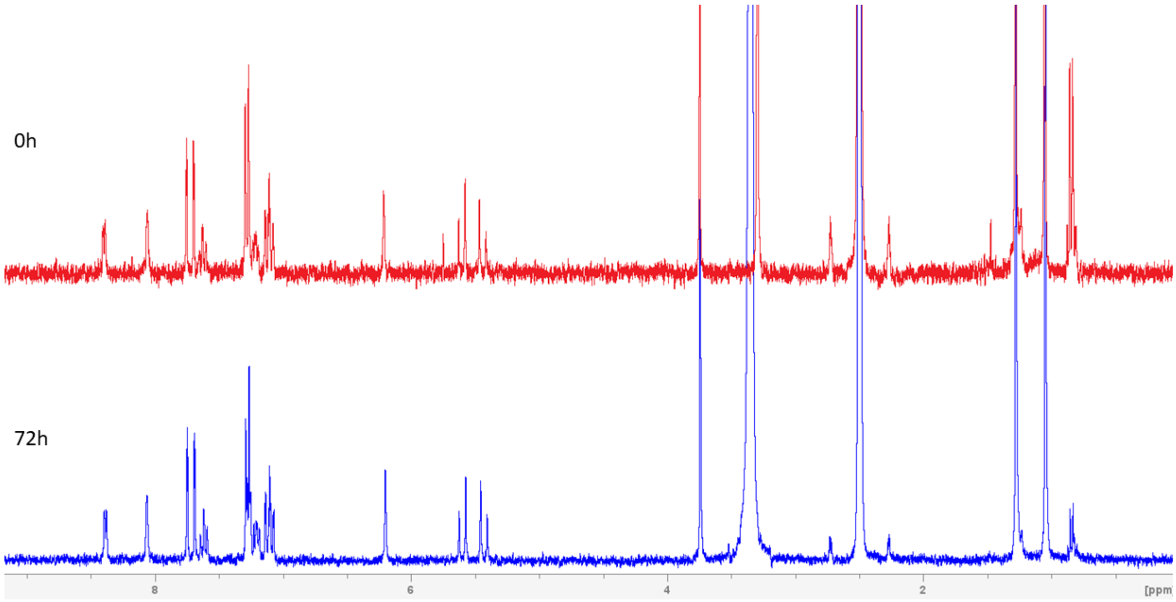

Figure S6: ^{1}H NMR spectrum of complex **BGC12a** in DMSO-d_6 right after the preparation and after 72 h of incubation at 37°C

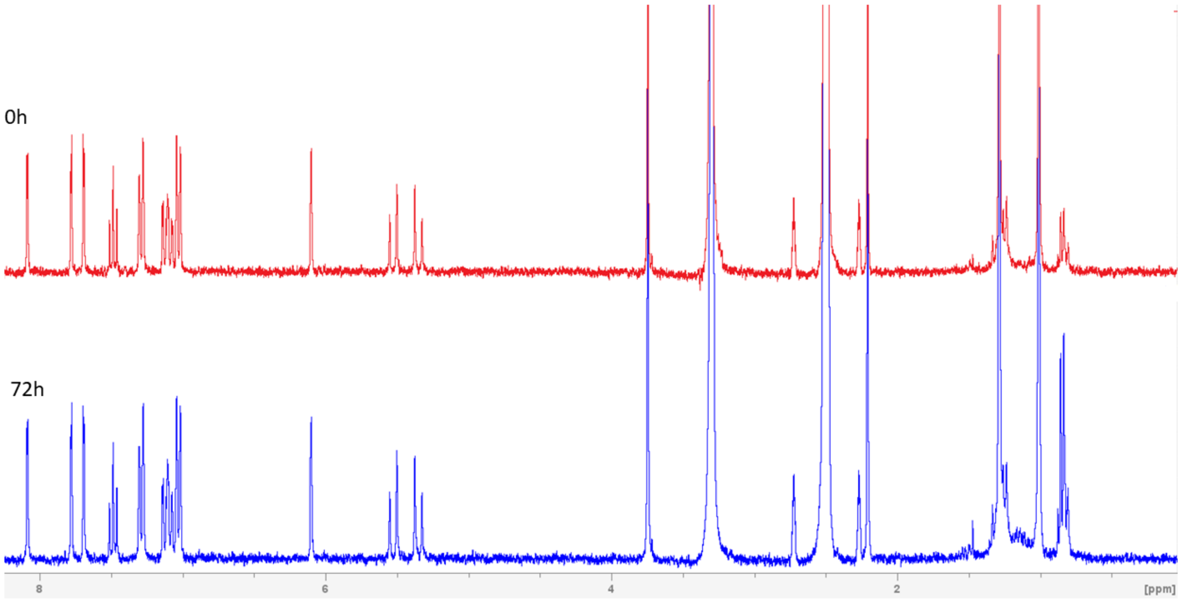

Figure S7: ^{1}H NMR spectrum of complex **BGC13a** in DMSO-d_6 right after the preparation and after 72 h of incubation at 37°C

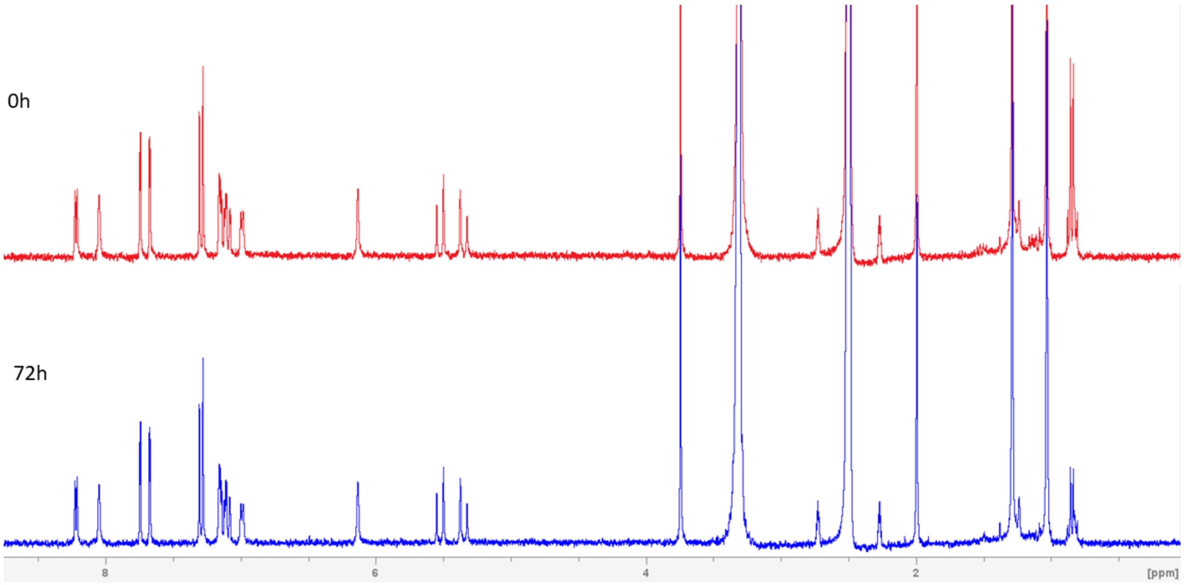

Figure S8: ^{1}H NMR spectrum of complex **BGC14a** in DMSO-d_6 right after the preparation and after 72 h of incubation at 37°C

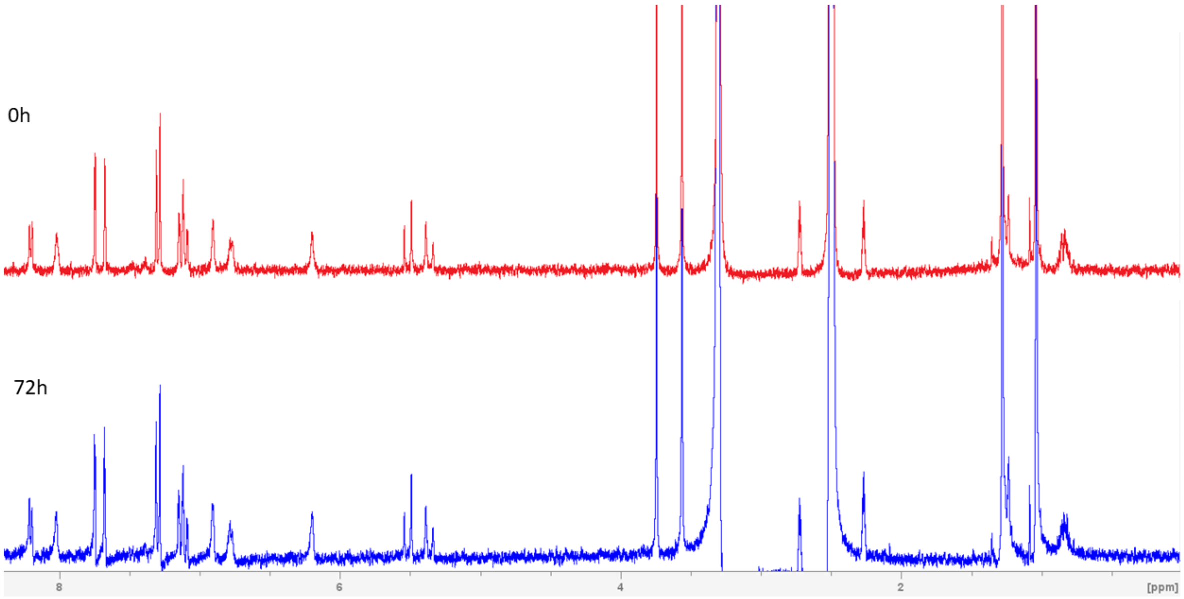

Figure S9: ^{1}H NMR spectrum of complex **BGC15a** in DMSO-d_6 right after the preparation and after 72 h of incubation at 37°C

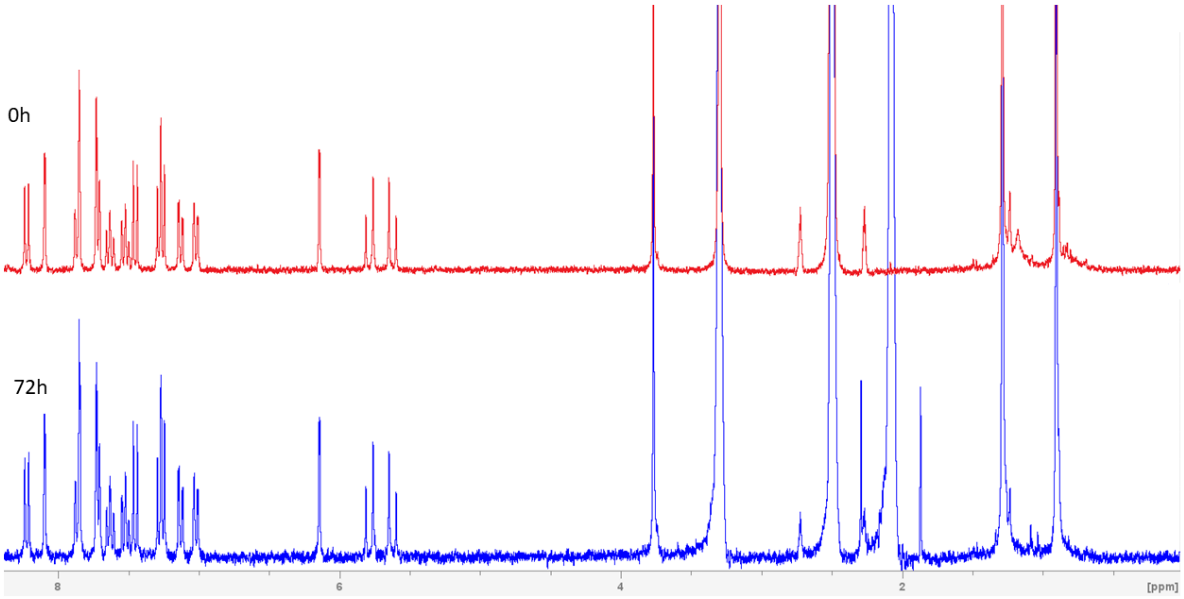

Figure S10: ^{1}H NMR spectrum of complex **BGC16a** in DMSO-d_6 right after the preparation and after 72 h of incubation at 37°C

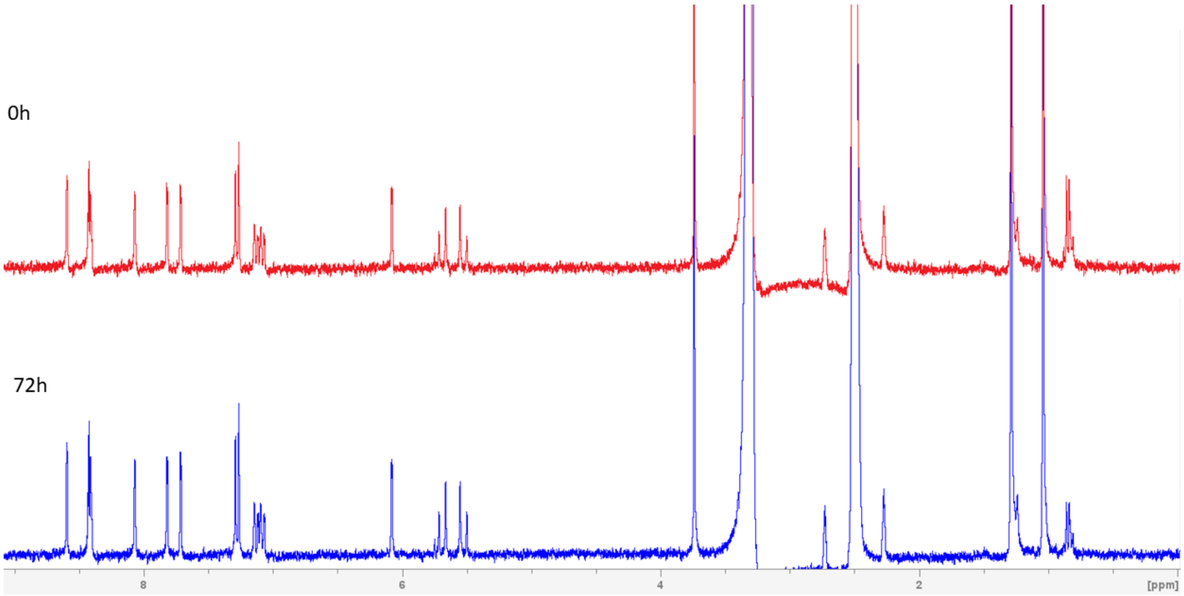

Figure S11: ^{1}H NMR spectrum of complex **BGC17a** in DMSO-d_6 right after the preparation and after 72 h of incubation at 37°C

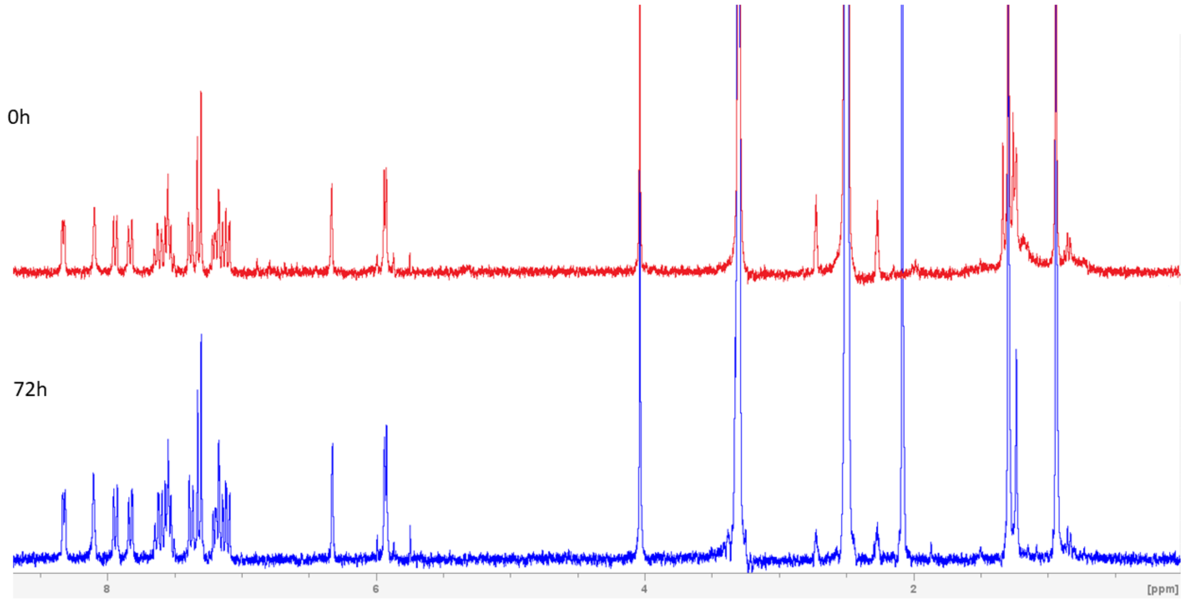

Figure S12: ^{1}H NMR spectrum of complex **BGC18a** in DMSO-d_6 right after the preparation and after 72 h of incubation at 37°C

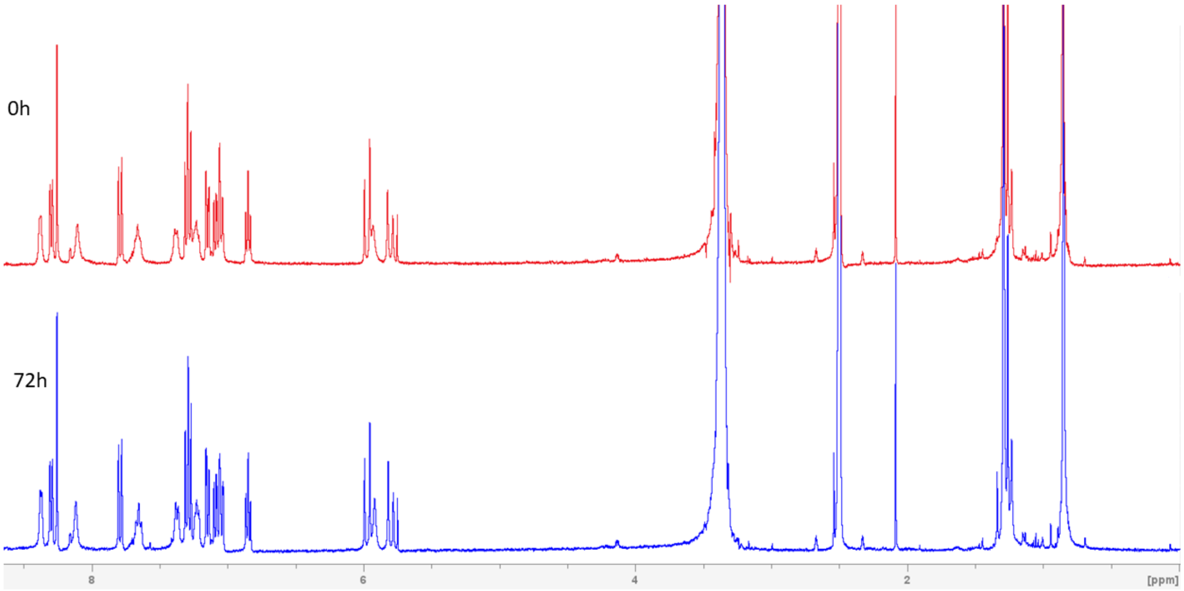

Figure S13: ^{1}H NMR spectrum of complex **BGC19a** in DMSO-d_6 right after the preparation and after 72 h of incubation at 37°C

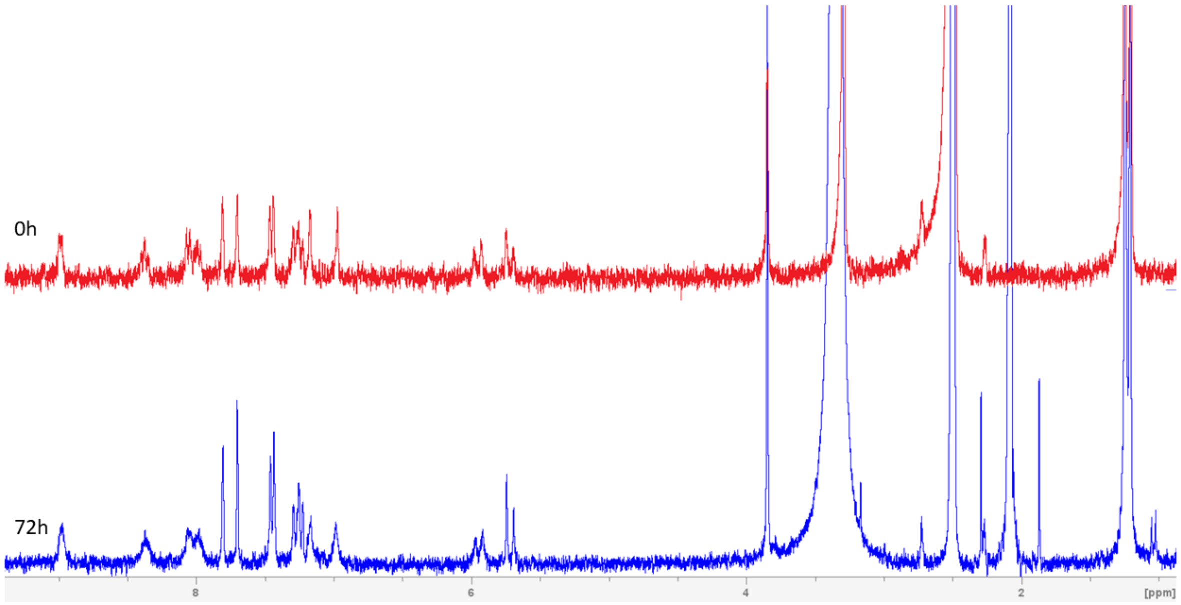

Figure S14: ^{1}H NMR spectrum of complex **BGC12b** in DMSO-d_6 right after the preparation and after 72 h of incubation at 37°C

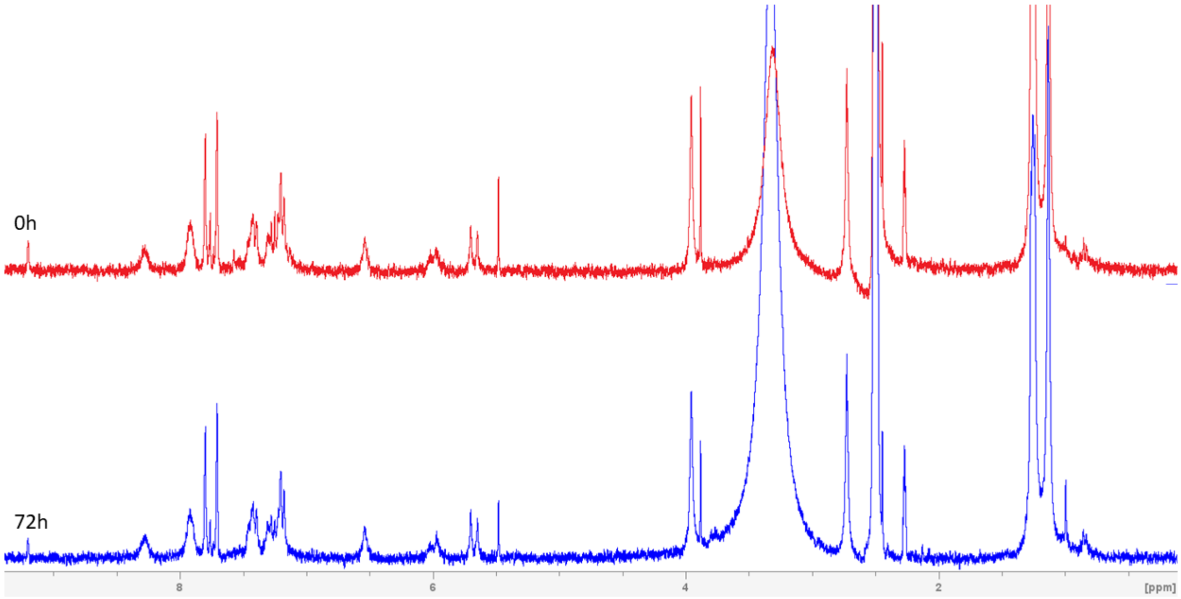

Figure S15: ^{1}H NMR spectrum of complex **BGC13b** in DMSO-d_6 right after the preparation and after 72 h of incubation at 37°C

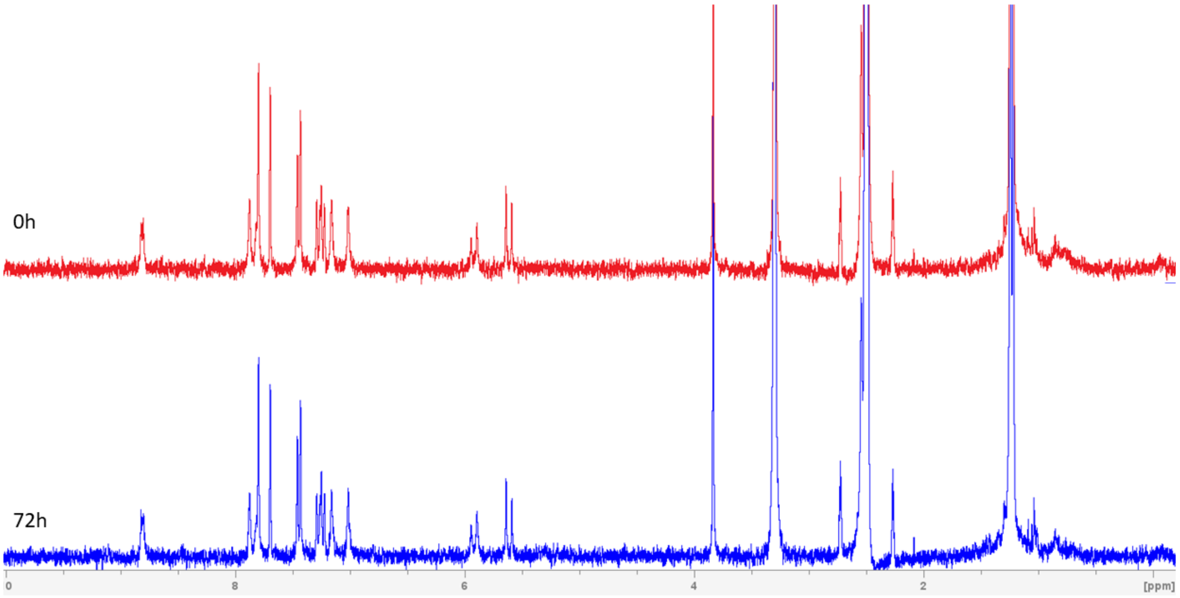

Figure S16: ^{1}H NMR spectrum of complex **BGC14b** in DMSO-d_6 right after the preparation and after 72 h of incubation at 37°C

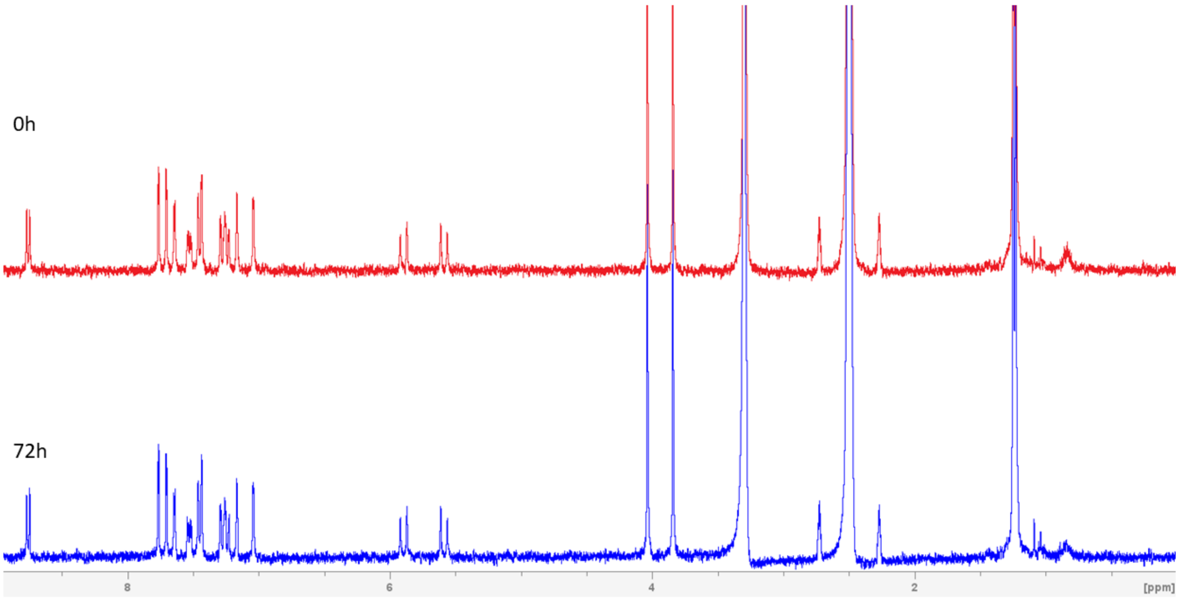

Figure S17: ^{1}H NMR spectrum of complex **BGC15b** in DMSO-d_6 right after the preparation and after 72 h of incubation at 37°C

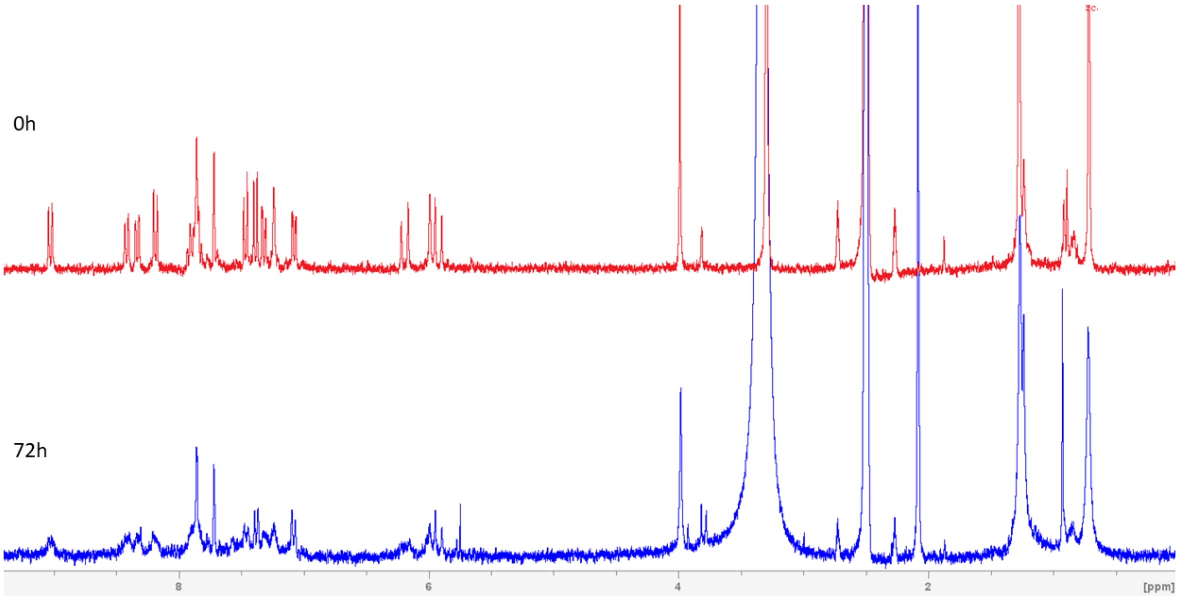

Figure S18: ^{1}H NMR spectrum of complex **BGC16b** in DMSO-d_6 right after the preparation and after 72 h of incubation at 37°C

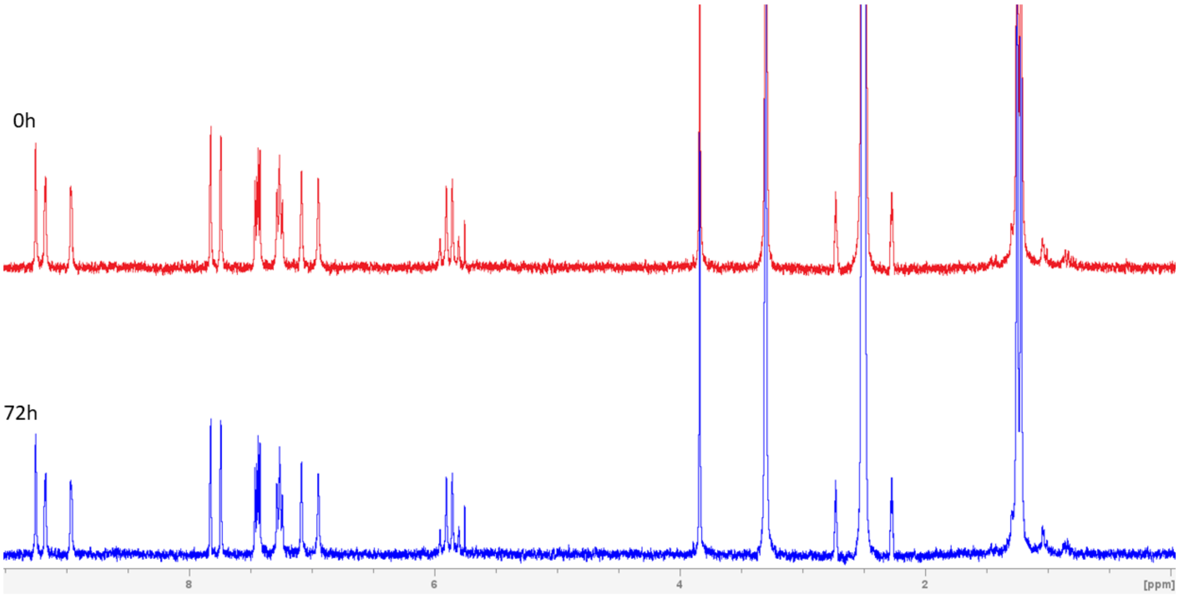

Figure S19: ^{1}H NMR spectrum of complex **BGC17b** in DMSO-d_6 right after the preparation and after 72 h of incubation at 37°C

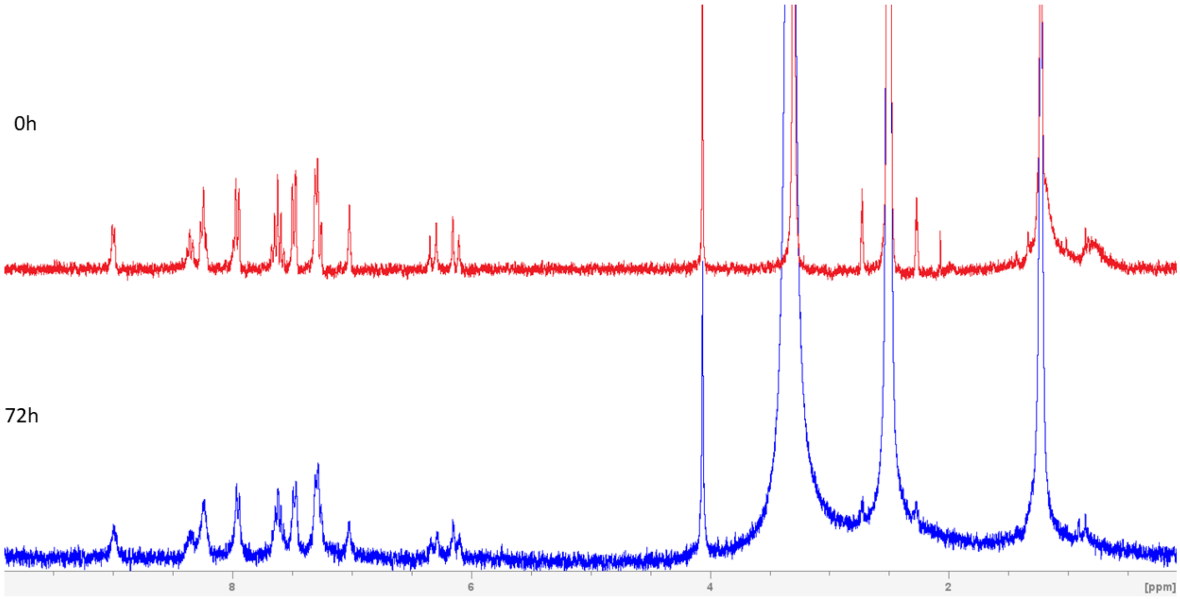

Figure S20: ^{1}H NMR spectrum of complex **BGC18b** in DMSO-d$_6$ right after dissolution and after 72 h of incubation at 37°C

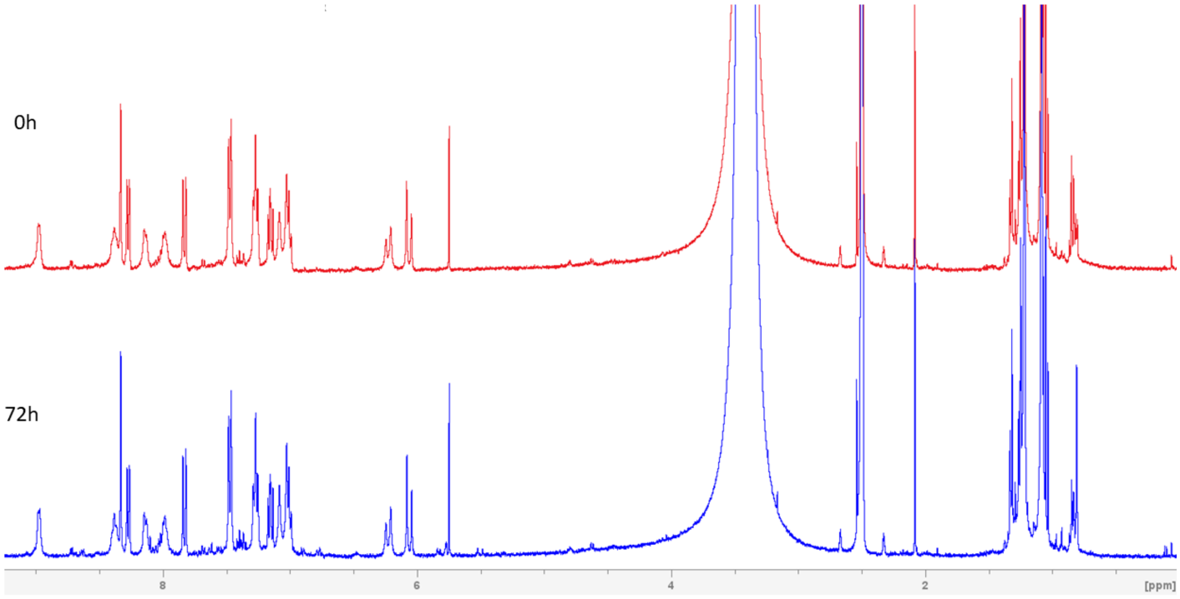

Figure S21: ^{1}H NMR spectrum of complex **BGC19b** in DMSO-d$_6$ right after dissolution and after 72 h of incubation at 37°C

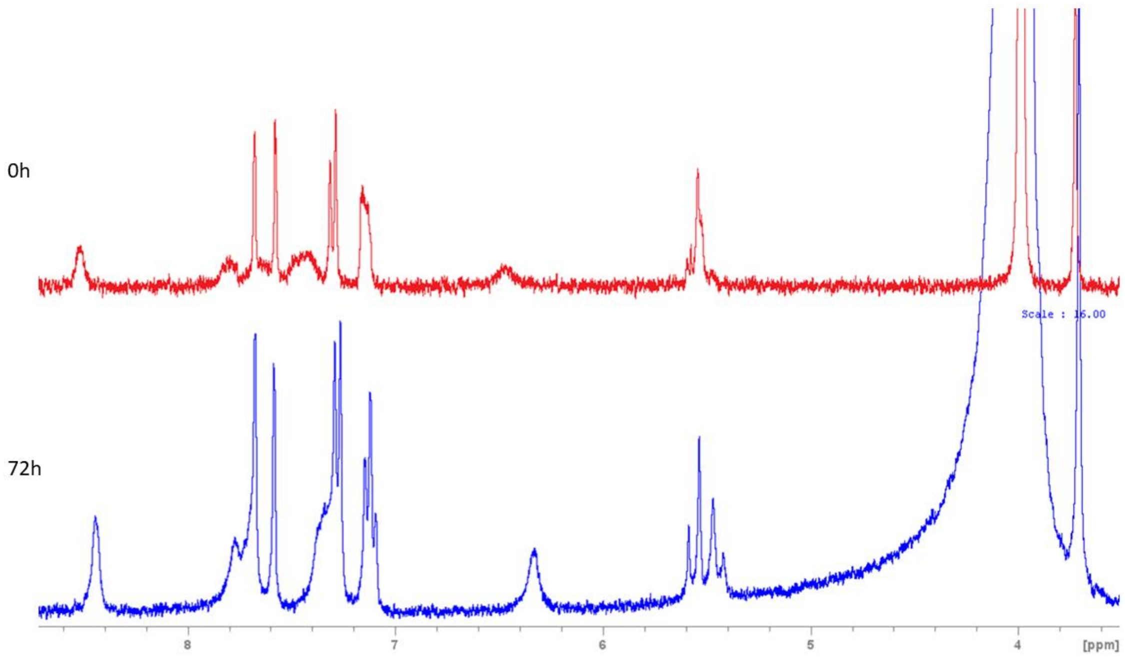

Figure S22: ^{1}H NMR spectrum of complex **BGC12a** in DMSO-d_6/D_2O (4:1 v/v) right after dissolution and after 72 h of incubation at 37°C

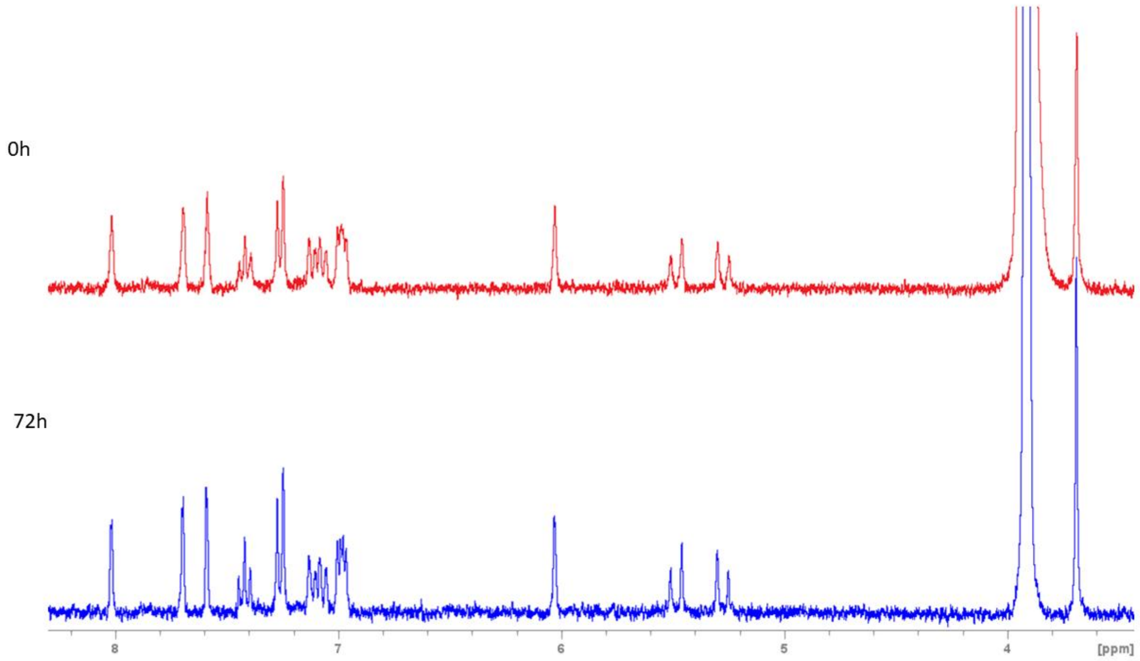

Figure S23: ^{1}H NMR spectrum of complex **BGC13a** in DMSO-d_6/D_2O (4:1 v/v) right after dissolution and after 72 h of incubation at 37°C

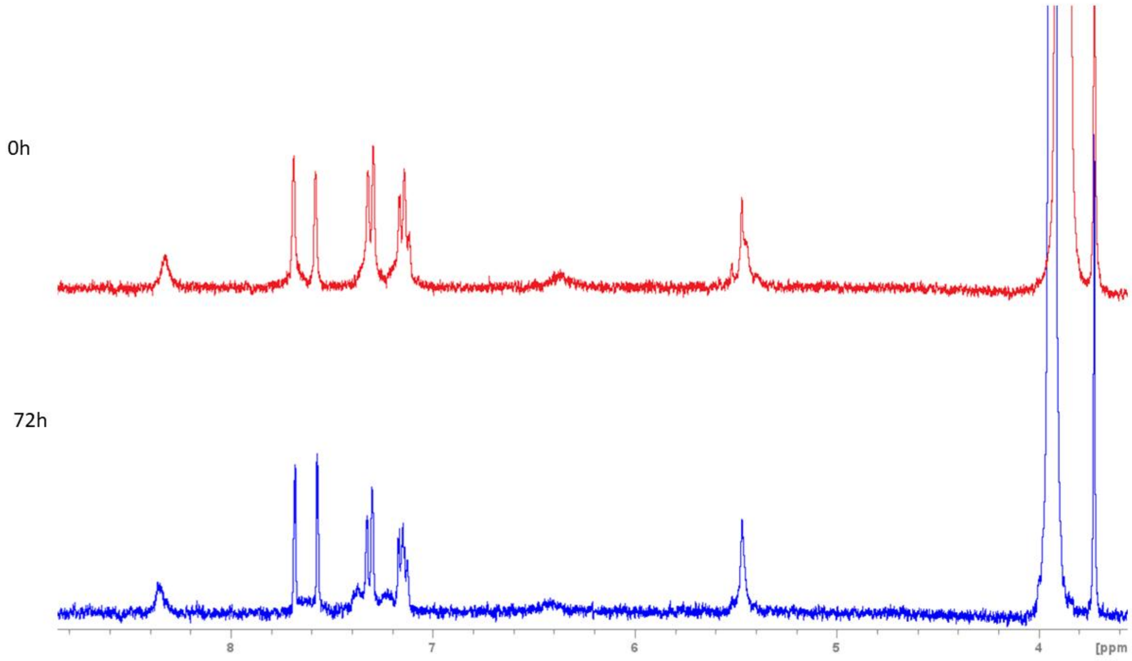

Figure S24: ^{1}H NMR spectrum of complex **BGC14a** in DMSO-d_6/D_2O (4:1 v/v) right after dissolution and after 72 h of incubation at 37°C

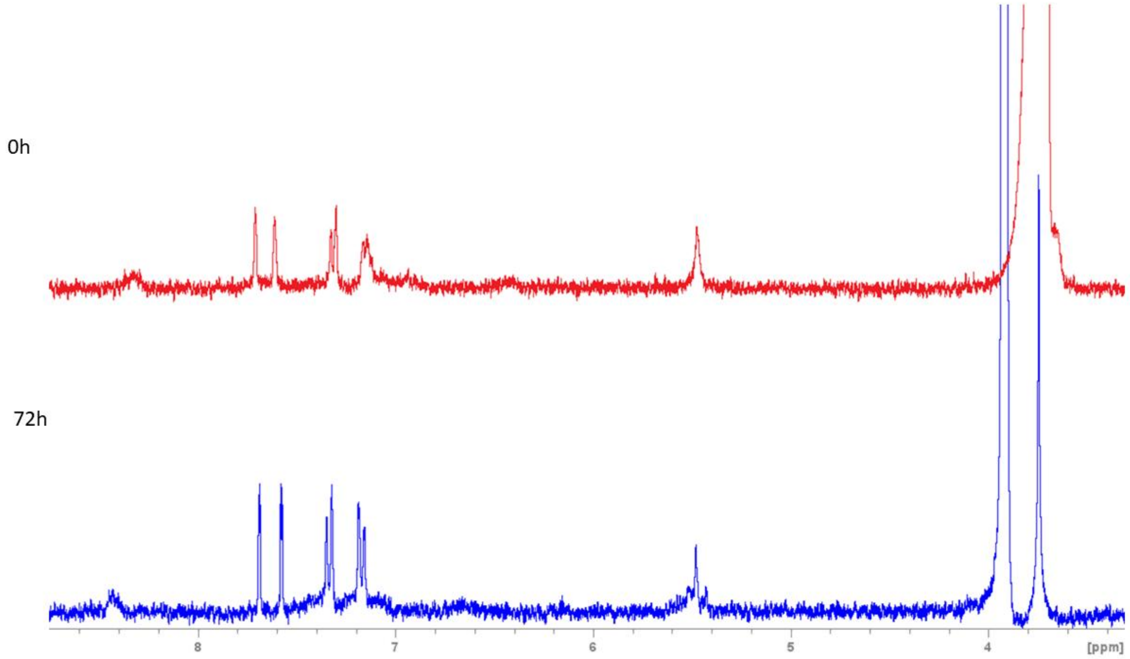

Figure S25: ^{1}H NMR spectrum of complex **BGC15a** in DMSO-d_6/D_2O (4:1 v/v) right after dissolution and after 72 h of incubation at 37°C

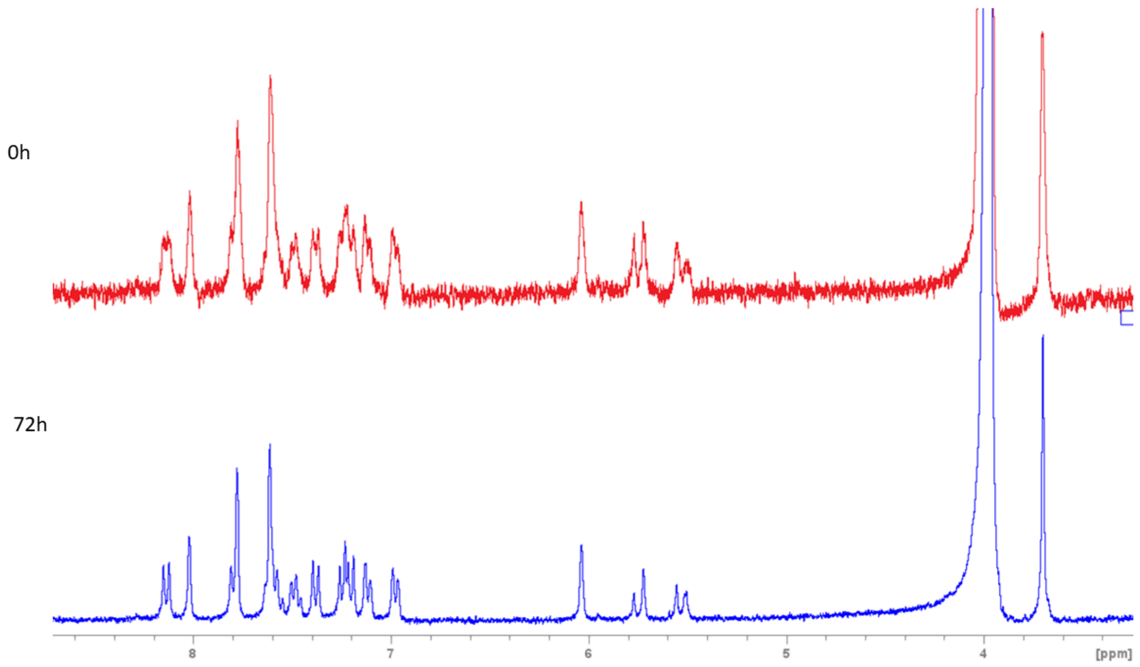

Figure S26: ^{1}H NMR spectrum of complex **BGC16a** in DMSO-d_6/D_2O (4:1 v/v) right after dissolution and after 72 h of incubation at 37°C

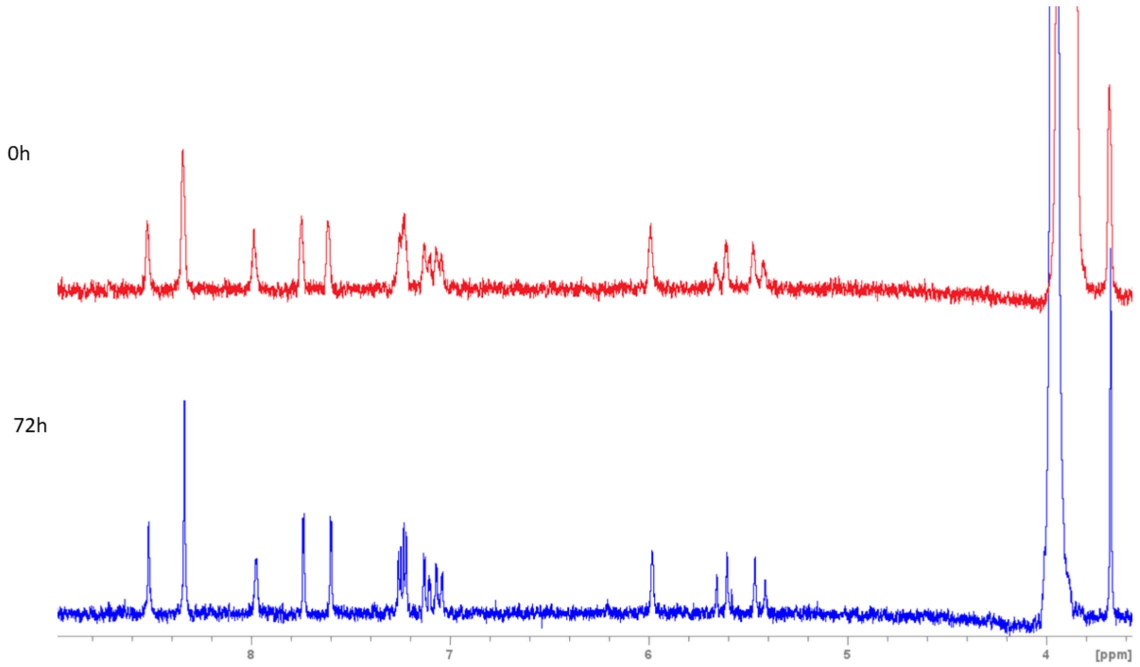

Figure S27: ^{1}H NMR spectrum of complex **BGC17a** in DMSO-d_6/D_2O (4:1 v/v) right after dissolution and after 72 h of incubation at 37°C

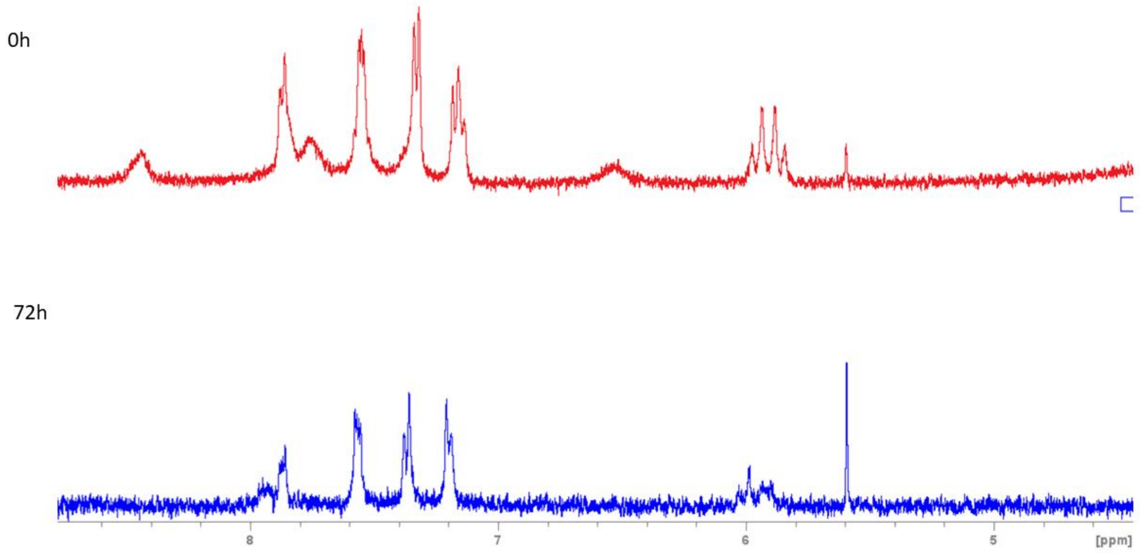

Figure S28: ^{1}H NMR spectrum of complex **BGC18a** in DMSO-d_6/D_2O (4:1 v/v) right after dissolution and after 72 h of incubation at 37°C

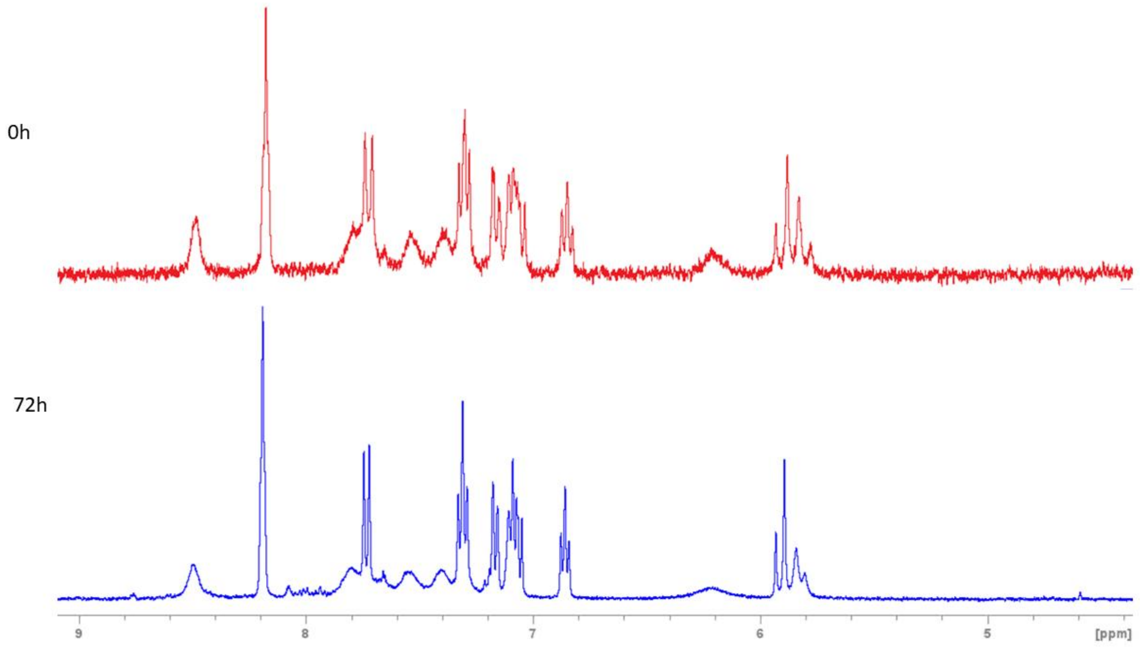

Figure S29: ^{1}H NMR spectrum of complex **BGC19a** in DMSO-d_6/D_2O (4:1 v/v) right after dissolution and after 72 h of incubation at 37°C

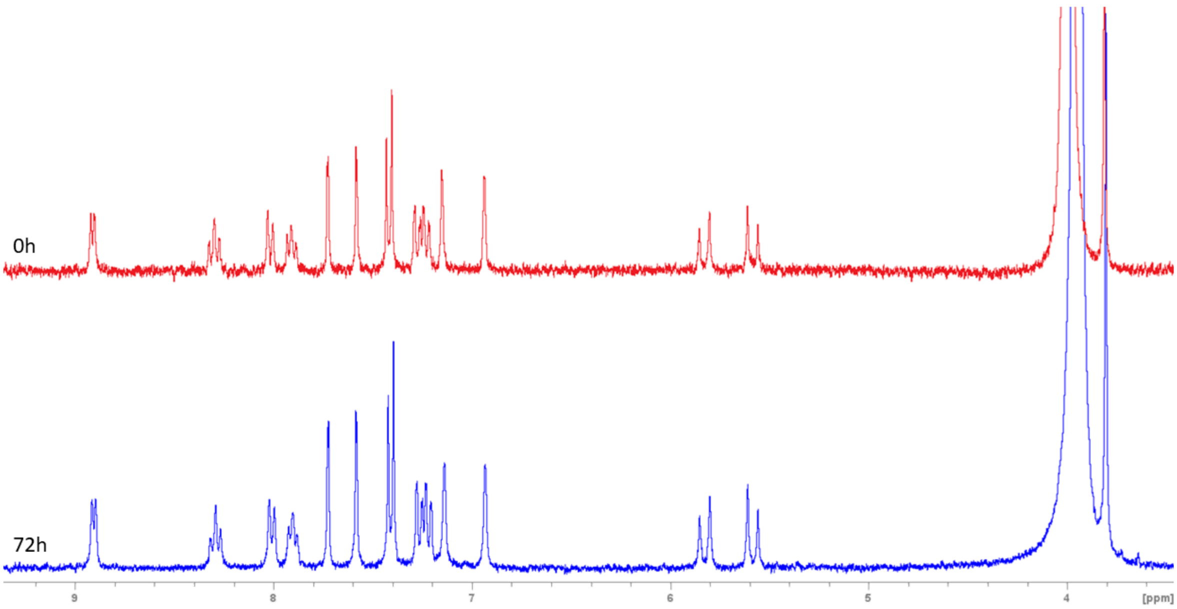

Figure S30: ^{1}H NMR spectrum of complex **BGC12b** in DMSO-d$_6$/D$_2$O (4:1 v/v) right after dissolution and after 72 h of incubation at 37°C

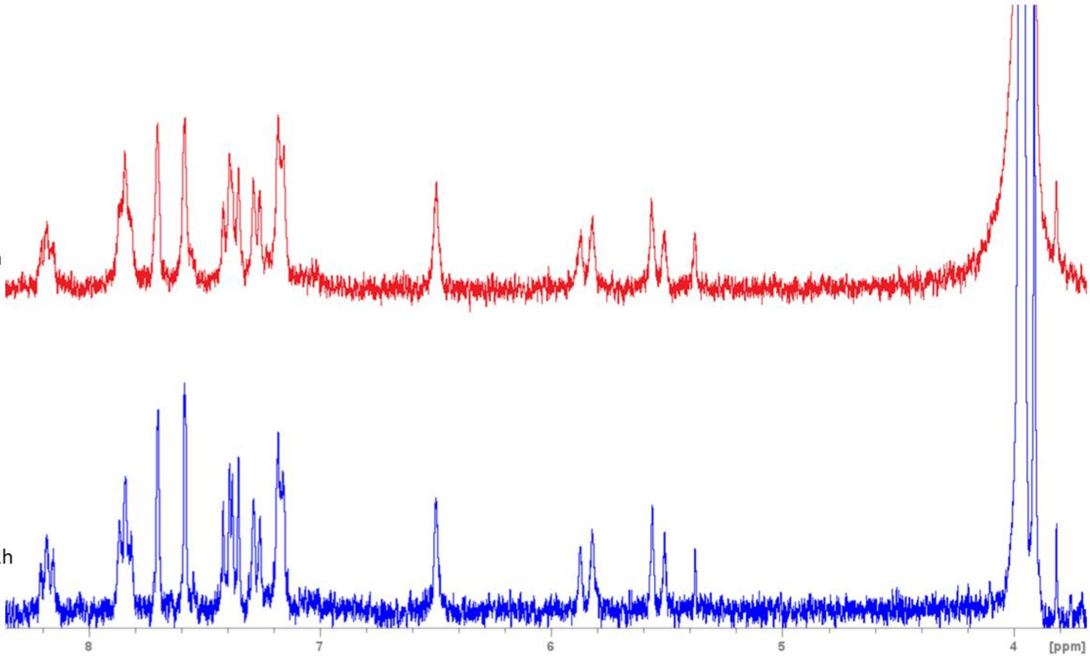

Figure S31: ^{1}H NMR spectrum of complex **BGC13b** in DMSO-d$_6$/D$_2$O (4:1 v/v) right after dissolution and after 72 h of incubation at 37°C

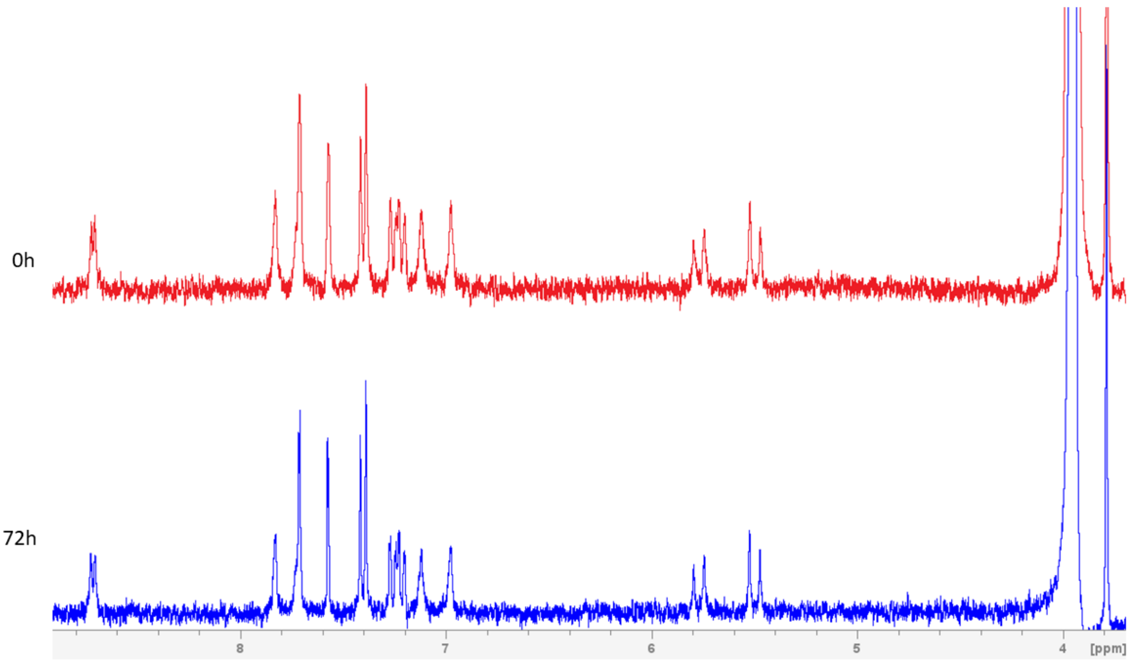

Figure S32: ^{1}H NMR spectrum of complex **BGC14b** in DMSO-d_6/D_2O (4:1 v/v) right after dissolution and after 72 h of incubation at 37°C

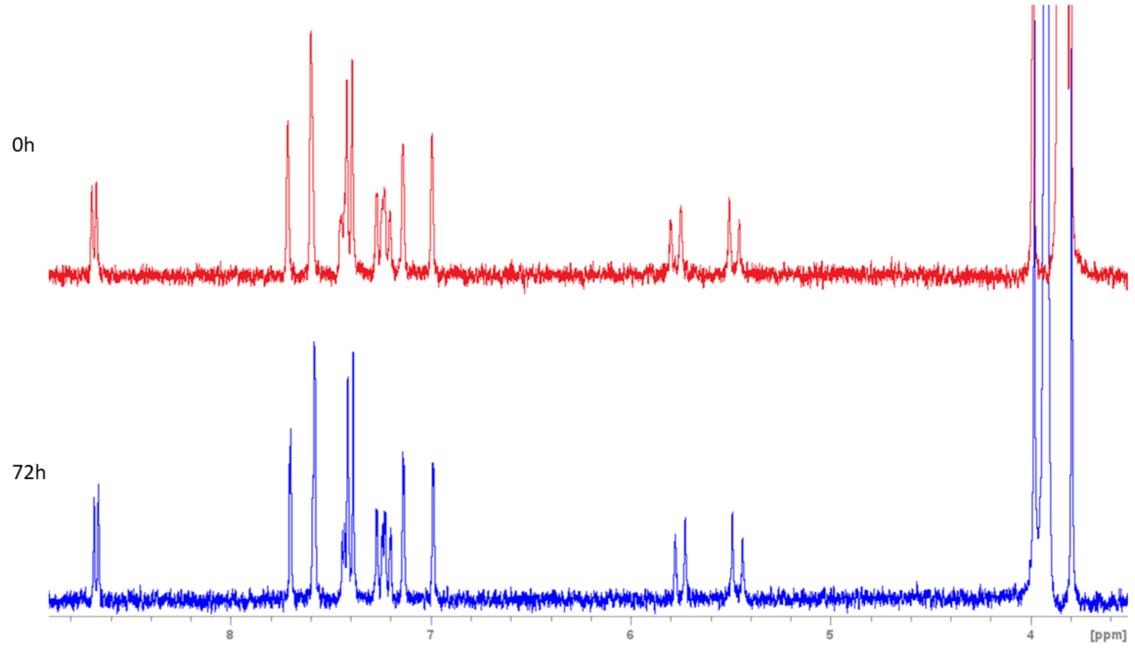

Figure S33: ^{1}H NMR spectrum of complex **BGC15b** in DMSO-d_6/D_2O (4:1 v/v) right after dissolution and after 72 h of incubation at 37°C

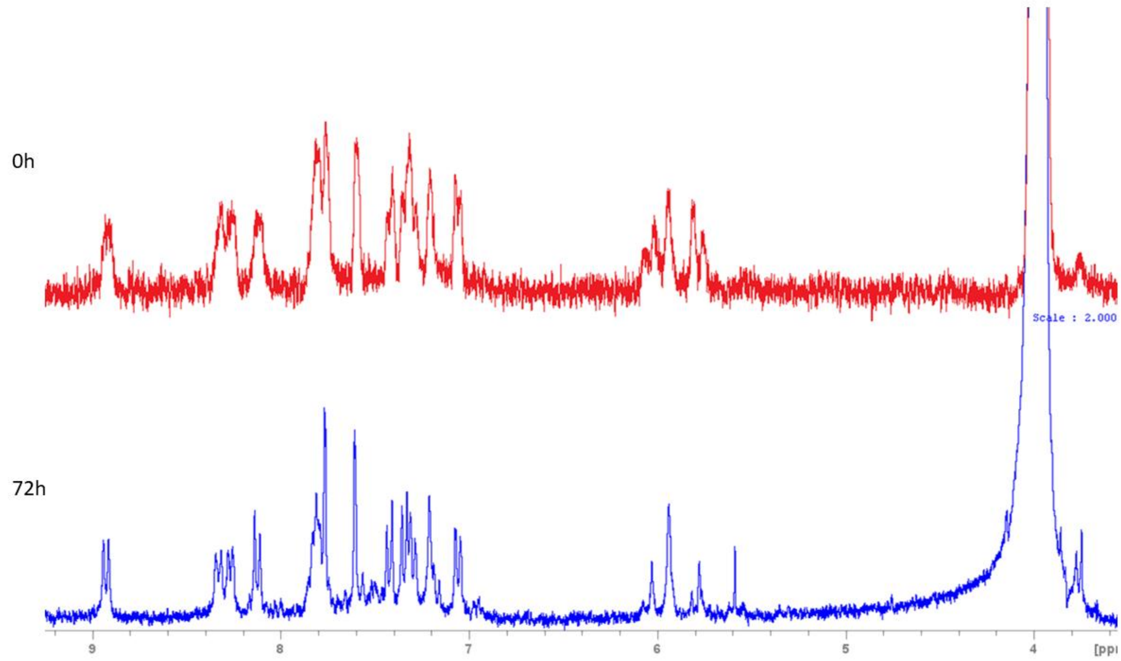

Figure S34: ^{1}H NMR spectrum of complex **BGC16b** in DMSO-d_6/D_2O (4:1 v/v) right after dissolution and after 72 h of incubation at 37°C

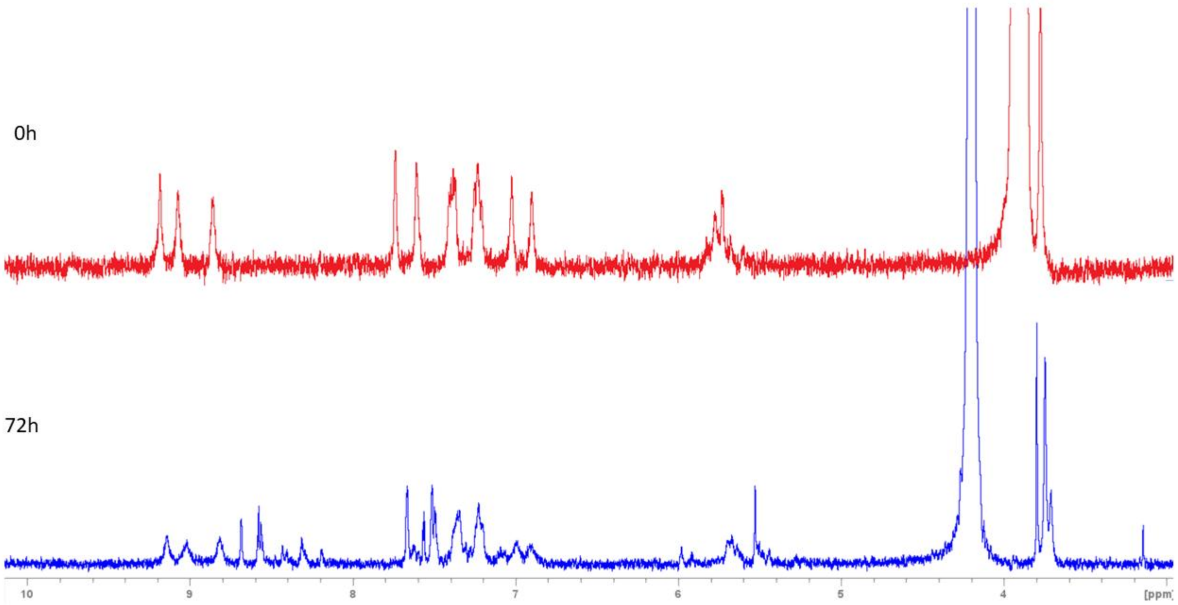

Figure S35: ^{1}H NMR spectrum of complex **BGC17b** in DMSO-d_6/D_2O (4:1 v/v) right after dissolution and after 72 h of incubation at 37°C

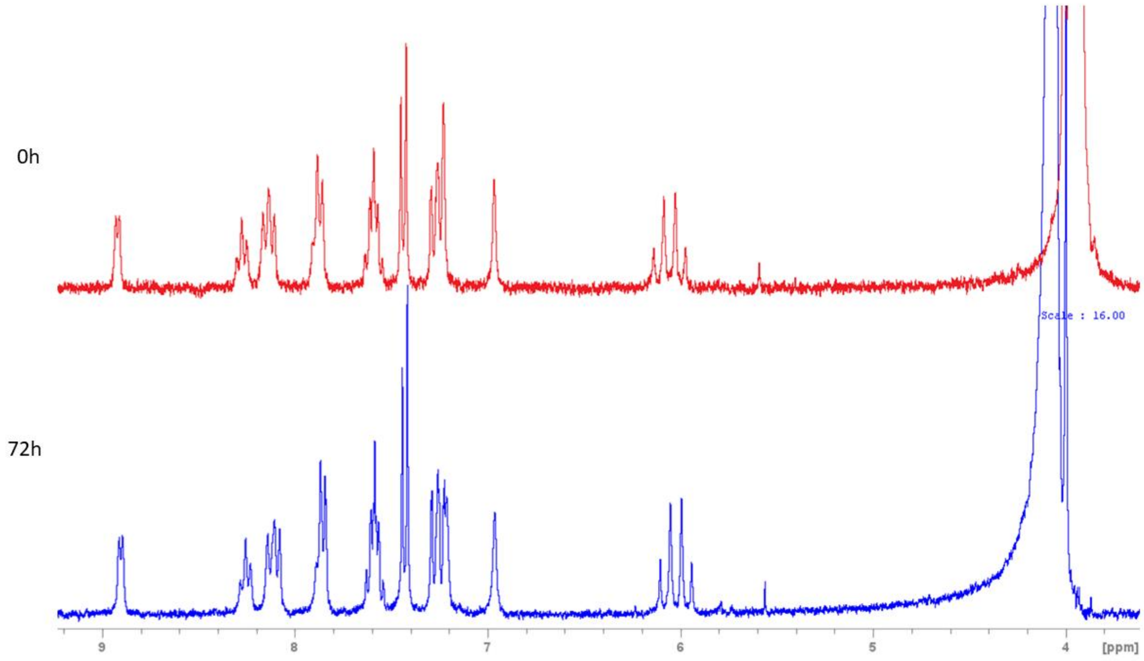

Figure S36: ^{1}H NMR spectrum of complex **BGC18b** in DMSO-d_6/D_2O (4:1 v/v) right after dissolution and after 72 h of incubation at 37°C

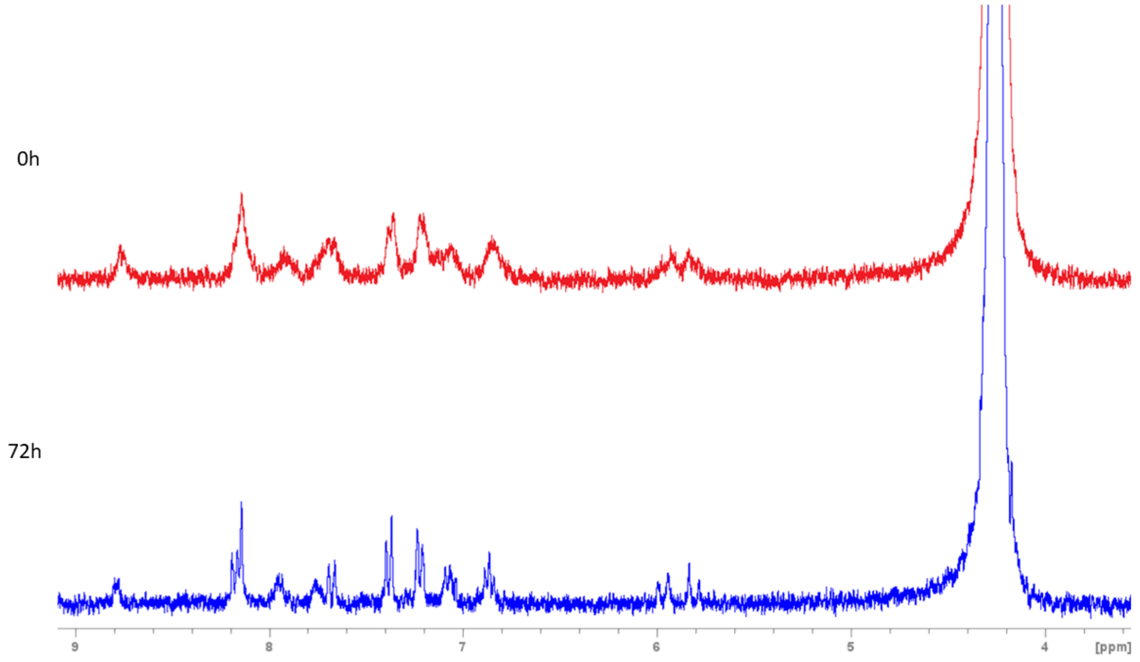

Figure S37: ^{1}H NMR spectrum of complex **BGC19b** in DMSO-d_6/D_2O (4:1 v/v) right after dissolution and after 72 h of incubation at 37°C

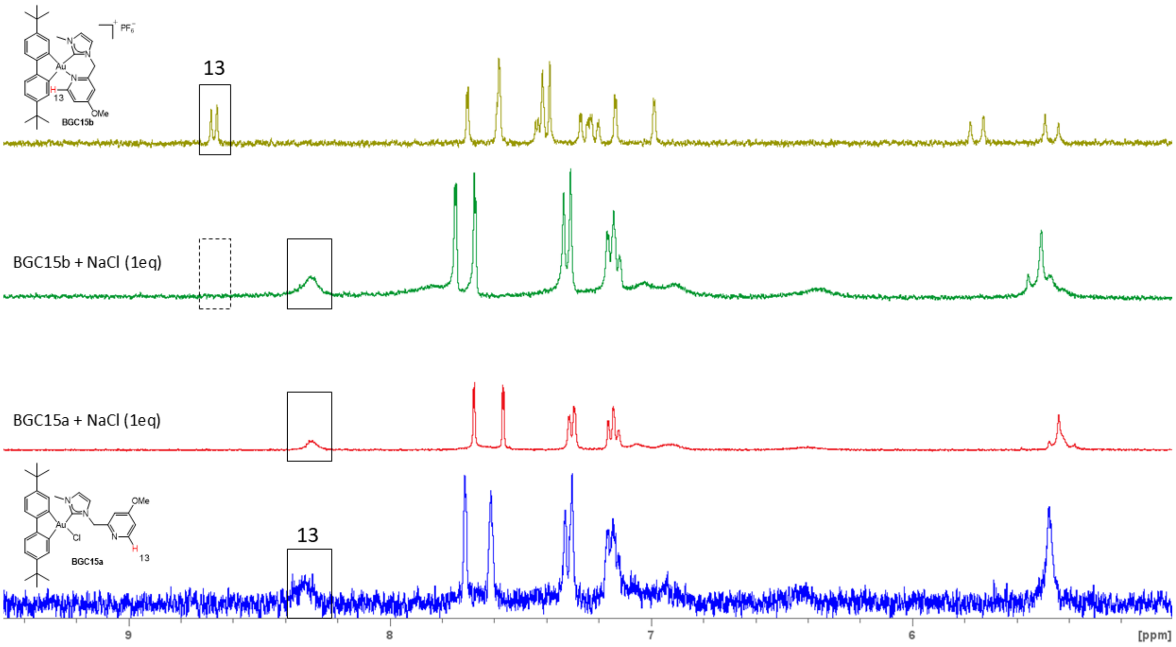

Figure S38: ^{1}H NMR spectrum of complex **BGC15a/b** in DMSO-d_6/D_2O (4:1 v/v) in presence or absence of NaCl (1 equiv.)

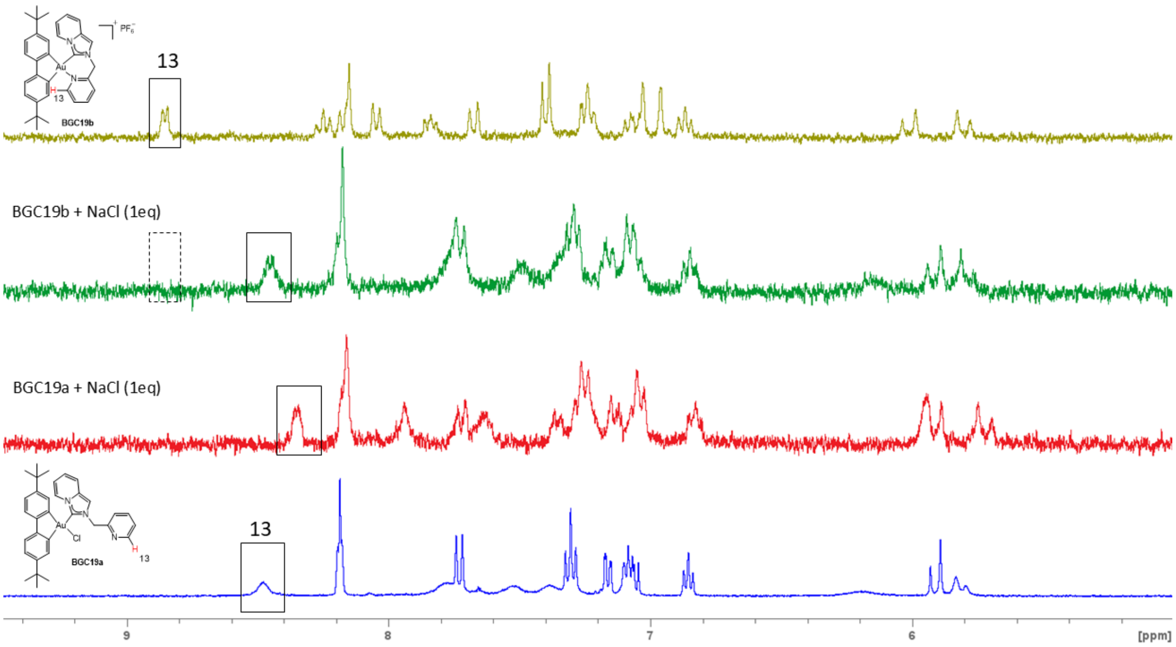

Figure S39: ^{1}H NMR spectrum of complex **BGC19a/b** in DMSO-d_6/D_2O (4:1 v/v) in presence or absence of NaCl (1 equiv.)

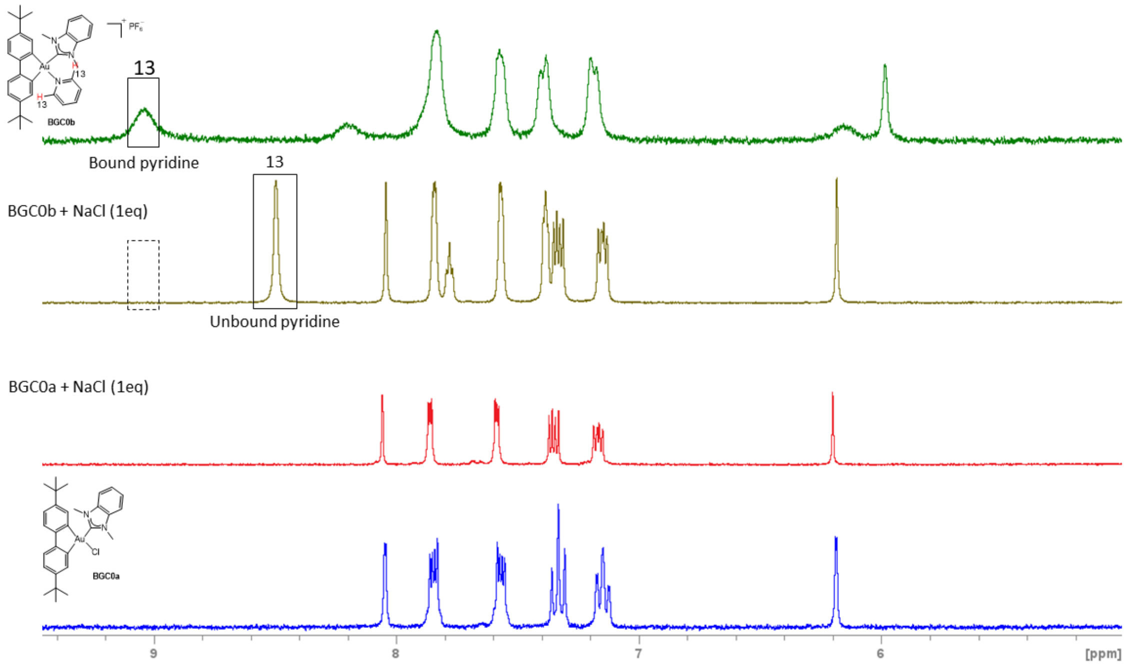

Figure S40: ^{1}H NMR spectrum of complex **BGC0a/b** in DMSO-d_6/D_2O (4:1 v/v) in presence or absence of NaCl (1 equiv.)

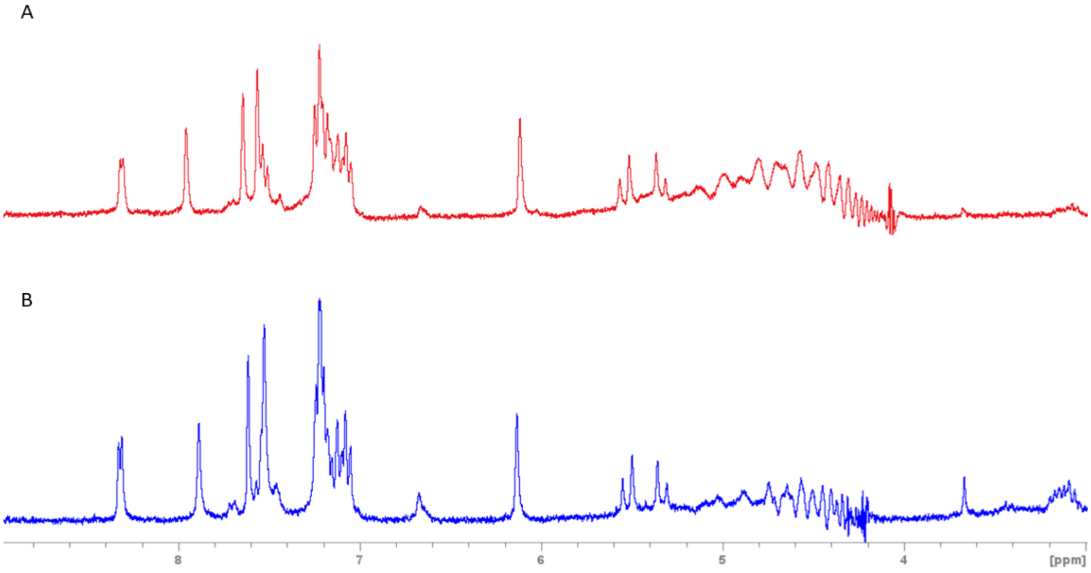

Figure S41: ^{1}H NMR spectra of (A) **BGC12a** and (B) **BGC12b** in DMSO-d_6/DMEM 3:1 mix using CPMGR-ESPG1D water suppression sequence (carrier frequency O1P is adjusted to the maximum of the water peak, Hahn echo delay D20 is left null)

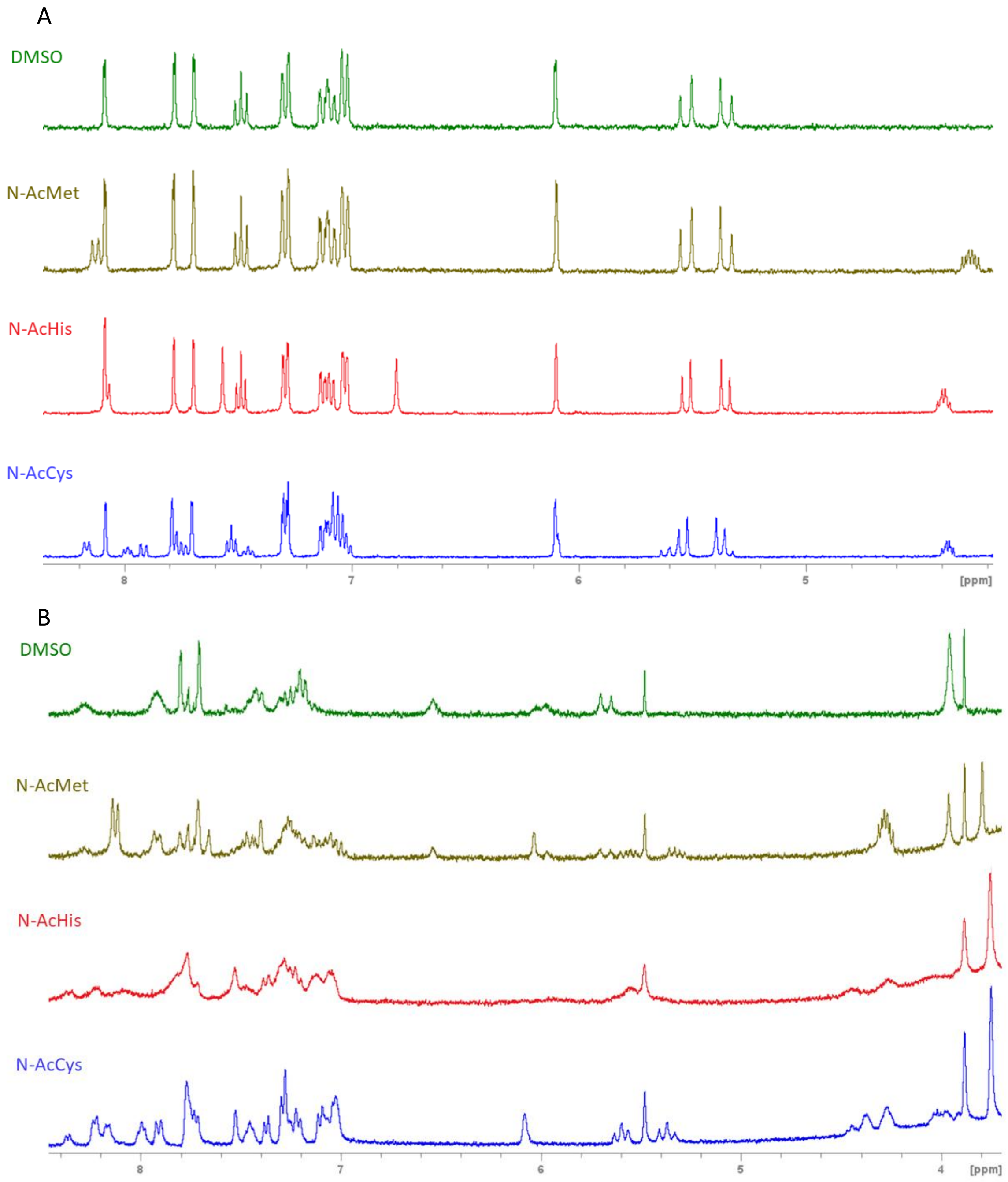

Figure S42: ^{1}H NMR spectra recorded at 5 mM in DMSO-d_6 immediately after mixing of A) **BGC13a** alone and reacted with 1 equiv. of N-Ac-methionine, N-Ac-histidine and N-Ac-cysteine; B) **BGC13b** alone and reacted with 1 equiv. of N-Ac-methionine, N-Ac-histidine and N-Ac-cysteine.

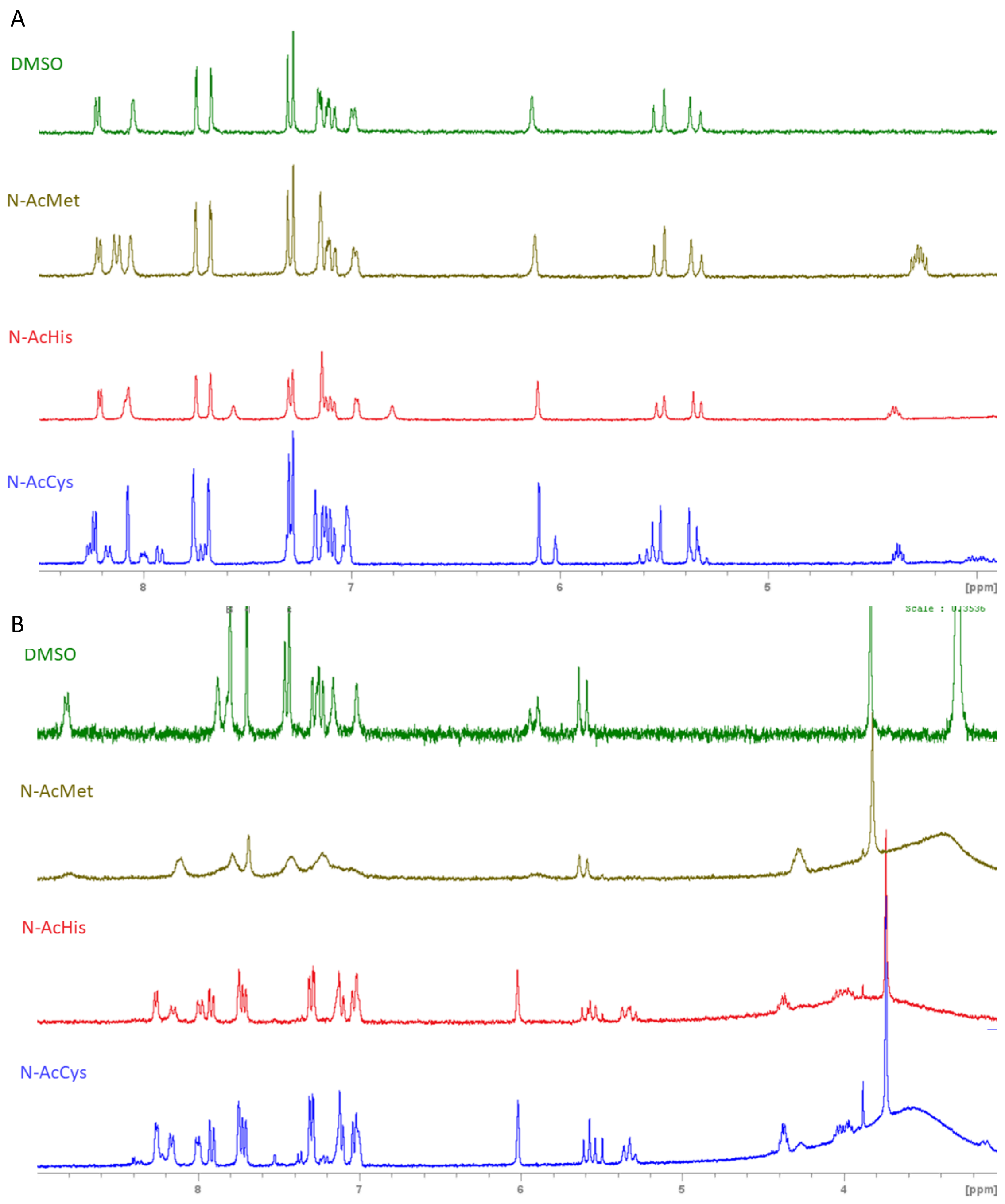

Figure S43: ^{1}H NMR spectra recorded at 5 mM in DMSO-d_6 immediately after mixing of A) **BGC14a** alone and reacted with 1 equiv. of N-Ac-methionine, N-Ac-histidine and N-Ac-cysteine; B) **BGC14b** alone and reacted with 1 equiv. of N-Ac-methionine, N-Ac-histidine and N-Ac-cysteine.

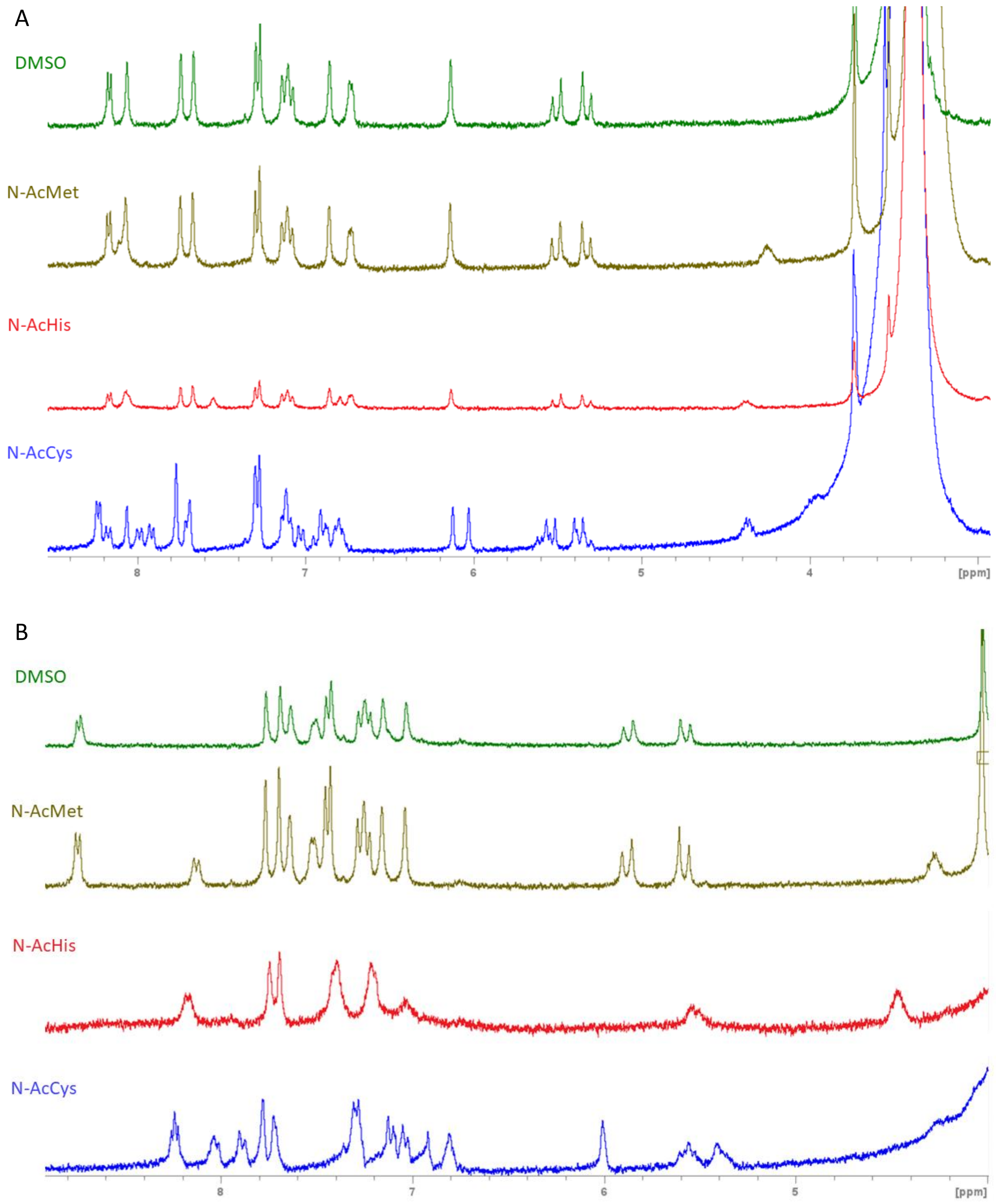

Figure S44: ^{1}H NMR spectra recorded at 5 mM in DMSO-d$_6$ immediately after mixing of A) **BGC15a** alone and reacted with 1 equiv. of N-Ac-methionine, N-Ac-histidine and N-Ac-cysteine; B) **BGC15b** alone and reacted with 1 equiv. of N-Ac-methionine, N-Ac-histidine and N-Ac-cysteine.

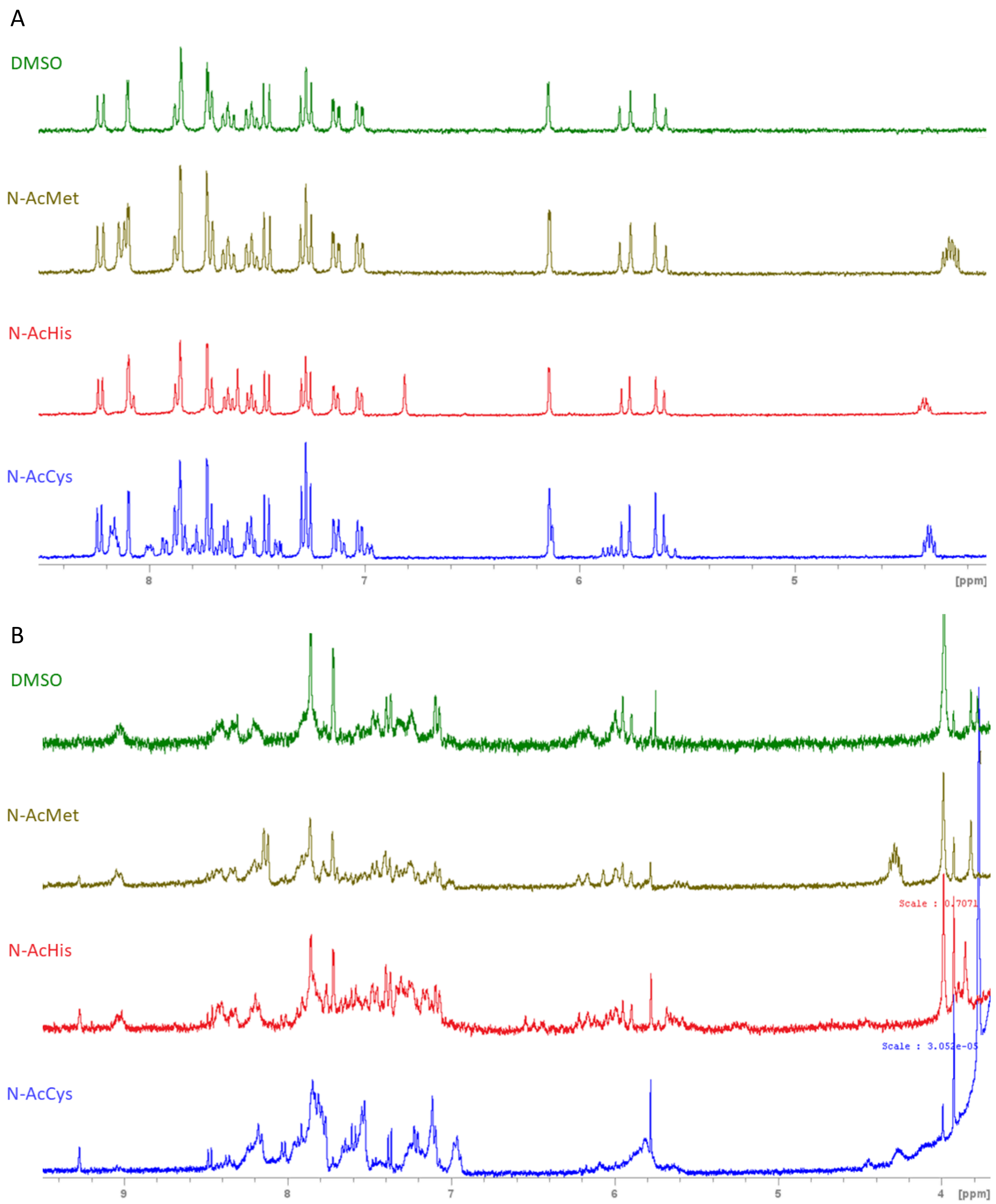

Figure S45: ^{1}H NMR spectra recorded at 5 mM in DMSO-d_6 immediately after mixing of A) **BGC16a** alone and reacted with 1 equiv. of N-Ac-methionine, N-Ac-histidine and N-Ac-cysteine; B) **BGC16b** alone and reacted with 1 equiv. of N-Ac-methionine, N-Ac-histidine and N-Ac-cysteine.

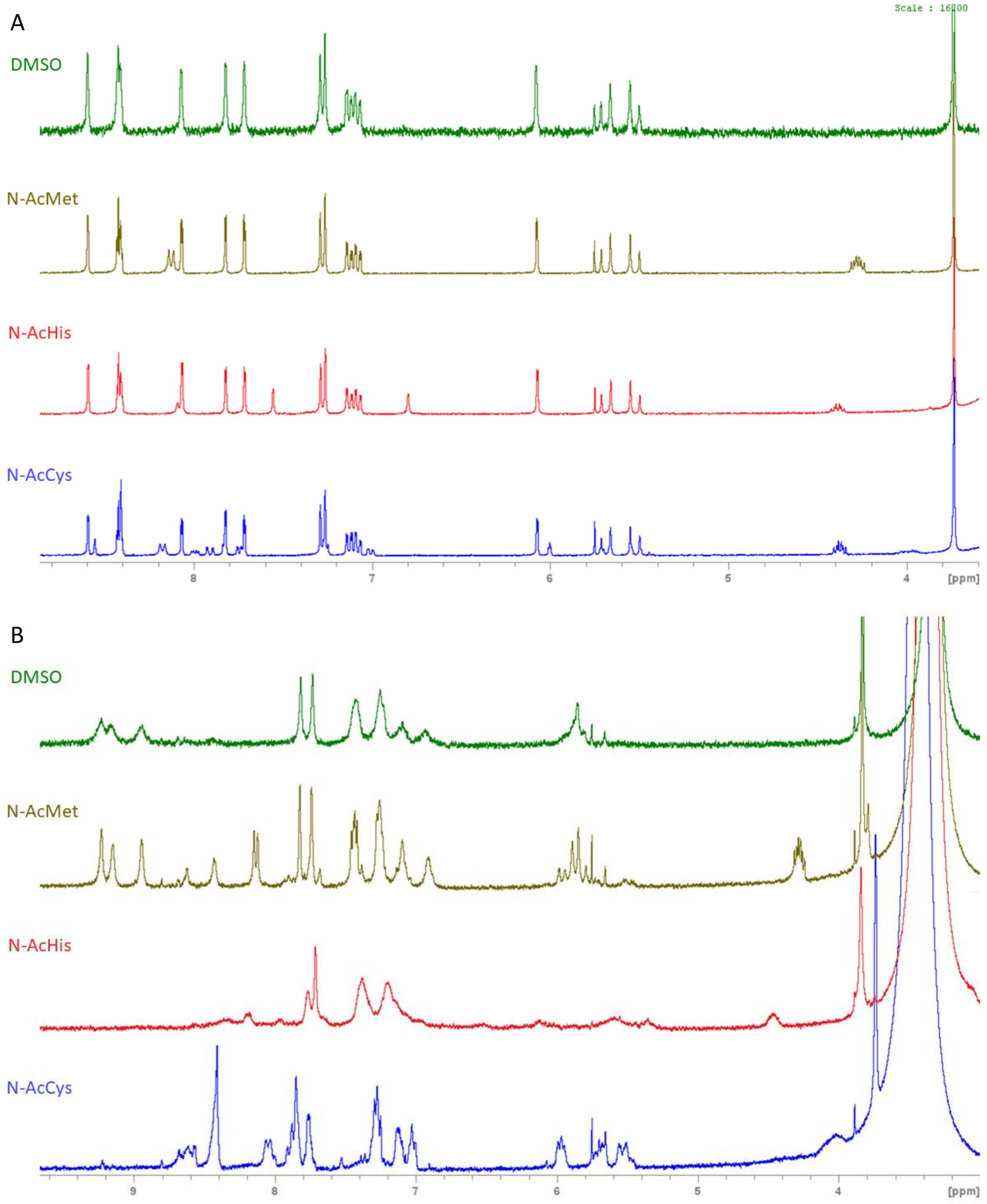

Figure S46: ^{1}H NMR spectra recorded at 5 mM in DMSO-d_6 immediately after mixing of A) **BGC17a** alone and reacted with 1 equiv. of N-Ac-methionine, N-Ac-histidine and N-Ac-cysteine; B) **BGC17b** alone and reacted with 1 equiv. of N-Ac-methionine, N-Ac-histidine and N-Ac-cysteine.

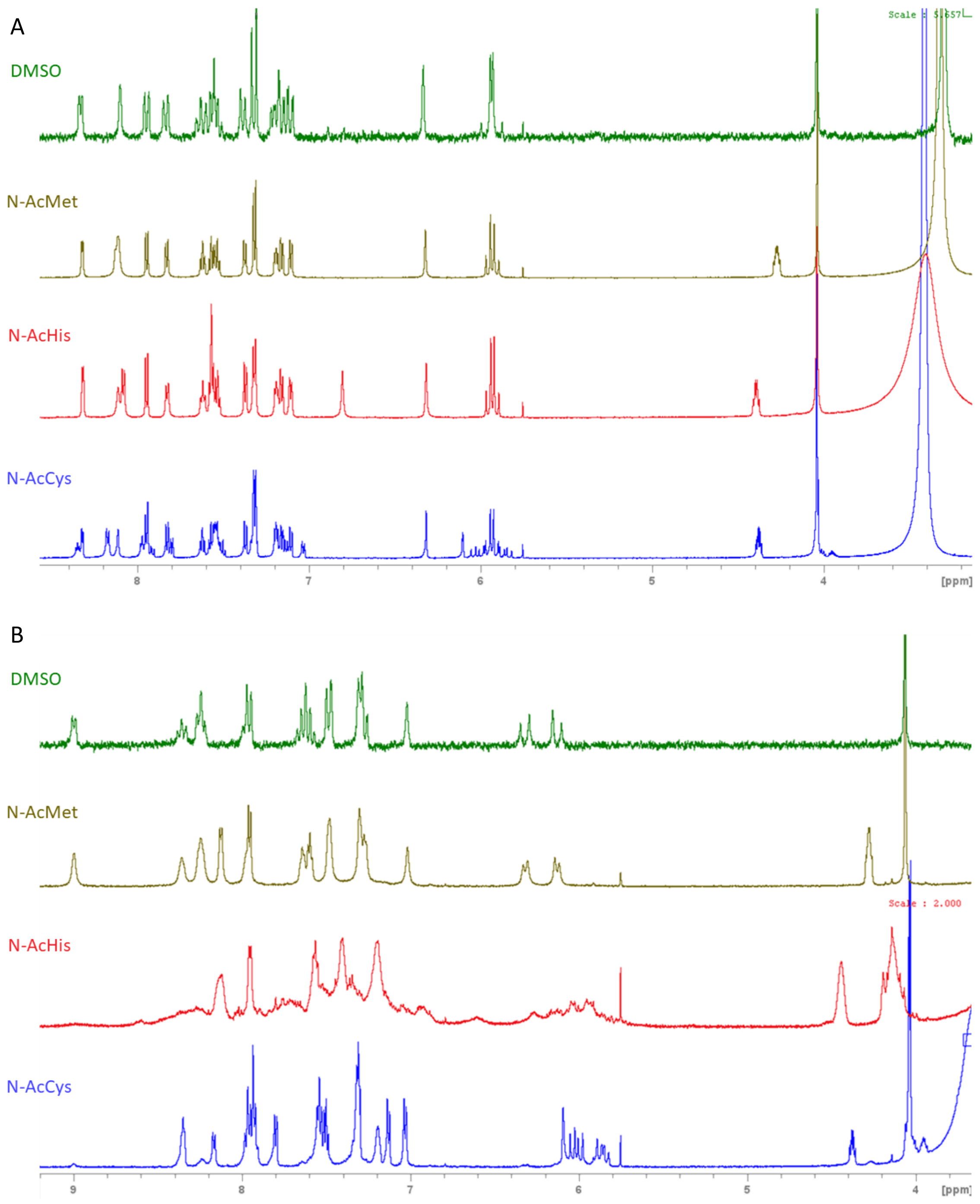

Figure S47: ^{1}H NMR spectra recorded at 5 mM in DMSO-d_6 immediately after mixing of A) **BGC18a** alone and reacted with 1 equiv. of N-Ac-methionine, N-Ac-histidine and N-Ac-cysteine; B) **BGC18b** alone and reacted with 1 equiv. of N-Ac-methionine, N-Ac-histidine and N-Ac-cysteine.

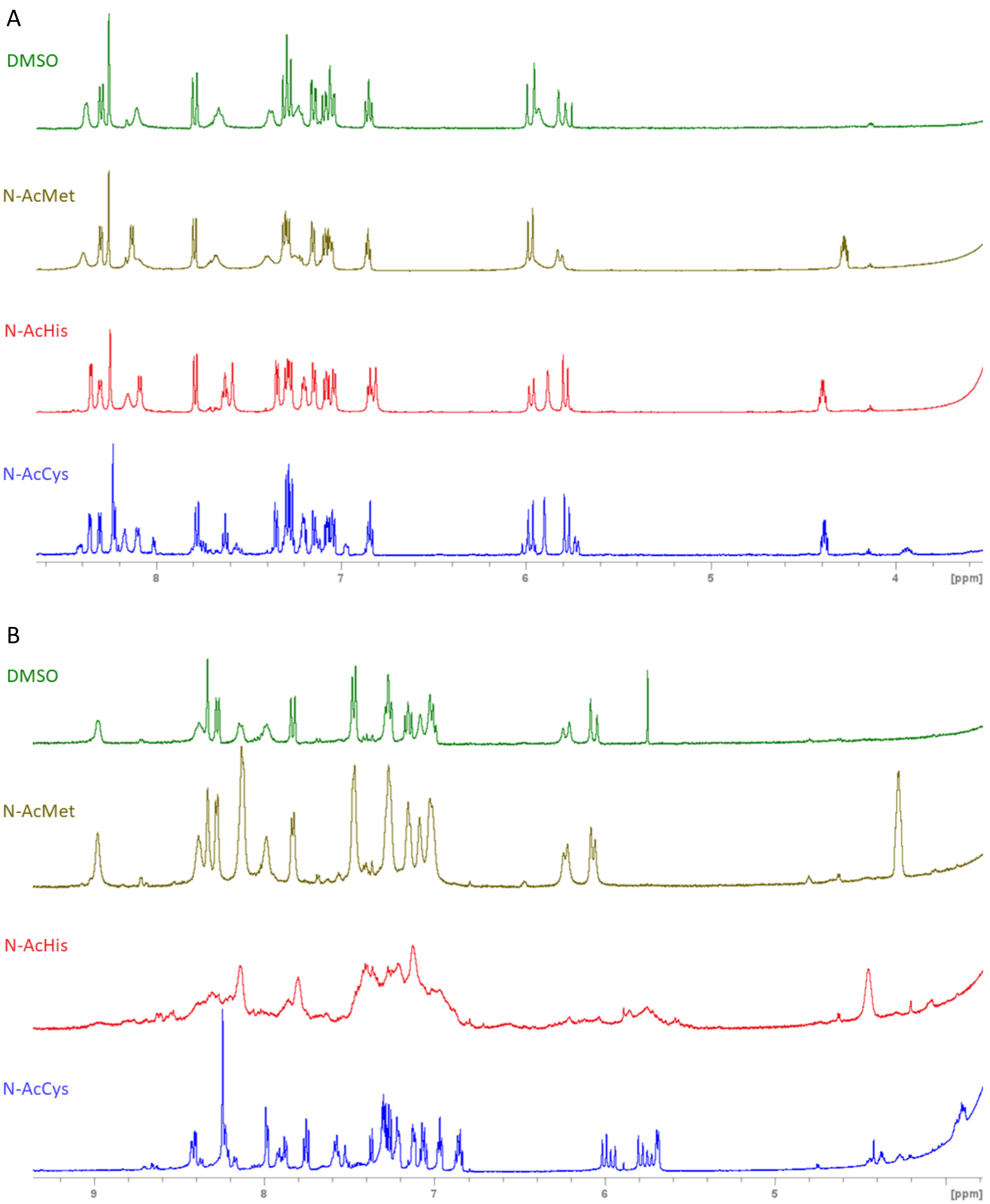

Figure S48: ^{1}H NMR spectra recorded at 5 mM in DMSO-d_6 immediately after mixing of A) **BGC19a** alone and reacted with 1 equiv. of N-Ac-methionine, N-Ac-histidine and N-Ac-cysteine; B) **BGC19b** alone and reacted with 1 equiv. of N-Ac-methionine, N-Ac-histidine and N-Ac-cysteine.

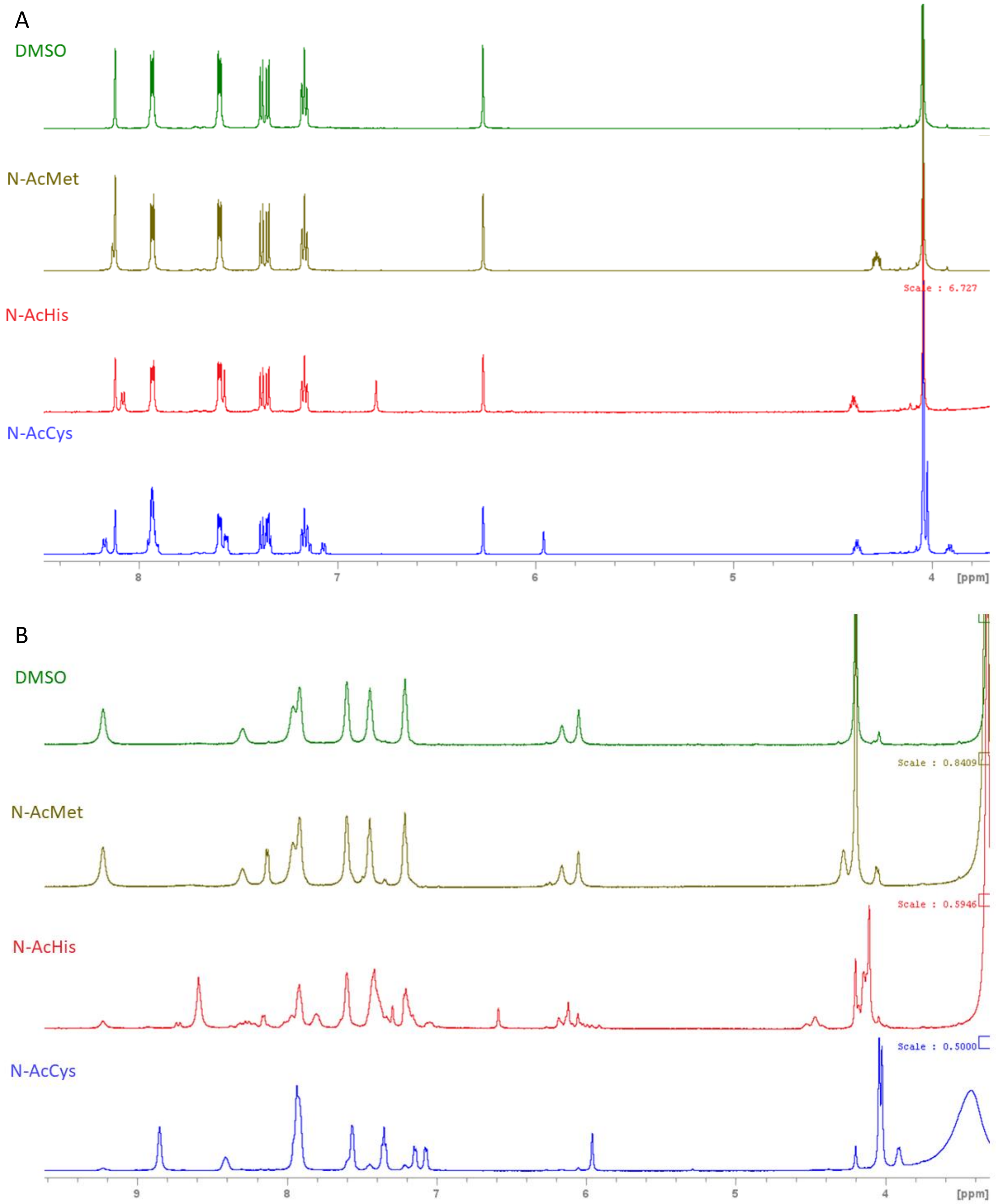

Figure S49: ^{1}H NMR spectra recorded at 5 mM in DMSO-d$_6$ immediately after mixing of A) **BGC0a** alone and reacted with 1 equiv. of N-Ac-methionine, N-Ac-histidine and N-Ac-cysteine; B) **BGC0b** alone and reacted with 1 equiv. of N-Ac-methionine, N-Ac-histidine and N-Ac-cysteine.

A

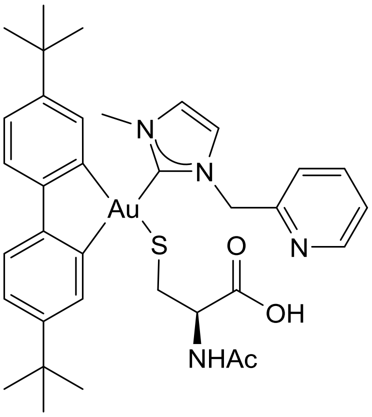

B

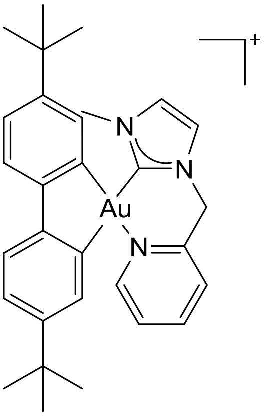

(A) HRMS (ESI+) m/z: $[M+Na]^+$ Calcd for $C_{35}H_{43}O_3N_4SAuNa$ 819.2614. Found 819.26; (Error: -1.7 ppm).

A

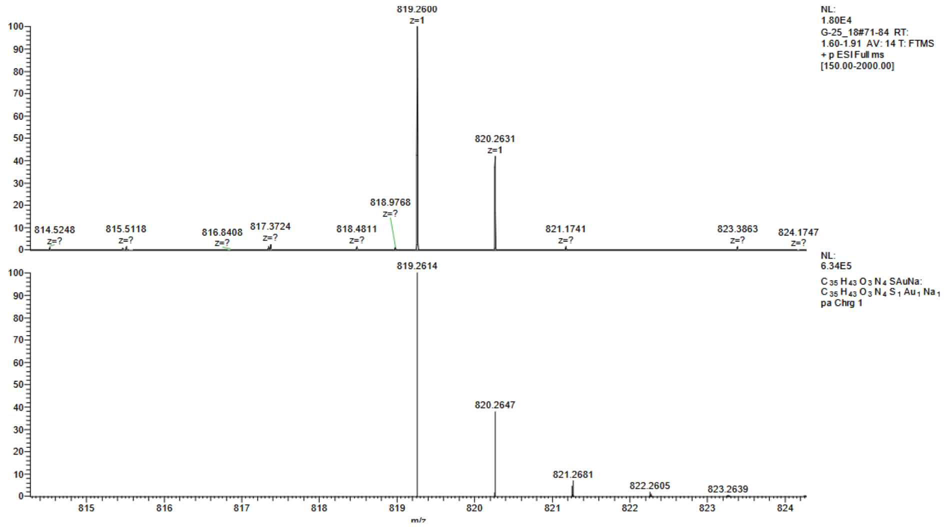

(B) HRMS (ESI) m/z: $[M-PF_6]^+$ Calcd for $C_{30}H_{35}AuN_3^+$ 634.2491. Found 634.2484; (Error: -0.8 ppm).

B

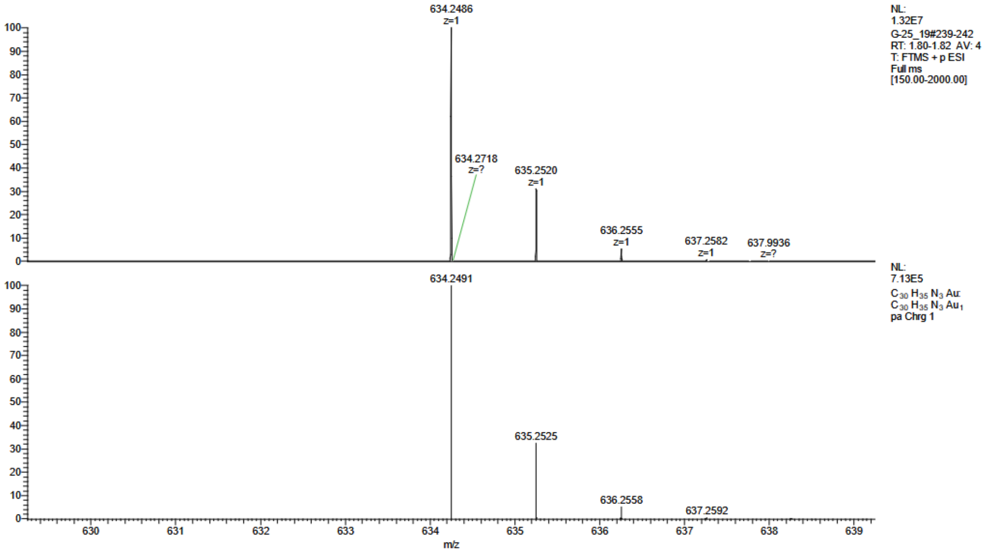

Fig S50: Structures of the products and HRMS experimental (up) and computed (down) spectra from the reaction of (A) **BGC12b** and N-acetylcysteine (1 equiv.) and (B) **BGC12b** and N-acetylhistidine (1 equiv.)

Table S4: ^{1}H NMR conversion rates of the reaction of **BGC** complex with 1 equivalent of N-Acetyl amino acid straight after mixing.

Complex	NMR Conversion (%)					
	Neutral (a form)			Cationic (b form)		
	N-AcCys	N-AcHis	N-AcMet	N-AcCys	N-AcHis	N-AcMet
**BGC12**	32	0	0	100	92	0
**BGC13**	30	0	0	100	100	0
**BGC14**	27	0	0	100	100	0
**BGC15**	29	0	0	100	100	0
**BGC16**	33	0	0	100	62	0
**BGC17**	27	0	0	100	100	0
**BGC18**	37	0	0	100	100	0
**BGC19**	28	0	0	100	100	0
**BGC0**	31	0	0	95	87	0

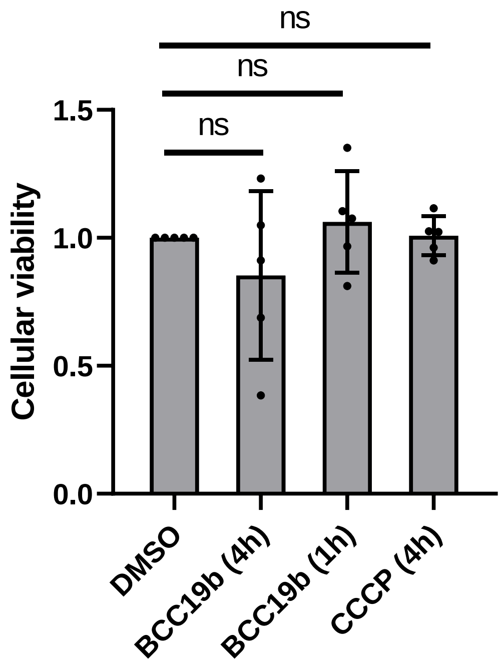

Figure S51: Cellular viability of HeLa cells treated with DMSO (4 h, normalization reference), **BGC19b** (10 µM, 4 h and 1 h) and **CCCP** (10 µM, 4 h). Viability measured using resazurin assay as described in the experimental part.

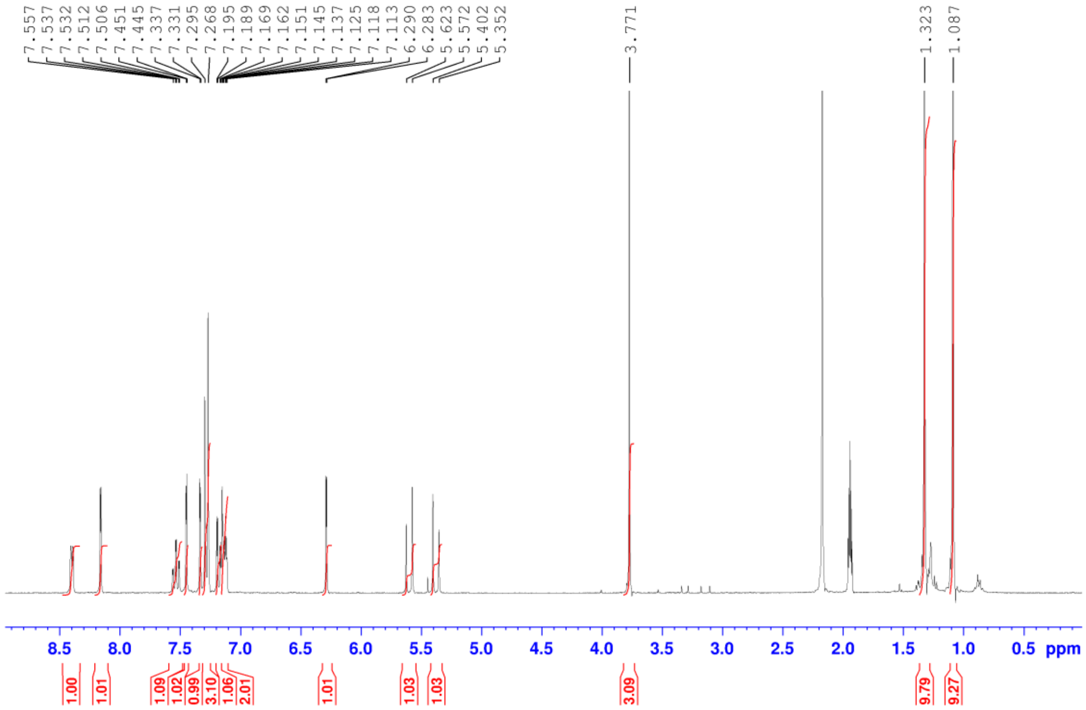

Figure S52: ^{1}H NMR of complex **BGC12a** in CD_3CN, recorded at 300 MHz and 300 K

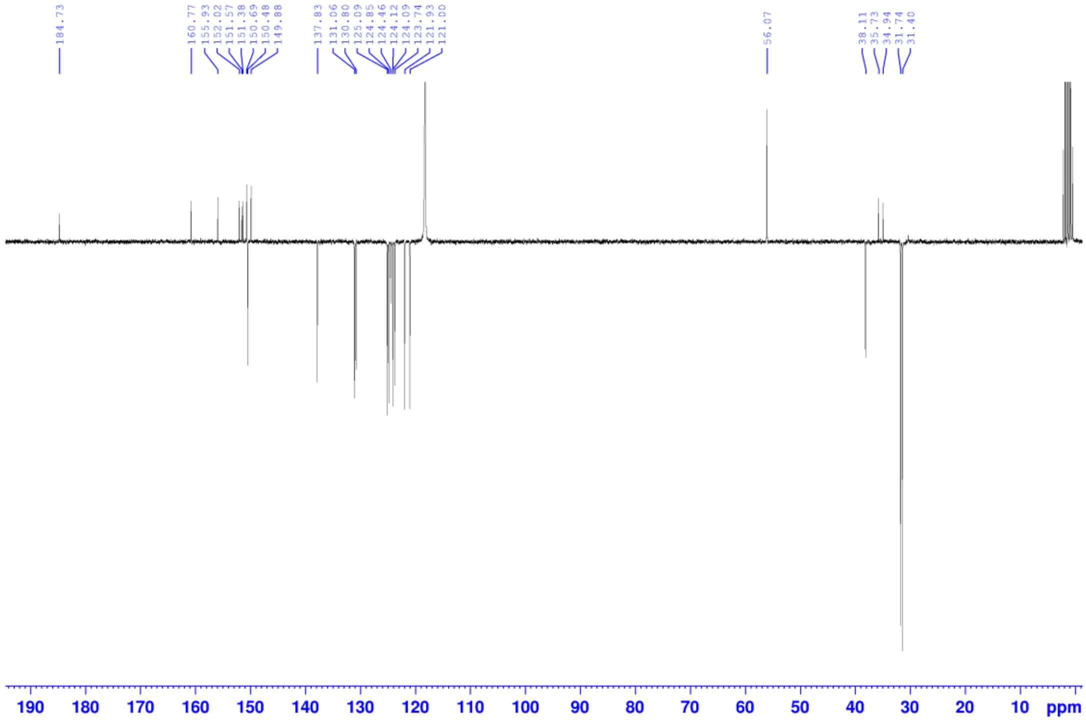

Figure S53: $^{13}C\{^{1}H\}$ JMod NMR of complex **BGC12a** in CD_3CN, recorded at 75 MHz and 300 K

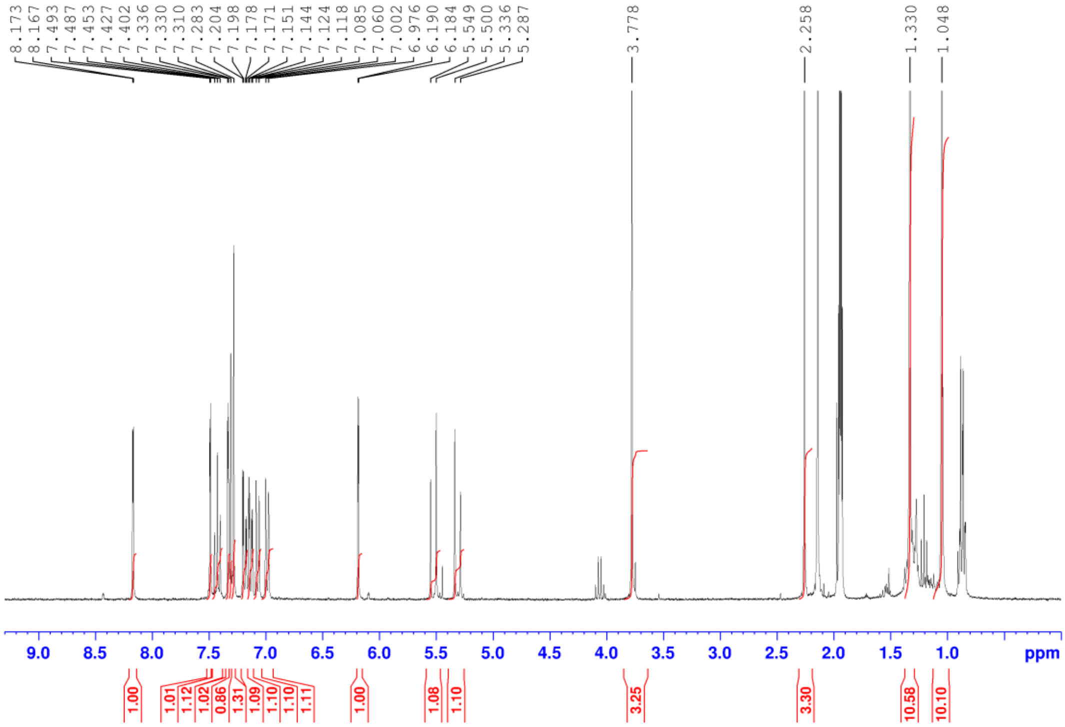

Figure S54: ^{1}H NMR of complex **BGC13a** in CD_3CN, recorded at 300 MHz and 300 K

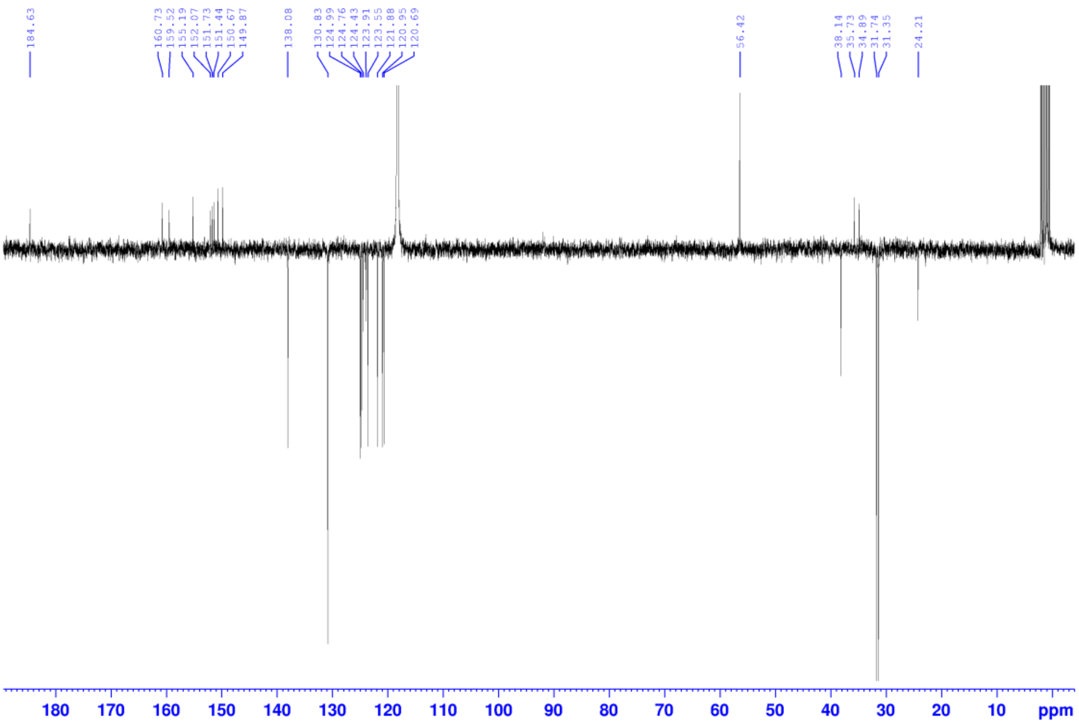

Figure S55: $^{13}C\{^{1}H\}$ JMod NMR of complex **BGC13a** in CD_3CN, recorded at 75 MHz and 300 K

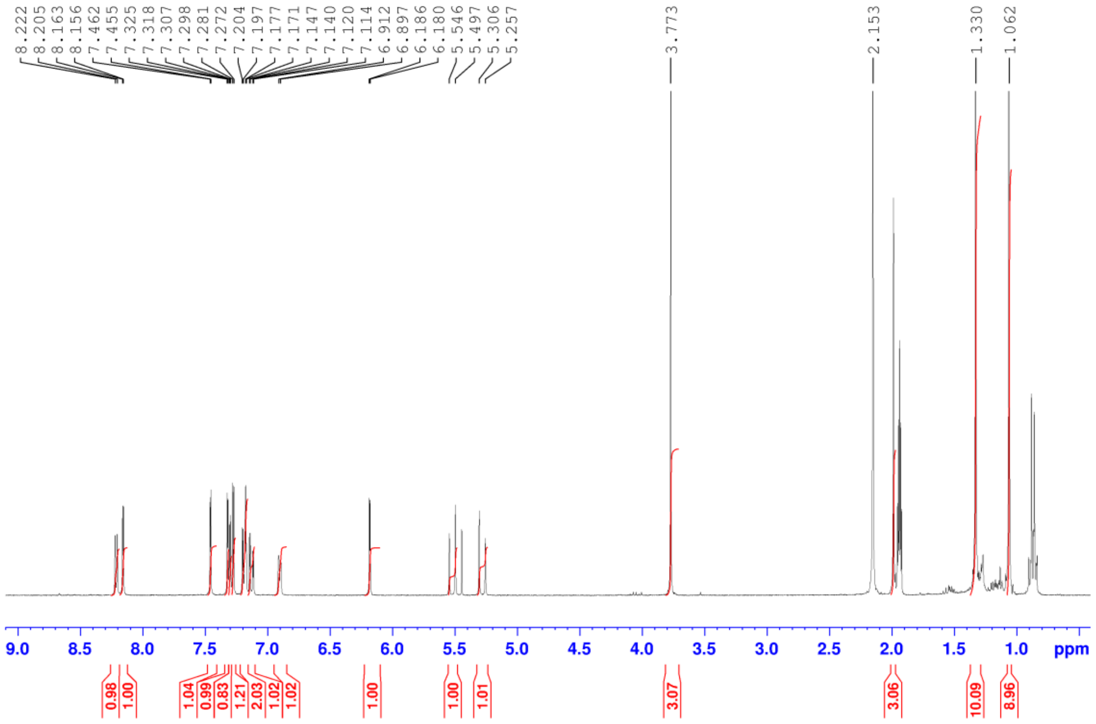

Figure S56: ^{1}H NMR of complex **BGC14a** in CD_3CN, recorded at 300 MHz and 300 K

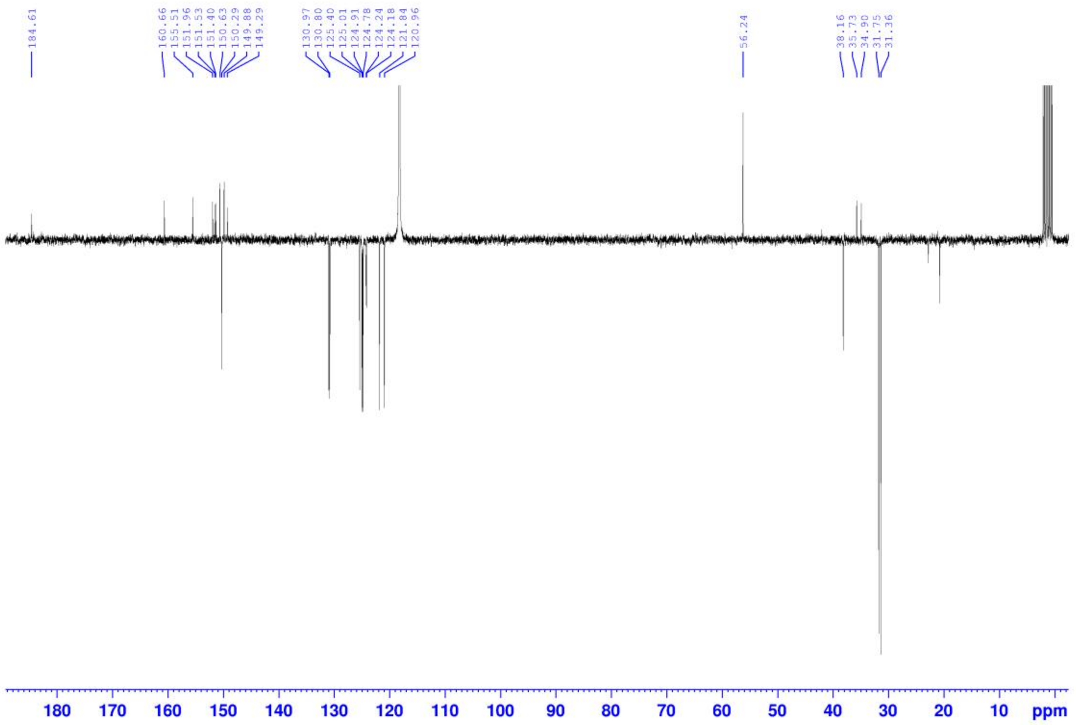

Figure S57: $^{13}C\{^{1}H\}$ JMod NMR of complex **BGC14a** in CD_3CN, recorded at 75 MHz and 300 K

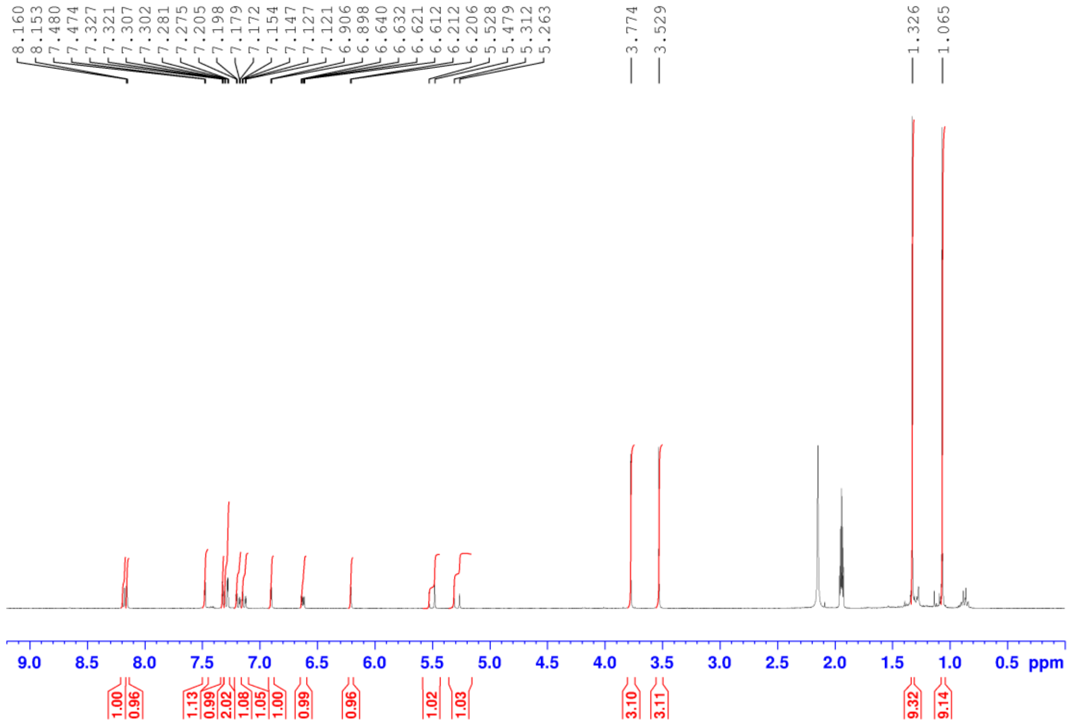

Figure S58: ^{1}H NMR of complex **BGC15a** in CD_3CN, recorded at 300 MHz and 300 K

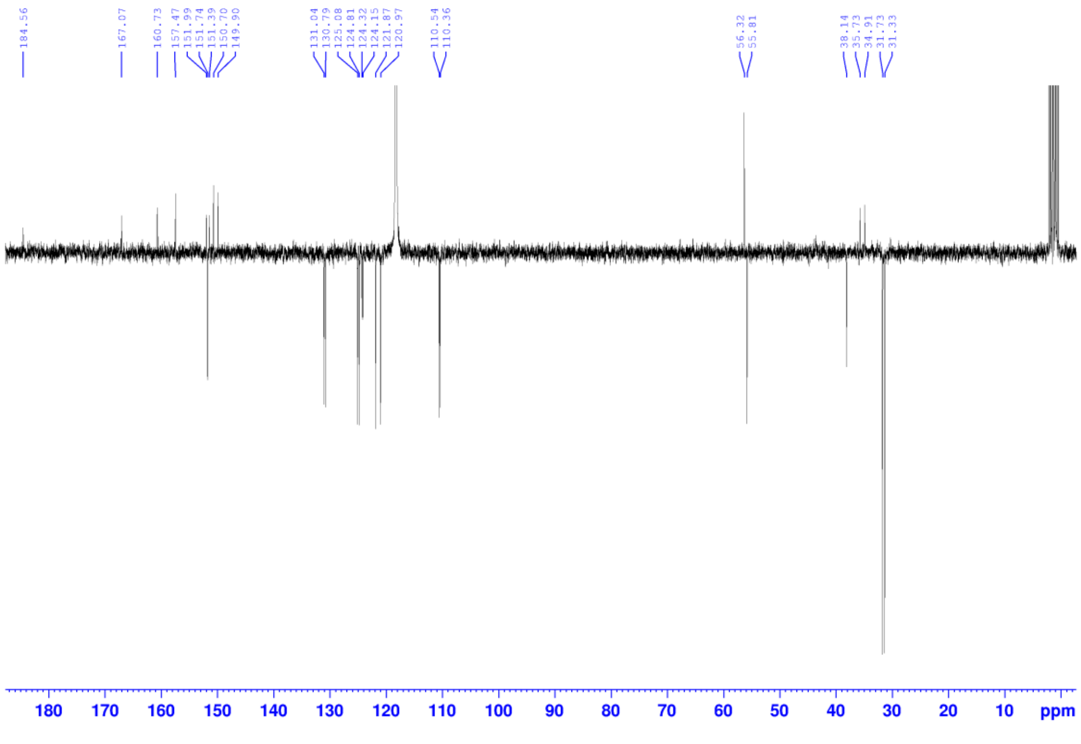

Figure S59: $^{13}C\{^{1}H\}$ JMod NMR of complex **BGC15a** in CD_3CN, recorded at 75 MHz and 300 K

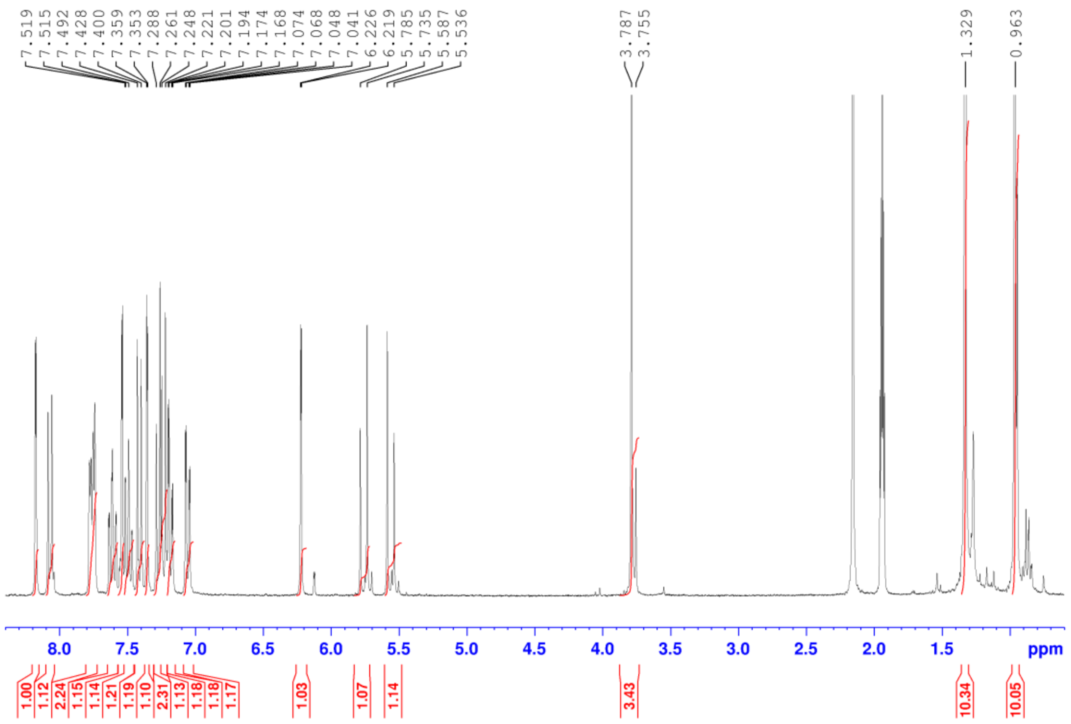

Figure S60: ^{1}H NMR of complex **BGC16a** in CD_3CN, recorded at 300 MHz and 300 K

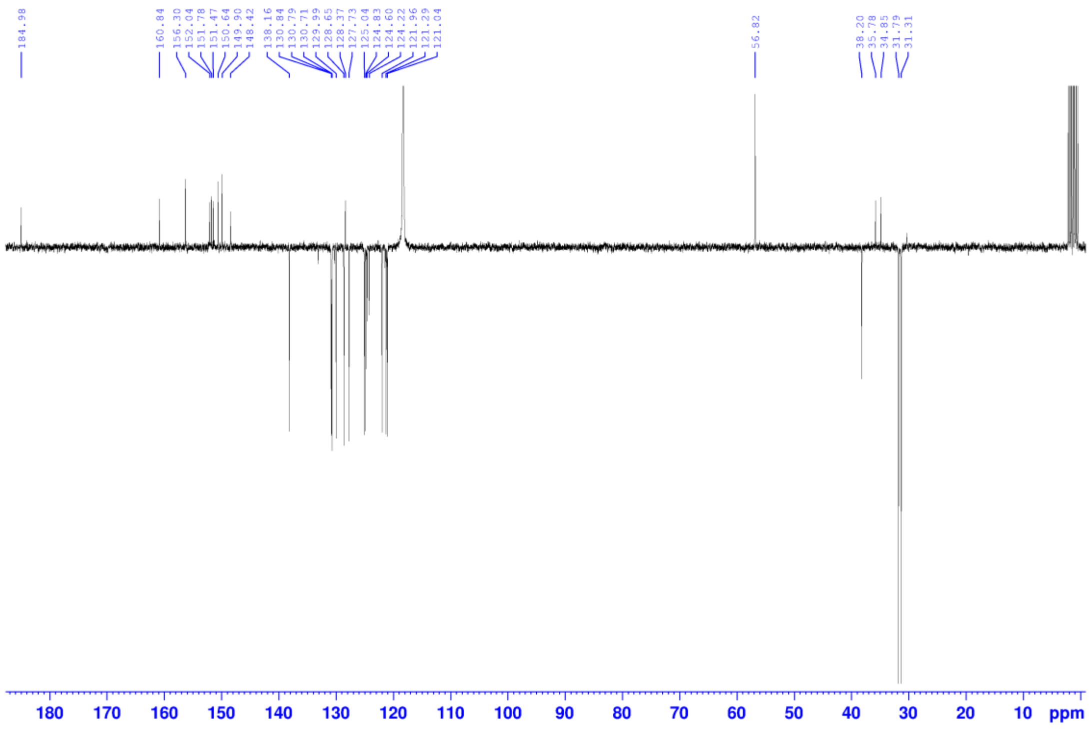

Figure S61: $^{13}C\{^1H\}$ JMod NMR of complex **BGC16a** in CD_3CN, recorded at 75 MHz and 300 K

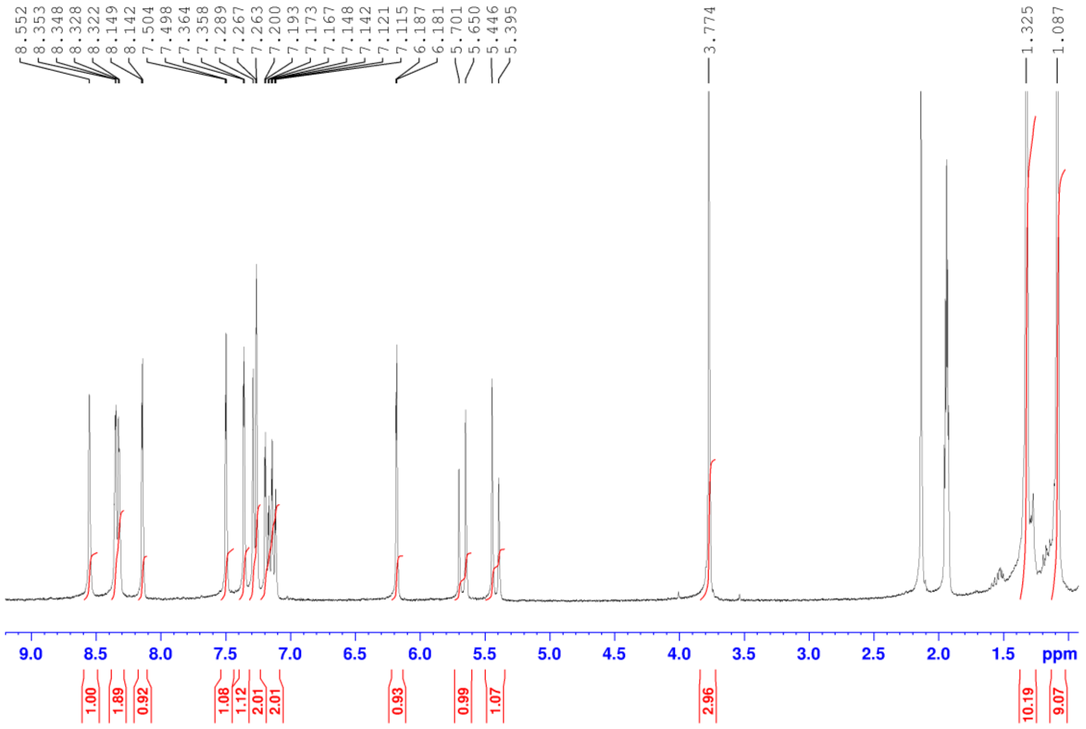

Figure S62: ^{1}H NMR of complex **BGC17a** in CD_3CN, recorded at 300 MHz and 300 K

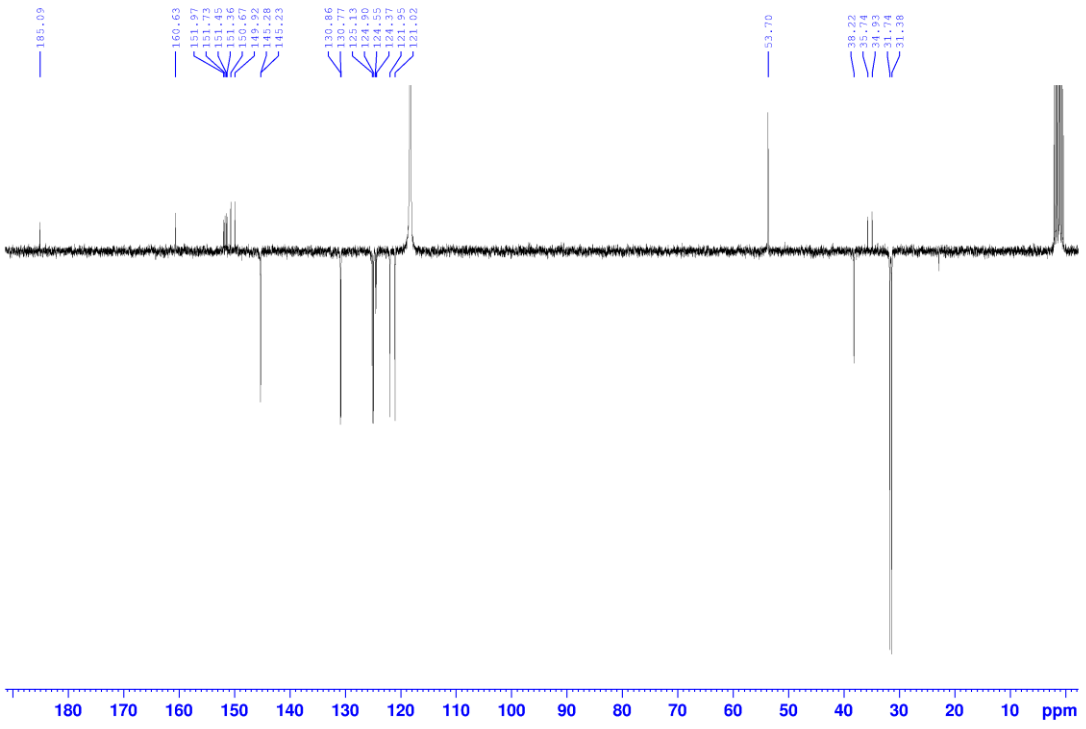

Figure S63: $^{13}C\{^{1}H\}$ JMod NMR of complex **BGC17a** in CD_3CN, recorded at 75 MHz and 300 K

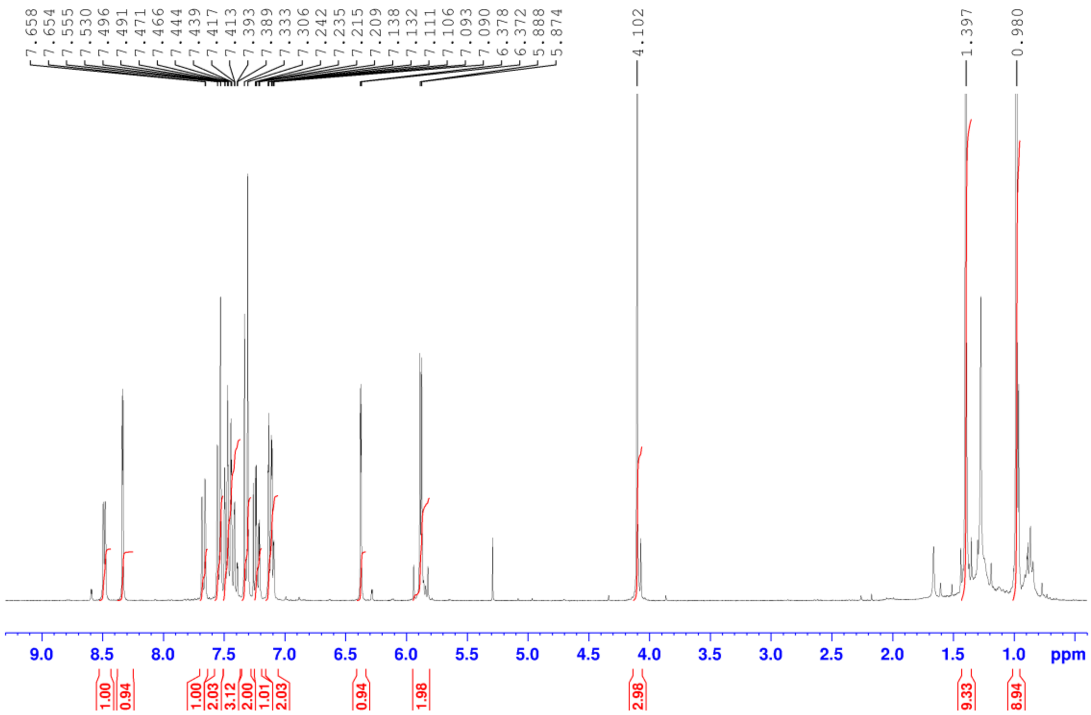

Figure S64: ^{1}H NMR of complex **BGC18a** in $CDCl_3$, recorded at 300 MHz and 300 K

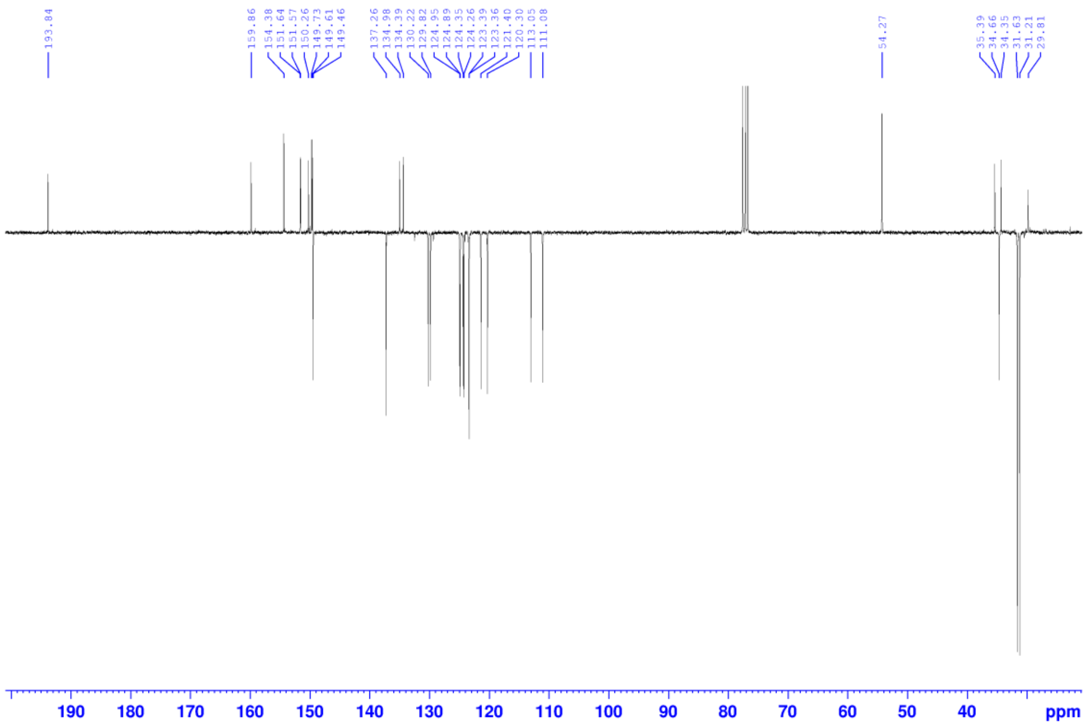

Figure S65: $^{13}C\{^{1}H\}$ JMod NMR of complex **BGC18a** in $CDCl_3$, recorded at 75 MHz and 300 K

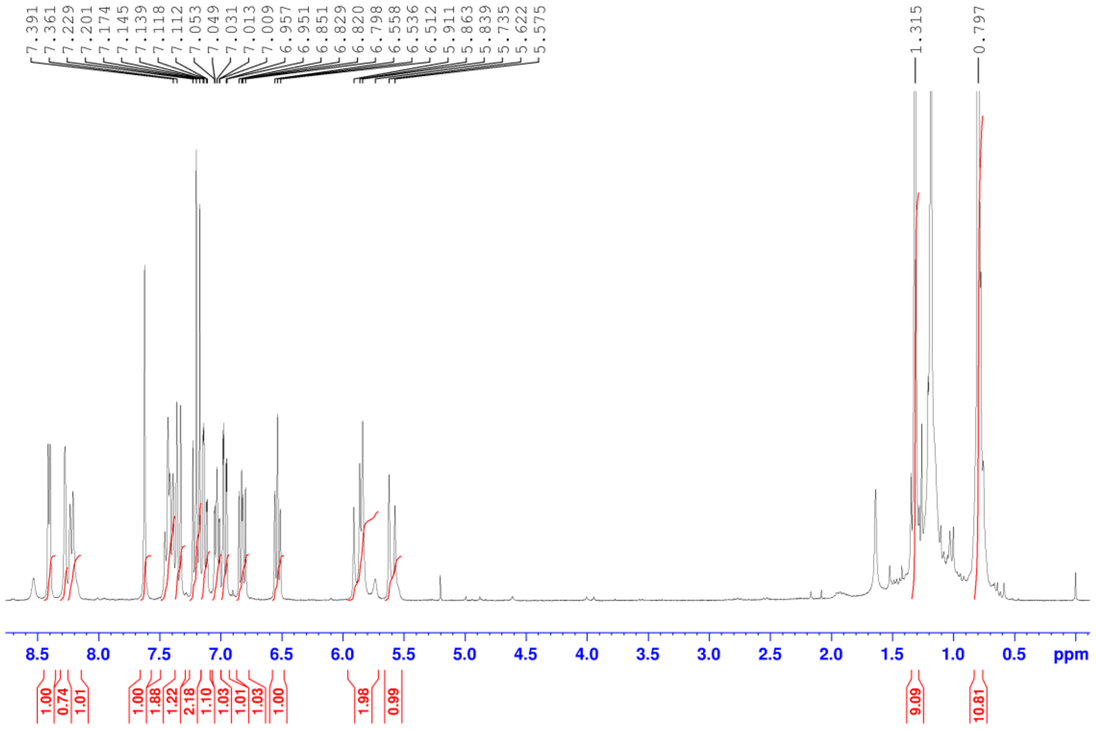

Figure S66: ^{1}H NMR of complex **BGC19a** in CD_3CN, recorded at 300 MHz and 300 K

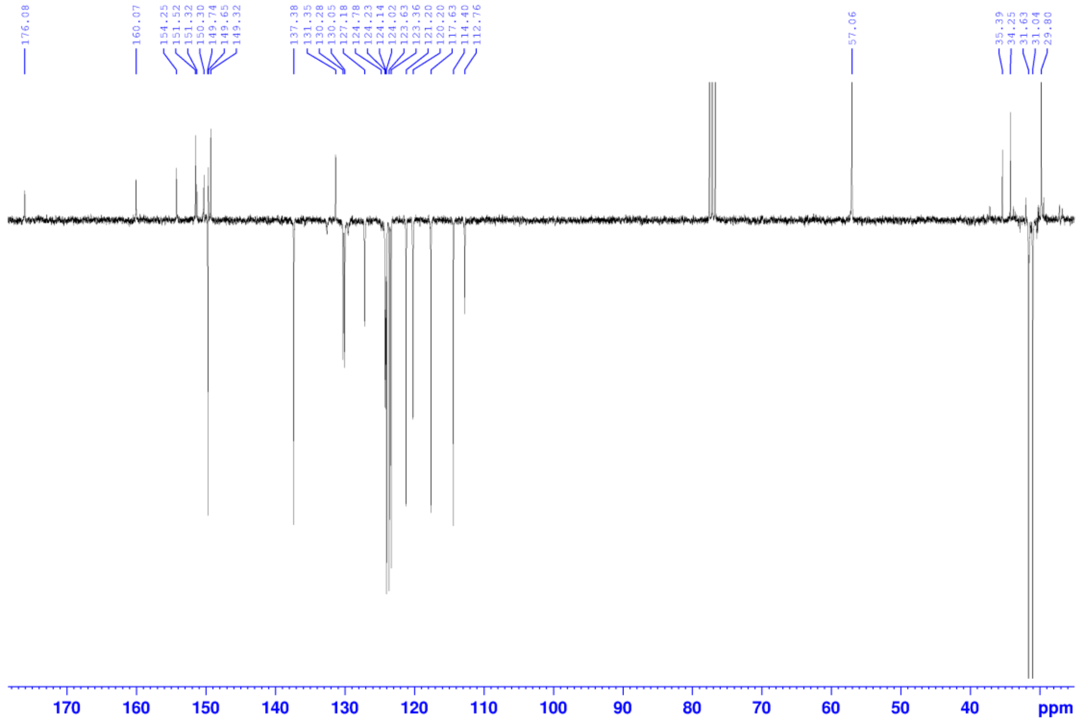

Figure S67: ^{13}C{^{1}H} JMod NMR of complex **BGC19a** in CD_3CN, recorded at 75 MHz and 300 K

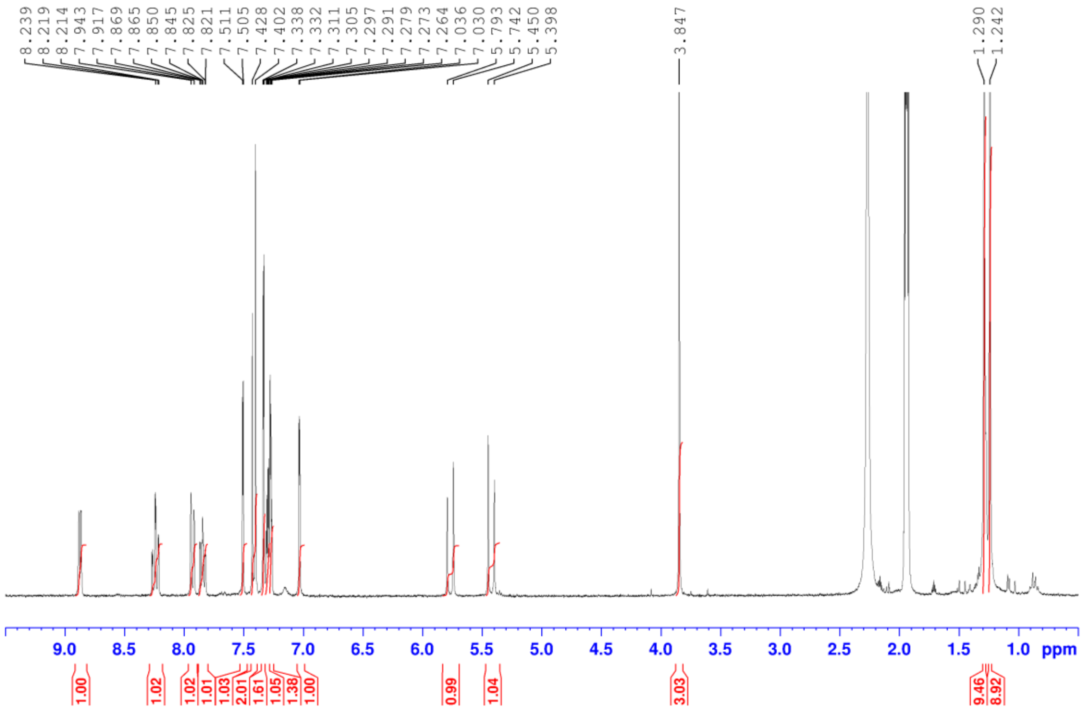

Figure S68: ^{1}H NMR of complex **BGC12b** in CD_3CN, recorded at 300 MHz and 300 K

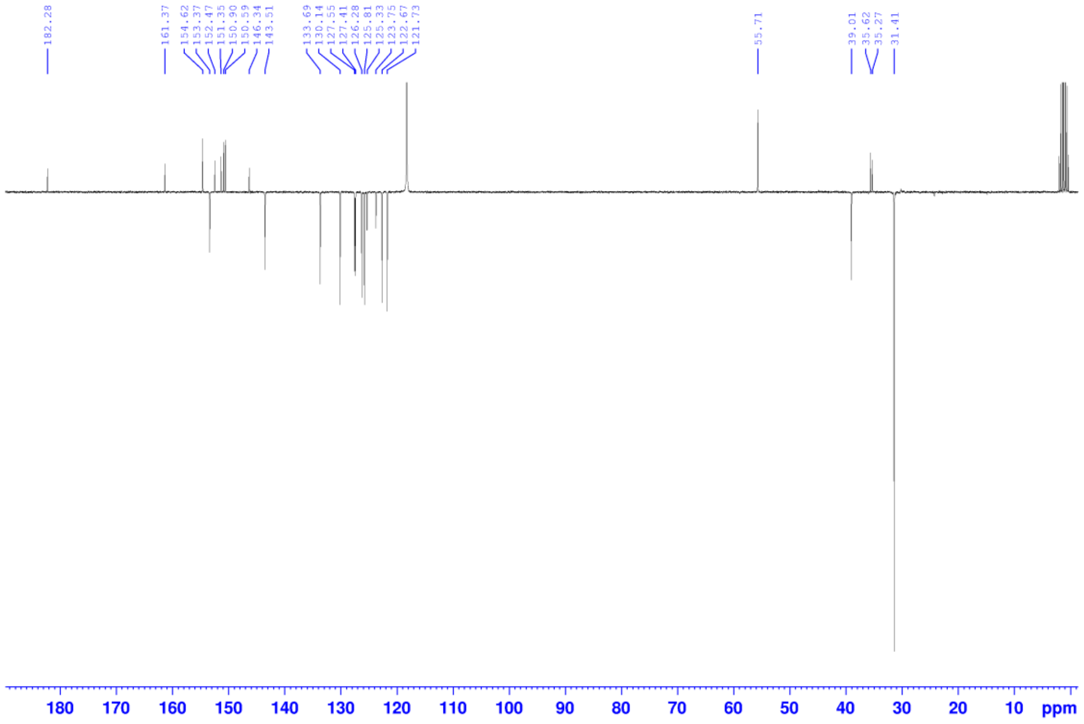

Figure S69: ^{13}C{^{1}H} JMod NMR of complex **BGC12b** in CD_3CN, recorded at 75 MHz and 300 K

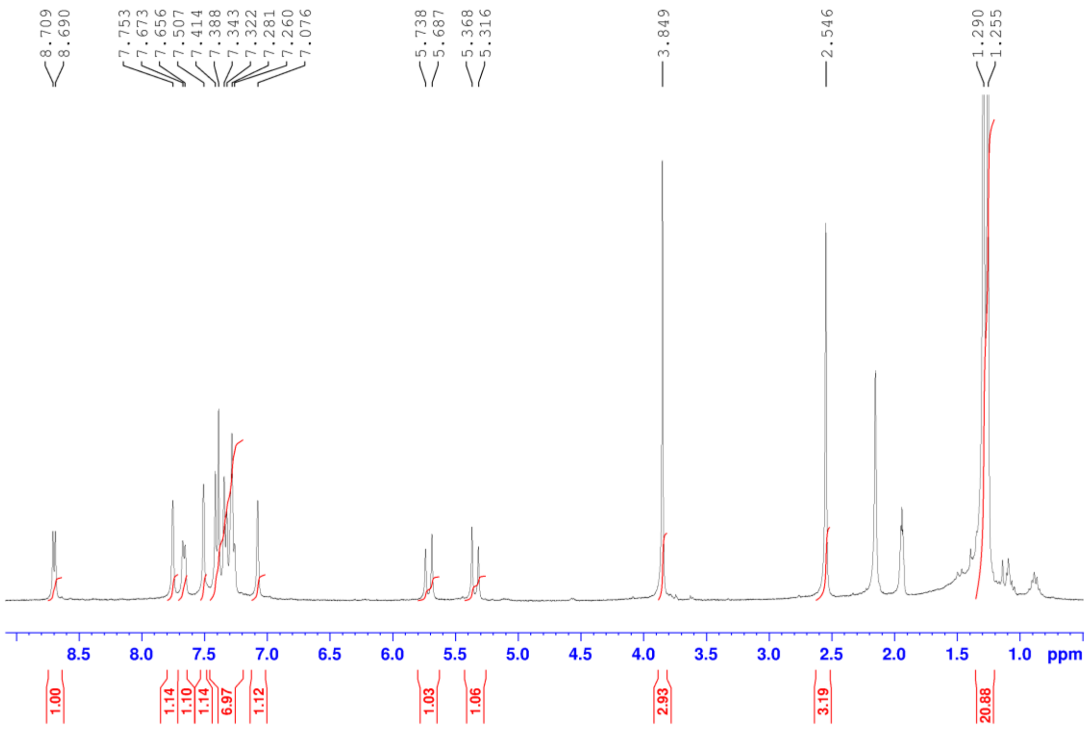

Figure S70: ^{1}H NMR of complex **BGC13b** in CD_3CN, recorded at 300 MHz and 300 K

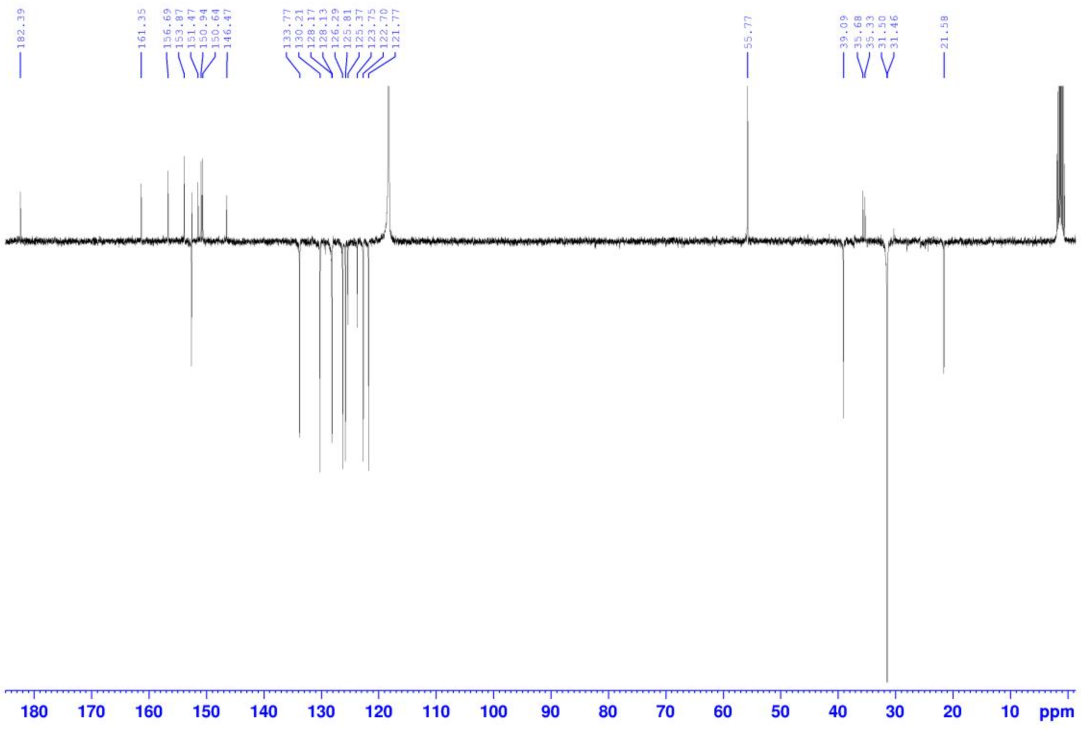

Figure S71: $^{13}C\{^{1}H\}$ JMod NMR of complex **BGC13b** in CD_3CN, recorded at 101 MHz and 300 K

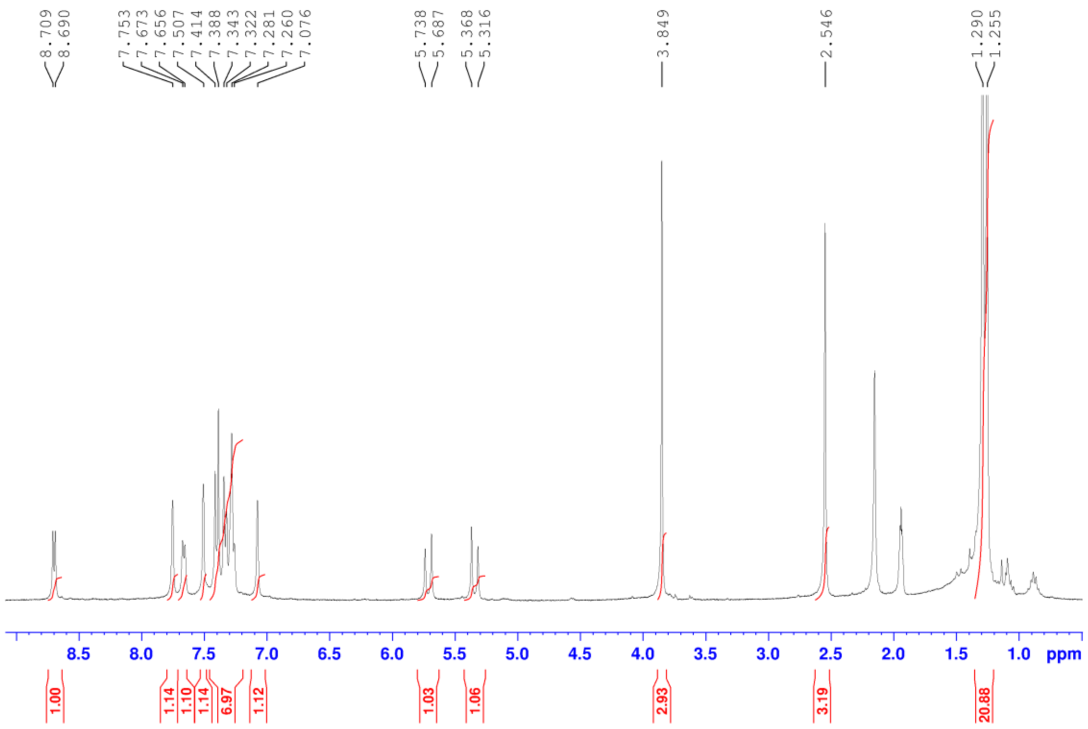

Figure S72: ^{1}H NMR of complex **BGC14b** in CD_3CN, recorded at 300 MHz and 300 K

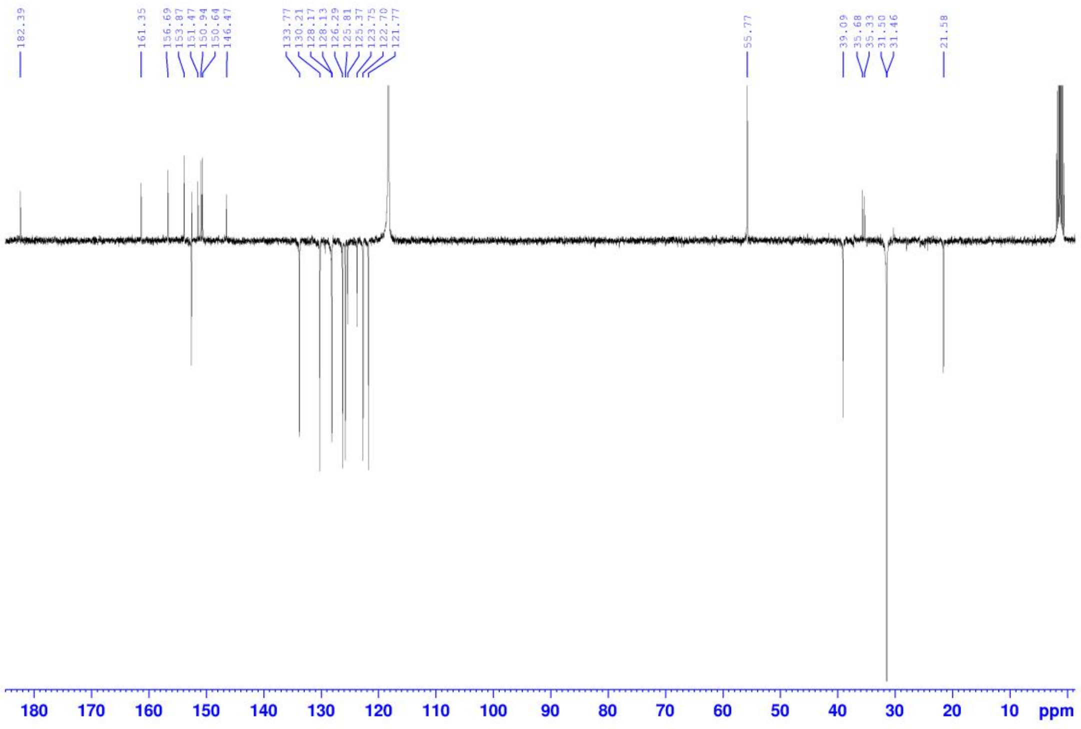

Figure S73: $^{13}C\{^{1}H\}$ JMod NMR of complex **BGC14b** in CD_3CN, recorded at 75 MHz and 300 K

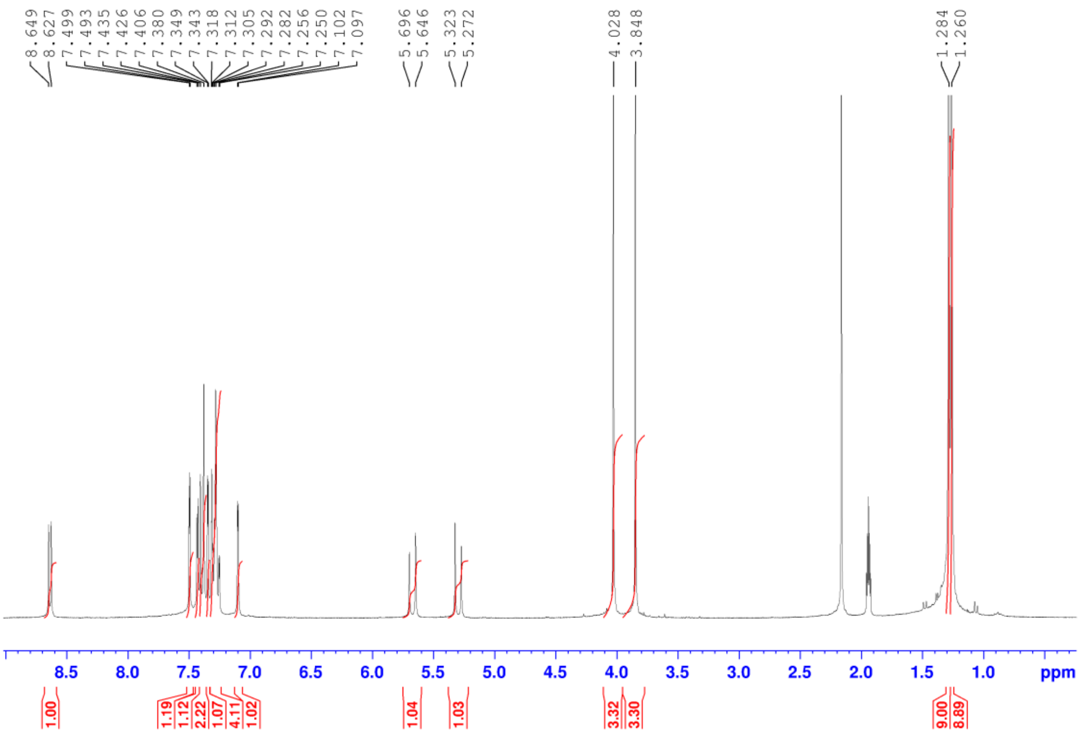

Figure S74: ^{1}H NMR of complex **BGC15b** in CD_3CN, recorded at 300 MHz and 300 K

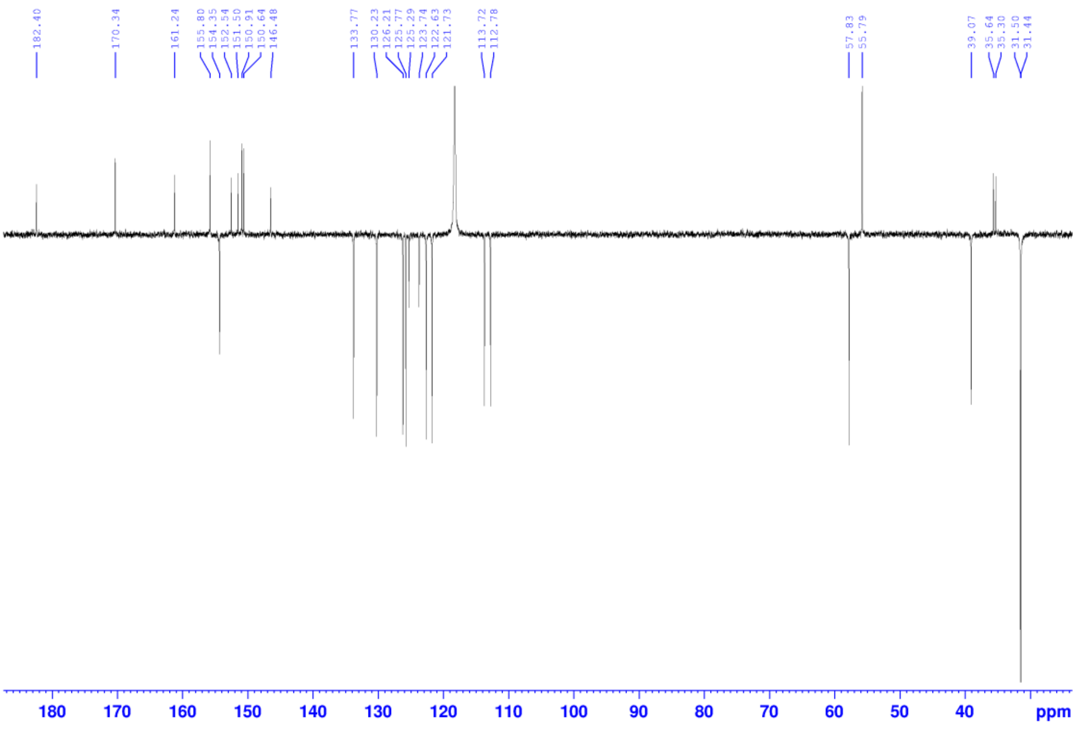

Figure S75: $^{13}C\{^{1}H\}$ JMod NMR of complex **BGC15b** in CD_3CN, recorded at 75 MHz and 300 K

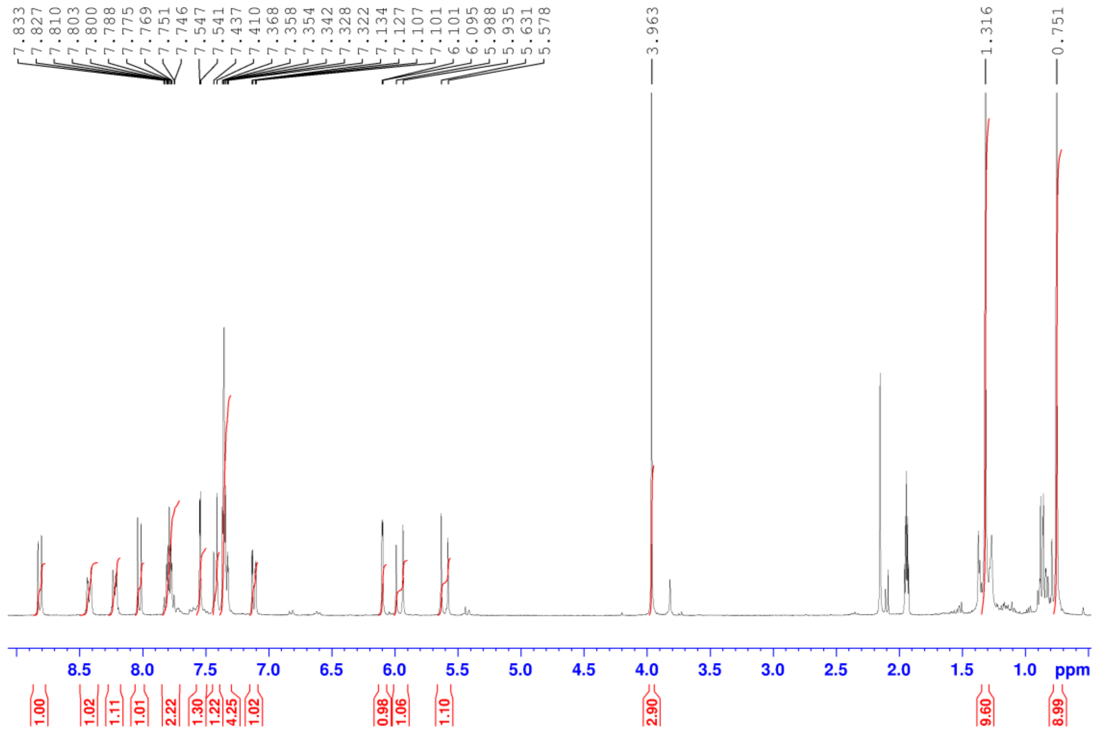

Figure S76: ^{1}H NMR of complex **BGC16b** in CD_3CN, recorded at 300 MHz and 300 K

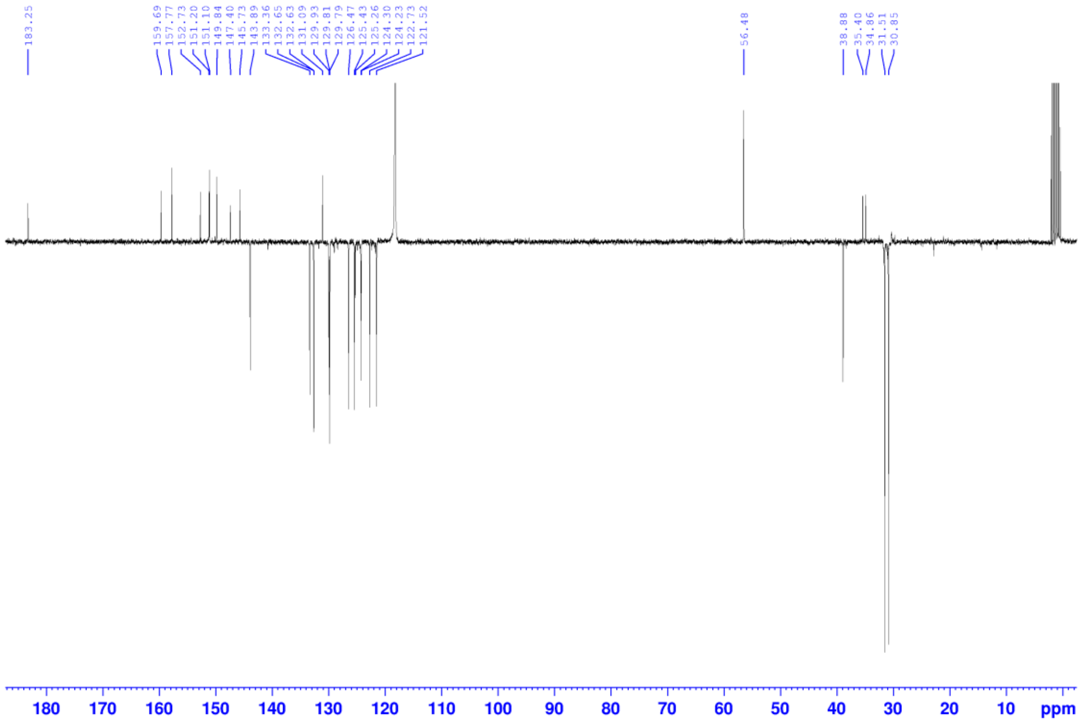

Figure S77: $^{13}C\{^{1}H\}$ JMod NMR of complex **BGC16b** in CD_3CN, recorded at 75 MHz and 300 K

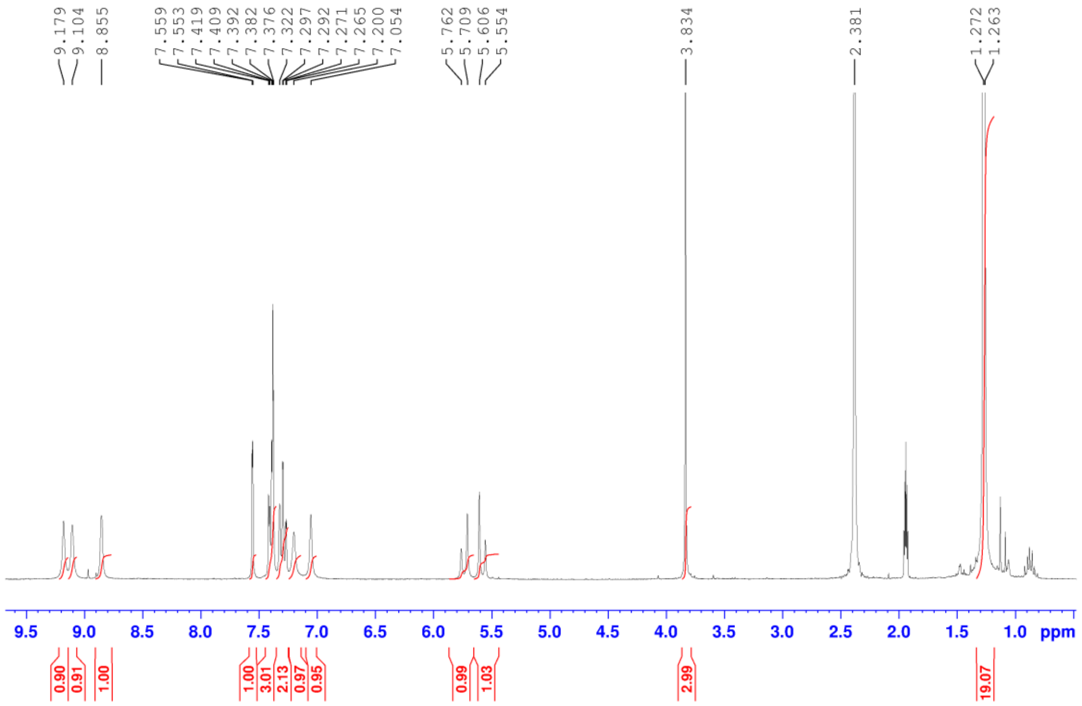

Figure S78: ^{1}H NMR of complex **BGC17b** in CD_3CN, recorded at 300 MHz and 300 K

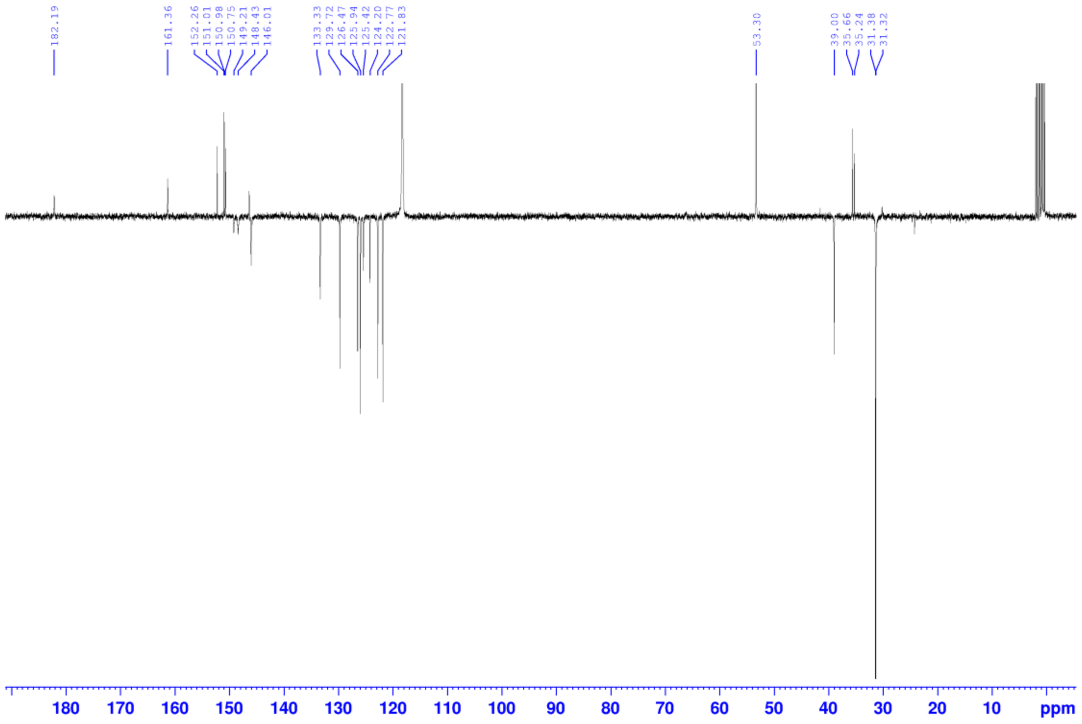

Figure S79: $^{13}C\{^{1}H\}$ JMod NMR of complex **BGC17b** in CD_3CN, recorded at 75 MHz and 300 K

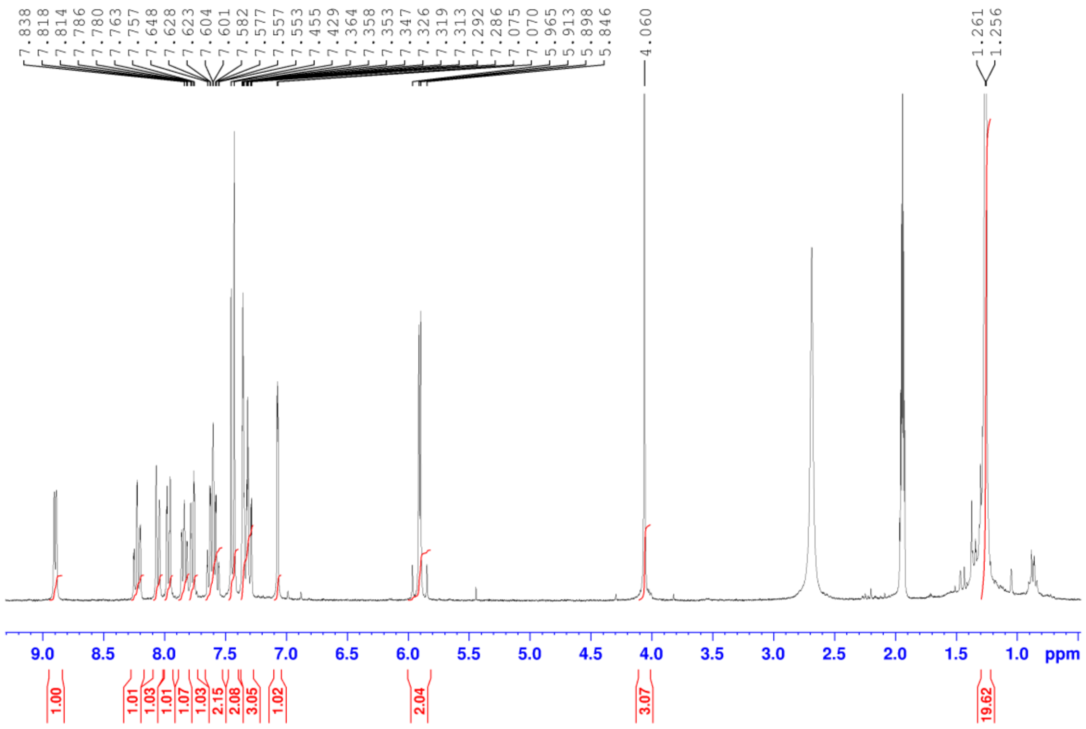

Figure S80: ^{1}H NMR of complex **BGC18b** in CD_3CN, recorded at 300 MHz and 300 K

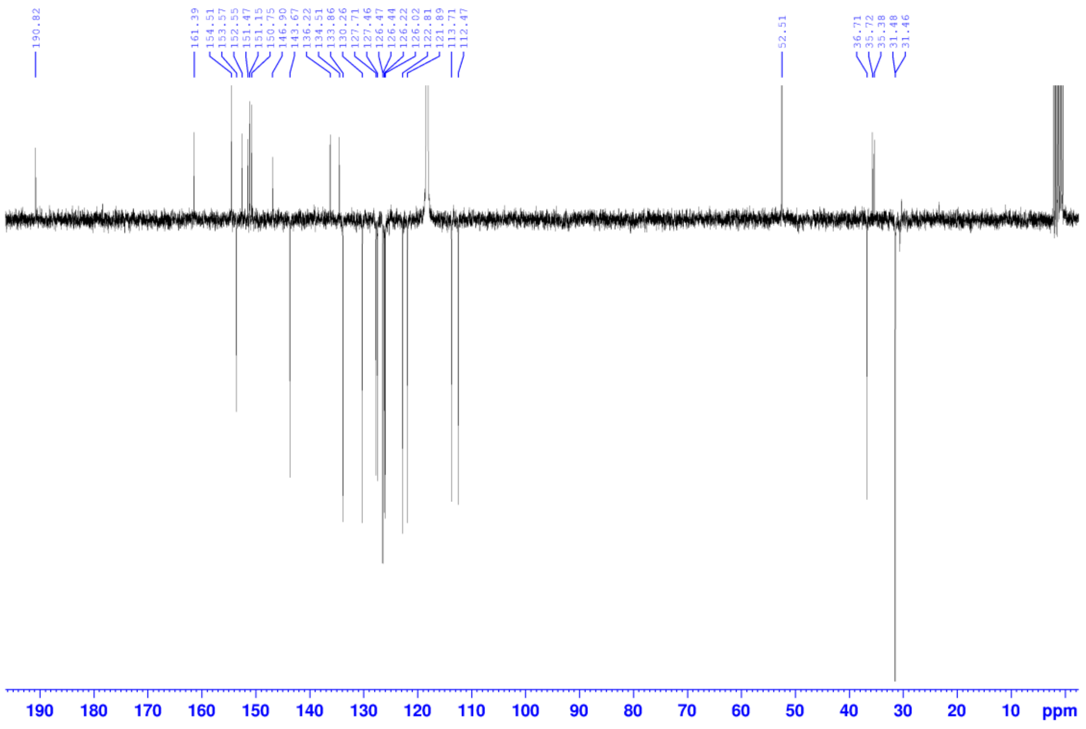

Figure S81: $^{13}C\{^{1}H\}$ JMod NMR of complex **BGC18b** in CD_3CN, recorded at 75 MHz and 300 K

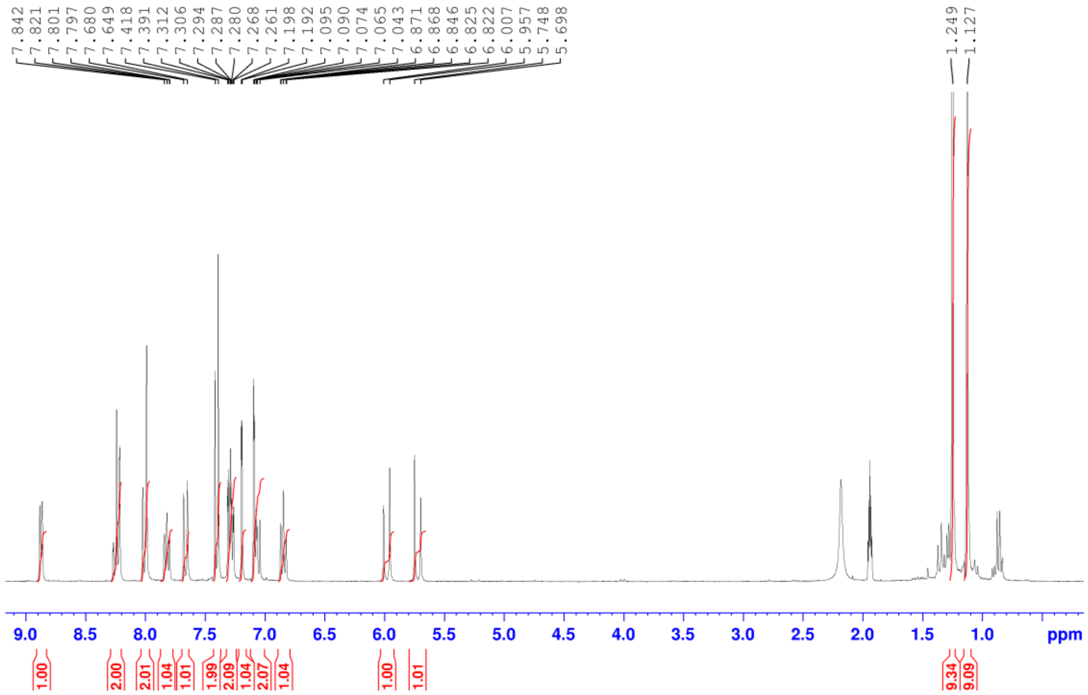

Figure S82: ^{1}H NMR of complex **BGC19b** in CD_3CN, recorded at 300 MHz and 300 K

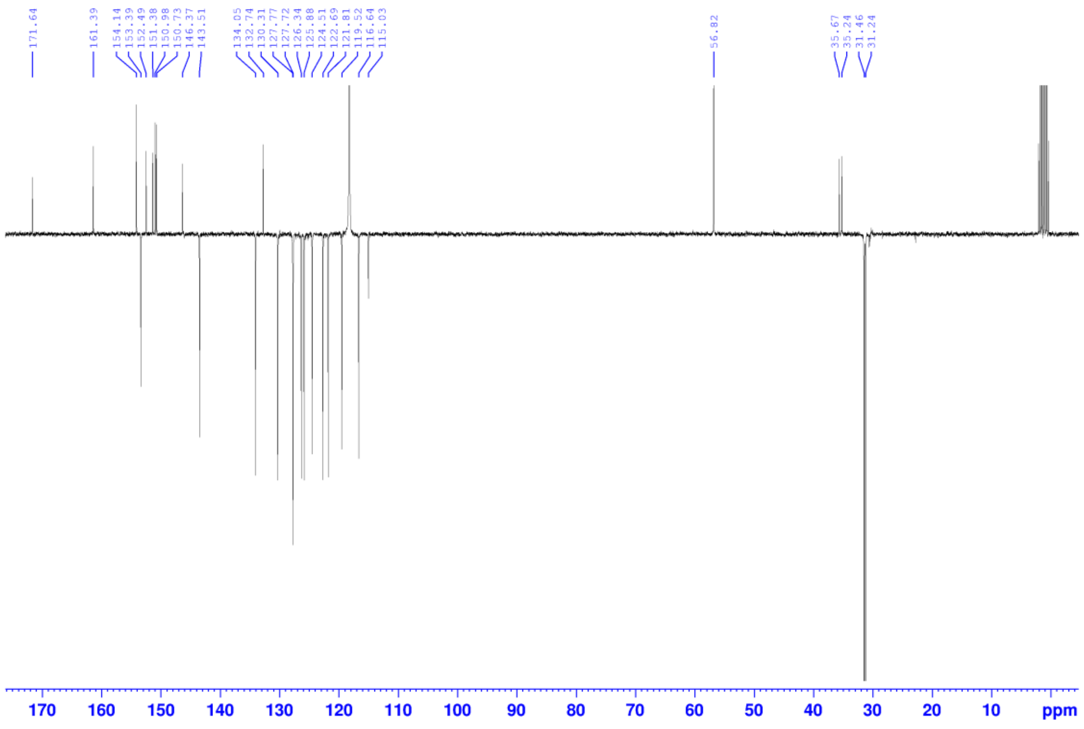

Figure S83: $^{13}C\{^{1}H\}$ JMod NMR of complex **BGC19b** in CD_3CN, recorded at 75 MHz and 300 K

Table S5. Crystallographic data for **BGC12a**, **BGC16a**, **BGC18a** and **BGC13b**, **BGC16b**, **BGC18b**.

	**BGC12a**	**BGC16a**	**BGC18a**
CCDC deposit number	*2420678*	*2420679*	*2420680*
**Empirical formula**[a]	$C_{31.13}H_{37.25}AuCl_{3.25}N_3$ [+ $C_{0.875}H_{1.75}Cl_{1.75}$]	$C_{69.5}H_{76.25}Au_2Br_{0.2}Cl_{1.8}N_{6.75}$	$C_{34}H_{37}AuClN_3$
**Moiety Formula**	$C_{30}H_{35}AuClN_3$, 0.25 (CH_2Cl_2), 0.875 [CH_2Cl_2]	$C_{34}H_{37}AuClN_3$, $C_{34}H_{37}AuBr_{0.2}Cl_{0.8}N_3$, 0.75 ($C_2H_3N$)	$C_{34}H_{37}AuClN_3$
**Formula weight (g/mol)**	765.63 [+ 74.32]	1479.84	720.08
**Temperature (K)**	200	200	200
**Crystal system**	Tetragonal	Monoclinic	Triclinic
**Space group**	$I4_1$	$P2_1/n$	P-1
**a (Å)**	26.6396(5)	25.2400(5)	11.2750(5)
**b (Å)**	26.6396(5)	10.63800(10)	12.3974(6)
**c (Å)**	18.7006(10)	26.0135(5)	13.2544(7)
**α (°)**	90	90	115.340(2)
**β (°)**	90	109.667(2)	97.718(2)
**γ (°)**	90	90	105.802(2)
**Volume (Å^3)**	13271.2(9)	6577.2(2)	1542.68(13)
**Z**	16	4	2
**ρ_{calc} (g/cm^3)**	1.533 [+ 0.149]	1.494	1.550

	**BGC13b**	**BGC16b**	**BGC18b**
CCDC deposit number	*2420681*	*2420683*	*2420682*
**Empirical formula**[a]	$C_{31}H_{37}AuF_6N_3P$	$C_{34.5}H_{38}AuClF_6N_3P$	$C_{34}H_{37}AuF_6N_3P$
**Moiety Formula**	$C_{31}H_{37}AuN_3^+$, PF_6^-	$C_{34}H_{37}AuN_3^+$, PF_6^-, 0.5 (CH_2Cl_2)	$C_{34}H_{37}AuN_3^+$, PF_6^-
**Formula weight (g/mol)**	793.57	872.06	829.60
**Temperature (K)**	200	200	200
**Crystal system**	Monoclinic	Triclinic	Monoclinic
**Space group**	$P2_1/c$	P-1	C2/c
**a (Å)**	20.9251(17)	10.4268(16)	27.6082(11)
**b (Å)**	16.3535(17)	12.2155(19)	9.1293(4)
**c (Å)**	9.1563(12)	13.971(2)	28.6297(12)
**α (°)**	90	92.255(2)	90
**β (°)**	97.973(9)	102.908(2)	113.267(2)
**γ (°)**	90	99.460(2)	90
**Volume (Å^3)**	3103.0(6)	1705.5(5)	6629.1(5)
**Z**	4	2	8
**ρ_{calc} (g/cm^3)**	1.699	1.698	1.662

[a] Including solvent molecules (if presence)

[b] $R1 = \sum||F_o| - |F_c|| / \sum |F_o|$ [c] $wR2 = \sqrt{\sum(w(F_o^2 - F_c^2))/\sum(w(F_o^2)^2)}$
